# Supplementary material for: Simultaneous alleviation of verification and reference standard biases in a community-based tuberculosis screening study using Bayesian latent class analysis
Source: PLoS One. 2024 Jun 10;19(6):e0305126. doi: 10.1371/journal.pone.0305126 (PMC11164341; doi:10.1371/journal.pone.0305126)
Supplement: S1 File — Appendix A presents additional results based on the analysis of Vukuzazi dataset. Appendix B presents the true values of the simulated data and an additional analysis of the simulated data. Appendix C presents a detailed derivation of the Bayesian latent class model, model structure and the associated parameters as well as the priors. In this appendix, we also present a technical note on how we imputed the missing data in Vukuzazi study using MICE. (DOCX) [file pone.0305126.s001.docx]

**Simultaneous alleviation of verification and reference standard biases in a community-based tuberculosis screening study using Bayesian latent class analysis**

**Supplementary Materials**

Alfred Kipyegon Keter^1, 2, 3, a*^, Fiona Vanobberghen^4, 5^, Lutgarde Lynen^1^, Alastair Van Heerden^2, 6^, Jana Fehr^7, 8^, Stephen Olivier^7^, Emily B. Wong^7, 9^, Tracy R Glass^4, 5^, Klaus Reither^4, 5^, Els Goetghebeur^3^, Bart K.M Jacobs^1^

^1^ Institute of Tropical Medicine, Antwerp, Belgium

^2^ Center for Community Based Research, Human Sciences Research Council, Pietermaritzburg, South Africa

^3^ Ghent University, Belgium

^4^ Swiss Tropical and Public Health Institute, Switzerland

^5^ University of Basel, Basel, Switzerland

^6^ SAMRC/WITS Developmental Pathways for Health Research Unit, Department of Paediatrics, School of Clinical Medicine, Faculty of Health Sciences, University of the Witwatersrand, Johannesburg, Gauteng, South Africa

^7^ Africa Health Research Institute, South Africa

^8^ Hasso-Plattner-Institute for Digital Engineering, Potsdam, Germany

^9^ University of Alabama at Birmingham, USA

* Corresponding Author

E-mail: [keteralfred@gmail.com](mailto:keteralfred@gmail.com) (AKA)

**Appendix A: Vukuzazi study**

S1 Fig describes the enrolled participants (n=9914), exclusions, and participants included in the analysis (n=9869). Among these, the figure describes the participants who were eligible for bacteriological testing for PTB using Xpert Ultra and culture and were tested (n=4942), those who were eligible but were not tested (n=1427), and those who were not eligible for bacteriological testing (n=3500). We included all 9869 participants in the analysis to estimate the prevalence of pulmonary TB in the three groups.


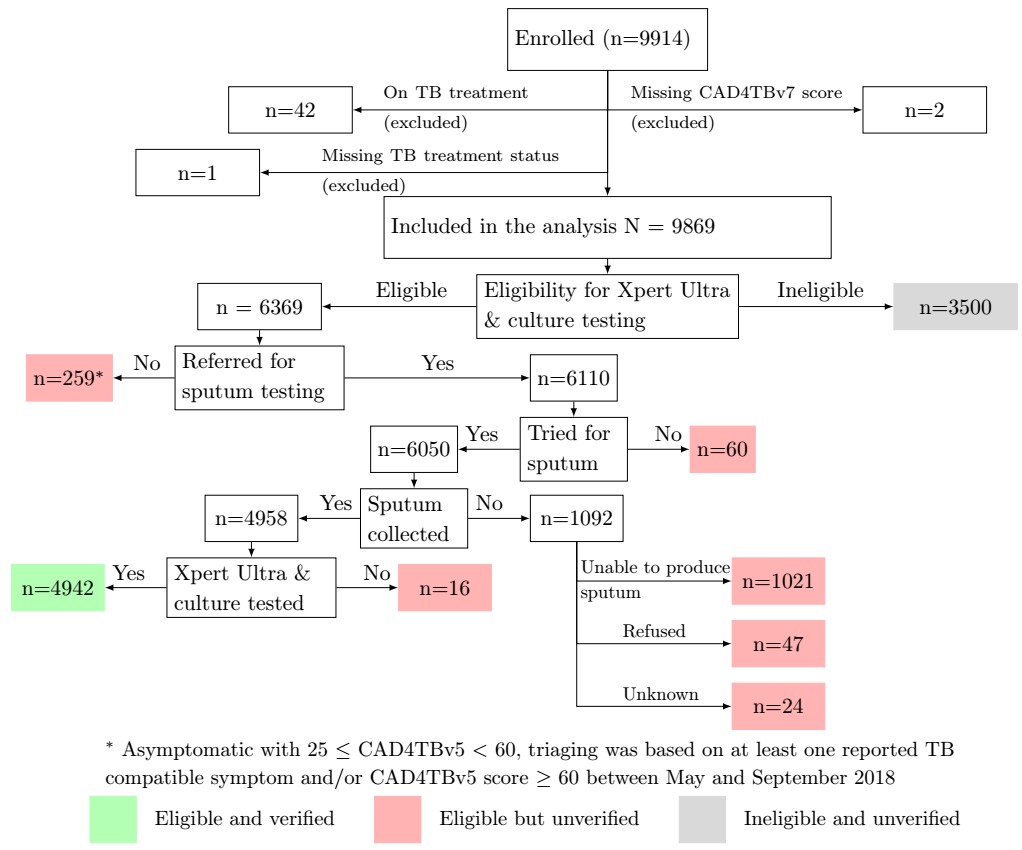


**S1 Fig: Consort diagram depicting the eligibility of the participants for bacteriological testing and inclusion in the analysis. Eligibility for Xpert Ultra and culture testing was based on at least one reported TB-compatible symptom and/or CAD4TBv5 score ≥25. Criterion of at least one reported TB-compatible symptom and/or CAD4TBv5 score ≥60 was used between May and September 2018.**

**S1 Table: Classification of diagnostic test results by bacteriological testing status from Vukuzazi study**

|  |  |  |  |  | $Y_{1}-$ |  |  |  | $Y_{1}+$ |  |  |
| --- | --- | --- | --- | --- | --- | --- | --- | --- | --- | --- | --- |
|  |  |  | $Y_{2}-$ | | $Y_{2}+$ | | $Y_{2}-$ | | $Y_{2}+$ | |  |
| V | $Y_{5}$ | $Y_{6}$ | $Y_{3}<25$ | $Y_{3}\geq25$ | $Y_{3}<25$ | $Y_{3}\geq25$ | $Y_{3}<25$ | $Y_{3}\geq25$ | $Y_{3}<25$ | $Y_{3}\geq25$ | Total |
|  | $-$ | $-$ | 0 | 2688 | 137 | 1223 | 239 | 343 | 14 | 231 | 4875 |
|  | $-$ | $+$ | 0 | 6 | 1 | 13 | 1 | 1 | 0 | 4 | 26 |
| TT | $+$ | $-$ | 0 | 1 | 0 | 12 | 0 | 0 | 0 | 3 | 16 |
|  | $+$ | $+$ | 0 | 2 | 0 | 20 | 0 | 0 | 0 | 3 | 25 |
| NT |  |  | 3500 | 972 | 38 | 211 | 67 | 82 | 3 | 54 | 4927 |
| Total |  |  | 3500 | 3669 | 176 | 1479 | 307 | 426 | 17 | 295 | 9869 |

$Y_{1}$ - Any TB symptom, $Y_{2}$ – Radiologist interpretation (any chest X-ray abnormality), $Y_{3}$ – CAD4TBv5, $Y_{5}$ – Xpert Ultra (excluding trace), $Y_{6}$ – Culture, V – Bacteriological testing status; “$-$ “= Negative test result; “$+$” = positive test result; NT = Not Bacteriologically tested; TT = Bacteriologically tested


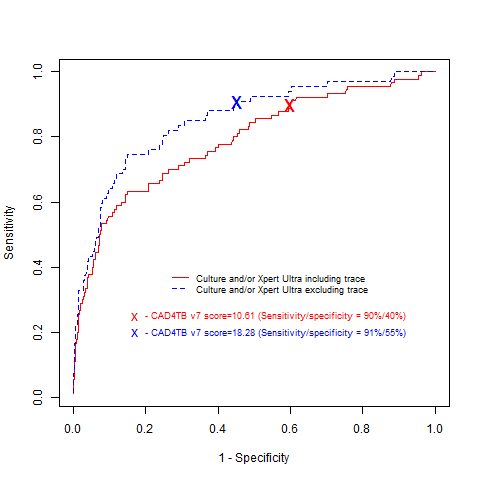


**S2 Fig: Receiver operating characteristic curve for CAD4TB version 7 against a composite reference standard of Xpert Ultra and/or culture**

**S2 Table: Comparison of the different groups of eligible and unverified, eligible but unverified and ineligible and unverified participants by measured participant characteristics**

|  | Eligibility for bacteriological testing using Xpert Ultra and culture and the testing tatus | | |  |
| --- | --- | --- | --- | --- |
| Characteristic | Eligible and verified  (n=4942; 50.1%) | Eligible but unverified  (n=1427; 14.5%) | Ineligible and untested (n=3500; 35.5%) | Total  (n=9869) |
| Age (Years) |  |  |  |  |
| 15 – 29 | 1153 (23.3%) | 484 (33.9%) | 1838 (52.5%) | 3475 (35.2%) |
| 30 – 49 | 1278 (25.9%) | 321 (22.5%) | 1125 (32.1%) | 2724 (27.6%) |
| 50 – 69 | 1717 (34.8%) | 411 (28.8%) | 481 (13.7%) | 2609 (26.4%) |
| ≥70 | 793 (16.0%) | 211 (14.8%) | 56 (1.6%) | 1060 (10.7%) |
| Missing | 1 | 0 | 0 | 1 |
| Sex |  |  |  |  |
| Female | 3144 (63.6%) | 546 (38.3%) | 883 (25.2%) | 6642 (67.3%) |
| Male | 1798 (36.4%) | 881 (61.7%) | 2617 (74.8%) | 3227 (32.7%) |
| HIV status |  |  |  |  |
| Negative | 3454 (70.1%) | 1068 (75.7%) | 2368 (68.0%) | 6890 (70.2%) |
| Positive | 1474 (29.9%) | 342 (24.3%) | 1112 (32.0%) | 2928 (29.8%) |
| Missing | 14 | 17 | 20 | 51 |
| Any TB symptom^†^ |  |  |  |  |
| No | 4103 (83.0%) | 1221 (85.6%) | 3500 (100%) | 8824 (89.4%) |
| Yes | 839 (17.0%) | 206 (14.4%) | 0 (0.0%) | 1045 (10.6%) |
| Chest X-ray lung field findings |  |  |  |  |
| Normal | 3281 (66.4%) | 1121 (78.6%) | 3500 (100%) | 7902 (80.1%) |
| Abnormal, not suggestive of active TB | 1495 (30.3%) | 283 (19.8%) | 0 (0.0%) | 1778 (18.0%) |
| Abnormal, suggestive of active TB | 166 (3.4%) | 23 (1.6%) | 0 (0.0%) | 189 (1.9%) |
| CAD4TBv5≥25 |  |  |  |  |
| No | 393 (8.0%) | 108 (7.6%) | 3500 (100%) | 4001 (40.5%) |
| Yes | 4549 (92.0%) | 1319 (92.4%) | 0 (0.0%) | 5868 (59.5%) |
| CAD4TBv7≥18.28 |  |  |  |  |
| No | 2668 (54.0%) | 741 (51.9%) | 2939 (84.0%) | 6348 (64.3%) |
| Yes | 2274 (46.0%) | 686 (48.1%) | 561 (16.0%) | 3521 (35.7%) |

† - A composite of cough and/or fever and/or night sweats and/or weight loss

**S3 Table: Pairwise correlation between the diagnostic tests used Vukuzazi study**

|  | Y_1_ | ­Y_2_ | Y_4_ | Y_5_ | Y_6_ |
| --- | --- | --- | --- | --- | --- |
| Y_1_ | 1.00 | -0.03 | 0.00 | 0.00 | -0.01 |
| Y_2_ | -0.03 | 1.00 | 0.28 | 0.10 | 0.11 |
| Y_4_ | 0.00 | 0.28 | 1.00 | 0.09 | 0.09 |
| Y_5_ | 0.00 | 0.10 | 0.09 | 1.00 | 0.54 |
| Y_6_ | -0.01 | 0.11 | 0.09 | 0.54 | 1.00 |

**Appendix B: Simulation**

S4 Table shows the true values of PTB prevalence (overall and sub-group specific) and the overall true values of diagnostic tests sensitivity and specificity in the simulation.

**S4 Table: True values of PTB prevalence and diagnostic test sensitivity and specificity in the simulation**

| Test | Parameter | True value (%) |
| --- | --- | --- |
|  | PTB prevalence |  |
|  | Eligible and verified | 3.3 |
|  | Eligible but unverified | 2.7 |
|  | Ineligible | 0.1 |
|  | Overall | 2.0 |
| Y_1_ | Sensitivity | 19.8 |
|  | Specificity | 87.4 |
| Y_2_ | Sensitivity | 66.0 |
|  | Specificity | 77.8 |
| Y_3_ | Sensitivity | 86.9 |
|  | Specificity | 70.0 |
| Y_4_ | Sensitivity | 63.8 |
|  | Specificity | 99.3 |
| Y_5_ | Sensitivity | 80.6 |
|  | Specificity | 99.3 |

S5 Table presents the covariances and correlations between the simulated diagnostic tests among the true PTB cases and the true non-PTB cases. The summary is based on all the participants. However, the true covariance and correlation values did not change when restricted to the cases that were verified only. The upper-right triangle presents the correlations while the lower-left triangle presents the covariances between the diagnostic tests. The values in the main diagonal are the true variances of the diagnostic tests. Only pairwise covariances and correlations were considered in the simulations.

**S5 Table: Covariances and correlations between the simulated diagnostic tests among the true PTB cases and the true non-PTB cases**

|  | True PTB cases | | | | |
| --- | --- | --- | --- | --- | --- |
|  | Y_1_ | Y_2_ | Y_4_ | Y_5_ | Y_6_ |
| Y_1_ | 0.158 | 0.034 | 0.055 | -0.017 | -0.001 |
| Y_2_ | 0.006 | 0.223 | **0.480** | 0.003 | 0.005 |
| Y_4_ | 0.008 | 0.077 | 0.114 | 0.006 | -0.008 |
| Y_5_ | -0.003 | 0.001 | 0.001 | 0.229 | **0.471** |
| Y_6_ | 0.000 | 0.001 | -0.001 | 0.089 | 0.154 |
|  | True non-PTB cases | | | | |
|  | Y_1_ | Y_2_ | Y_4_ | Y_5_ | Y_6_ |
| Y_1_ | 0.110 | **0.343** | **0.453** | 0.001 | -0.002 |
| Y_2_ | 0.047 | 0.173 | **0.772** | 0.000 | 0.000 |
| Y_4_ | 0.070 | 0.149 | 0.215 | 0.000 | 0.000 |
| Y_5_ | 0.000 | 0.000 | 0.000 | 0.007 | -0.001 |
| Y_6_ | 0.000 | 0.000 | 0.000 | 0.000 | 0.007 |

S6 Table shows the distribution of the average dataset (average number of subjects within the categories of the multiway table) to illustrate the verification status based on Y_1_, Y­_2_ and Y_3_.

**S6 Table: Distribution of the average of the 100 simulated datasets**

|  |  |  |  |  | $Y_{1}-$ |  |  |  | $Y_{1}+$ |  |  |
| --- | --- | --- | --- | --- | --- | --- | --- | --- | --- | --- | --- |
|  |  |  | $Y_{2}-$ | | $Y_{2}+$ | | $Y_{2}-$ | | $Y_{2}+$ | |  |
| V | $Y_{5}$ | $Y_{6}$ | $Y_{3}<25$ | $Y_{3}\geq25$ | $Y_{3}<25$ | $Y_{3}\geq25$ | $Y_{3}<25$ | $Y_{3}\geq25$ | $Y_{3}<25$ | $Y_{3}\geq25$ | Total |
|  | $-$ | $-$ | 0 | 2528 | 118 | 1001 | 11 | 375 | 0 | 591 | 4624 |
|  | $-$ | $+$ | 0 | 22 | 0 | 24 | 0 | 3 | 0 | 9 | 58 |
| 1 | $+$ | $-$ | 0 | 18 | 1 | 12 | 1 | 3 | 0 | 4 | 39 |
|  | $+$ | $+$ | 0 | 30 | 0 | 39 | 0 | 2 | 0 | 17 | 88 |
| 0 |  |  | 3691 | 883 | 33 | 336 | 6 | 97 | 0 | 145 | 5191 |
| Total |  |  | 3691 | 3481 | 152 | 1412 | 18 | 480 | 0 | 766 | 10000 |

V – Verification status; “$-$ “= Negative test result; “$+$” = positive test result; V = 0 implies not bacteriologically verified; V = 1 implies bacteriologically verified.

**S7 Table: Estimates of overall prevalence based on the analysis using composite reference standard (CRS-based analysis) and Bayesian LCA assuming the unverified participants are negative for bacteriological tests under different assumptions of true prevalence based on a single simulation run with N=10000**

|  | CRS-based analysis | Bayesian LCA |
| --- | --- | --- |
| True value (%) | Median (95% CrI) | Median (95% CrI) |
| 1.0 | 1.2 (1.0-1.5) | 1.4 (1.0-2.2) |
| 2.0 | 2.1 (1.9-2.4) | 2.0 (1.6-2.8) |
| 3.0 | 2.6 (2.3-2.9) | 2.5 (2.0-3.2) |
| 4.0 | 3.1 (2.8-3.5) | 3.4 (2.9-4.3) |
| 5.0 | 3.9 (3.5-4.3) | 3.8 (3.4-4.5) |
| 7.5 | 5.8 (5.4-6.3) | 5.7 (5.1-6.6) |
| 10.0 | 7.3 (6.8-7.8) | 7.3 (6.6-8.2) |
| 15.0 | 10.6 (10.0-11.2) | 10.9 (10.0-12.3) |
| 20.0 | 14.1 (13.4-14.8) | 14.7 (13.7-16.2) |
| 30.0 | 20.7 (20.0-21.5) | 21.6 (20.4-23.6) |

CrI – Credible interval

**Appendix C**

**Model**

Suppose the random variable $Y_{j}, j=1,2,\ldots,J$ denotes the $j^{th}$ diagnostic test and the random variable $D$ denotes the latent (unobserved) disease status such that $Y_{j}=0\left( 1 \right)$ if the $j^{th}$ diagnostic test result is negative (positive) and $D=0\left( 1 \right)$ if the true disease status is negative (positive). Under the assumption of conditional independence, the joint probability of a combination of test results from a set of $J$ diagnostic tests $\boldsymbol{Y}=\left( Y_{1},Y_{2},\cdots,Y_{J} \right)$ is given by [1]

$$Pr\left( \boldsymbol{Y} \right)=\sum_{d=0}^{1} Pr\left( D=d \right)\prod_{j=1}^{J} Pr\left( Y_{j}=y_{j}|D=d \right)\ldots\ldots\ldots\ldots\ldots\ldots\ldots\ldots\ldots\ldots\ldots\ldots\ldots\ldots\left( 1 \right)$$

Suppose that an imperfect diagnostic test considered the most accurate is available. But due to the high cost of performing the test only a subset of individuals who are already suggestive of the presence of the disease based on the first *t* tests receive it. That is, individuals who return positive test results on at least one of the first *t* tests are verified using the most accurate but expensive diagnostic test. Let the random variable V denote the verification status such that V = 1 if an individual is verified and 0 otherwise. Now, suppose that we have data available for a subset of individuals who are verified. Then expression (1) becomes

$$Pr\left( \boldsymbol{Y} | V=1 \right)=\sum_{d=0}^{1} Pr\left( D=d | V=1 \right)\prod_{j=1}^{J} Pr\left( Y_{j}|D=d, V=1 \right)\ldots\ldots\ldots\ldots\ldots\ldots\ldots\ldots\ldots\ldots\ldots\ldots\left( 2 \right)$$

For $J$ dependent diagnostic tests, $Pr\left( \boldsymbol{Y} | V=1 \right)$ can be expressed using the chain rule of conditional probability as follows [2]

$$Pr\left( \boldsymbol{Y} | V=1 \right)= \sum_{d=0}^{1} Pr\left( D=d | V=1 \right)\prod_{j=1}^{J} Pr\left( Y_{j}=y_{j}| V=1,D=d ,\bigcap_{j^{'}=1|j>1}^{j-1} Y_{j^{'}=y_{j^{'}}} \right)$$

$$= \sum_{d=0}^{1} \theta_{d}\prod_{j=1}^{J} {p_{dj}}^{y_{j}}\left( 1-p_{dj} \right)^{1-y_{j}}\ldots\ldots\ldots\ldots\ldots\ldots\ldots\ldots\ldots\ldots\ldots.\ldots\ldots\ldots\left( 3 \right)$$

where

$$p_{dj}=Pr\left( Y_{j}=1| V=1,D=d, \bigcap_{j^{'}=1|j>1}^{j-1} Y_{j^{'}=y_{j^{'}}} \right)\text{and} \theta_{d}=Pr\left( D=d | V=1 \right), d\in\left\{ 0,1 \right\}$$

The probabilities $p_{dj}\text{and} \theta_{d}$can be calculated using regression methods.

Consequently,

$$Pr\left( Y_{j}=1 | V=1, D=d \right)=\sum_{Y_{j-1}\in\left( 0,1 \right)} \ldots\sum_{Y_{2}\in\left( 0,1 \right)} \sum_{Y_{1}\in\left( 0,1 \right)} \prod_{k=1}^{j} p_{dk}\ldots\ldots\ldots\ldots\ldots\ldots\ldots\ldots\left( 4 \right)$$

and $Pr\left( Y_{j}=0 | V=1, D=d \right)=1-Pr\left( Y_{j}=1 | V=1, D=d \right)$.

Unless the verified individuals represent a random sample of the population and are representative of the unverified individuals, inferences based on expressions (2) and (3) will suffer from verification bias. We put this in perspective by considering the community-based multi-morbidity survey. In this study, a subset of participants was eligible for microbiological testing using Xpert Ultra MTB/RIF^®^ (Xpert Ultra) and liquid culture if they reported any cardinal TB symptom (fever, night sweats, weight loss or cough) or if they had abnormal chest X-ray finding. Thus, asymptomatic individuals with normal chest X-ray findings have missing test results for Xpert Ultra and culture by design. Besides this, some individuals eligible for bacteriological testing had missing test results for Xpert Ultra and culture. Analysis that excludes unverified individuals may lead to incorrect inferences.

We derive a model that incorporates individuals not bacteriologically tested to mitigate potential verification bias. We begin with the simple model assuming the diagnostic tests are independent conditional on the true disease status. Noting that the probability of getting verified is dependent on the first *t* tests, expression (1) can be modified to allow plausible incorporation of the individuals who were not tested (or verified) using the remaining $J-t$ diagnostic tests as follows

$$Pr\left( \boldsymbol{Y} \right)=\sum_{d=0}^{1} Pr\left( D=d \right)\left\{ \left( \prod_{j=1}^{t} Pr\left( Y_{j}|D=d \right)Pr\left( V | Y_{1},Y_{2},\ldots,Y_{t},D \right) \right)\times\left( \prod_{j=t+1}^{J} \sum_{v=0}^{1} \frac{Pr\left( Y_{j}|D=d, V \right)Pr\left( D=d, V=v \right)}{Pr\left( D=d \right)} \right) \right\}\ldots\ldots\ldots\ldots\ldots\ldots\ldots\ldots\ldots\ldots\ldots\ldots\ldots.\ldots\left( 5 \right)$$

It is immediately clear that $Pr\left( Y_{j}|D=d, V=0 \right) , j=t+1, t+2, \ldots, J$ and $Pr\left( D=d | V=0 \right)$ cannot be estimated directly from the data.

We know can express $Pr\left( V=v | Y_{1},Y_{2},\ldots,Y_{t},D=d \right)$ as

$$\frac{Pr\left( Y_{1},Y_{2},\ldots,Y_{t} | V=v,D=d \right)Pr\left( V=v,D=d \right)}{Pr\left( Y_{1},Y_{2},\ldots,Y_{t} | D \right)Pr\left( D=d \right)}$$

Hence, expression (5) can simplify to

$$Pr\left( \boldsymbol{Y} \right)=\sum_{v=0}^{1} \sum_{d=0}^{1} Pr\left( V=v \right)Pr\left( D=d | V=v \right)\prod_{j=1}^{J} Pr\left( Y_{j}|D=d,V=v \right)\ldots\ldots\ldots\ldots\ldots\ldots\ldots\ldots. \left( 6 \right)$$

But expression (6) will lead to incorrect inferences if, conditional on the true disease status, all or a subset of the $J$ diagnostic tests are dependent.

We extend this concept to derive a model for dependent diagnostic tests that incorporates individuals not bacteriologically tested to help mitigate potential verification bias.

Let any TB symptom, radiologist conclusion, CAD4TBv7, Xpert Ultra and culture represent the sequence of the diagnostic tests. CAD4TBv7 became available later after the participants had been tested using Xpert Ultra and Culture. Nonetheless, we include it in the model so as to evaluate its diagnostic accuracy alongside the other diagnostic tests. Obviously, there might be some association between verification status and CAD4TBv7 because it was used to retrospectively interpret the same images interpreted by the radiologist. That, notwithstanding, the model can be expressed as follows

$$Pr\left( \boldsymbol{Y} \right)= \sum_{v=0}^{1} \sum_{d=0}^{1} Pr\left( V=v \right)Pr\left( D=d | V=v \right)\prod_{j=1}^{J} Pr\left( Y_{j}=y_{j}| V=v,D=d \bigcap_{j^{'}=1|j>1}^{j-1} Y_{j^{'}=y_{j^{'}}} \right)$$

$$= \sum_{v=0}^{1} \sum_{d=0}^{1} {\pi\theta}_{vd}\prod_{j=1}^{J} {p_{vdj}}^{y_{j}}\left( 1-p_{vdj} \right)^{1-y_{j}}\ldots\ldots\ldots\ldots\ldots\ldots\ldots\ldots\ldots\ldots\ldots.\ldots\ldots\ldots\left( 7 \right)$$

where

$p_{vdj}=Pr\left( Y_{j}=1| V=v,D=d \bigcap_{j^{'}=1|j>1}^{j-1} Y_{j^{'}=y_{j^{'}}} \right)\text{,} \theta_{vd}=Pr\left( D=d | V=v \right)$ and

$$\pi=Pr\left( V=v \right), v\in\left\{ 0,1 \right\}, d\in\left\{ 0,1 \right\}$$

The probabilities $p_{vdj}\text{and} \theta_{vd}$can also be calculated using regression methods.

Consequently,

$$Pr\left( Y_{j}=1 | D=d \right)=\sum_{Y_{j-1}\in\left( 0,1 \right)} \ldots\sum_{Y_{2}\in\left( 0,1 \right)} \sum_{Y_{1}\in\left( 0,1 \right)} \prod_{k=1}^{j} \left( {\pi p}_{1dk}+{\left( 1-\pi\right)p}_{0dk} \right)\ldots\ldots\ldots\ldots\ldots\ldots\ldots\left( 8 \right)$$

and $Pr\left( Y_{j}=0 | D=d \right)= 1-Pr\left( Y_{j}=1 | D=d \right)$.

Using the notation in the main text, i.e., $Y_{1},Y_{2},Y_{4},Y_{5}$ and $Y_{6}$ for any TB symptom, radiologist conclusion, CAD4TBv7, Xpert Ultra and culture respectively, notice that

$$p_{0d5}=Pr\left( Y_{5}=1| V=0,D=d \right)\text{, }p_{0d6}=Pr\left( Y_{6}=1| V=0,D=d, Y_{5}=y_{5} \right)\text{,}$$

$$\theta_{0d}=Pr\left( D=d | V=0 \right), d\in\left\{ 0,1 \right\}$$

cannot be calculated because Y_5_ and Y_6_ are not observed among the unverified (V=0) individuals. Under certain assumptions, we can impute these quantities. We assume that $p_{0dj}= {\emptyset\left( \epsilon_{jd} \right)p}_{1dj}, j\in\left\{ 5,6 \right\}, d\in\left\{ 0,1 \right\} \text{and} Pr\left( D=1 | V=0 \right)= \emptyset\left( \tau\right)Pr\left( D=1 | V=1 \right) \text{i.e} \theta_{01}=\emptyset\left( \tau\right)\theta_{11}$ where $0\leq\emptyset\left( \epsilon_{jd} \right) \leq1\text{ and 0≤ }\text{∅}\left( \tau\right)\text{ ≤1}$, Where $\emptyset\left( . \right)$ is the Gaussian cumulative distribution function.

The model can be extended to include measured covariates known to affect the diagnostic accuracy and/or prevalence as follows (noting that Y­­­_3_ is not included in the sequence)

$$Pr\left( \boldsymbol{Y} \right)= \sum_{v=0}^{1} \sum_{d=0}^{1} \sum_{x=0}^{1} Pr\left( X=x \right)Pr\left( V | X=x \right)Pr\left( D=d | V=v,X \right)\times\prod_{j=1}^{J} Pr\left( Y_{j}=y_{j}| X=x,V=v,D=d\bigcap_{j^{'}=1|j>1}^{j-1} Y_{j^{'}=y_{j^{'}}} \right)$$

For a vector $\boldsymbol{X}$ of p covariates,

$$Pr\left( \boldsymbol{X} \right)= Pr\left( X_{1},X_{2},\ldots,X_{p} \right)= Pr\left( X_{1} \right)Pr\left( X_{2} | X_{1} \right)\ldots Pr\left( X_{p} | X_{1},X_{2},\ldots X_{p-1} \right)$$

**Bayesian Inference**

Since the likelihood function of $\boldsymbol{y}_{\boldsymbol{i}}=\left( y_{i1},y_{i2},\cdots,y_{iJ} \right)$ depends on the (latent) disease status$d_{i}$ and the verification status $v_{i}$, $i=1,2,3,\cdots,N$ we have

$$Pr\left( \boldsymbol{y}_{\boldsymbol{i}},d_{i},v_{i}|\theta,p_{{vd}_{i}j} \right)=$$

$$\left[ \pi\left\{ \theta_{vd}\prod_{j=1}^{J} p_{{vd}_{i}j}^{y_{ij}}\left( 1-p_{{vd}_{i}j} \right)^{1-y_{ij}} \right\}^{d_{i}}\left\{ \left( 1-\theta_{vd} \right)\prod_{j=1}^{J} p_{{vd}_{i}j}^{y_{ij}}\left( 1-p_{{vd}_{i}j} \right)^{1-y_{ij}} \right\}^{1-d_{i}} \right]^{v_{i}}\times$$

$$\left[ \left( 1-\pi\right)\left\{ \theta_{vd}\prod_{j=1}^{J} p_{{vd}_{i}j}^{y_{ij}}\left( 1-p_{{vd}_{i}j} \right)^{1-y_{ij}} \right\}^{d_{i}}\left\{ \left( 1-\theta_{vd} \right)\prod_{j=1}^{J} p_{{vd}_{i}j}^{y_{ij}}\left( 1-p_{{vd}_{i}j} \right)^{1-y_{ij}} \right\}^{1-d_{i}} \right]^{{1-v}_{i}}..$$

$$\ldots\ldots\ldots\ldots\ldots\ldots\ldots\ldots\ldots\ldots\ldots\ldots\ldots\ldots\ldots\ldots\ldots\ldots\ldots\ldots\ldots\ldots\ldots\ldots\ldots\ldots\ldots\ldots\ldots\ldots\ldots\ldots\ldots\ldots\ldots\ldots\ldots\ldots\ldots\ldots\ldots\ldots\ldots\ldots\ldots\left( 9 \right)$$

We will only focus on the case where we have data on J diagnostic tests. Extension to incorporate measured covariates is straight forward. From (7) we have J conditional probability models to fit to determine $p_{vd_{i}j}, j=1,2,\ldots J$ among subjects whose verification status $V=v$. Since the outcome is Bernoulli distributed and $p_{{vd}_{i}j}$is related to a set of (binary) independent variables we can define a binary regression model as$p_{{ivd}_{i}j}=\emptyset\left( \boldsymbol{y}_{\boldsymbol{i}}^{\boldsymbol{T}}\boldsymbol{\beta}_{\boldsymbol{vdj}} \right)$, where $\boldsymbol{y}_{\boldsymbol{i}}^{\boldsymbol{T}}\boldsymbol{=}\left( y_{i0},y_{i1},\cdots,y_{ij} \right)$ is a $\left( j+1 \right)\times1$ vector of observed variables (or diagnostic tests) with $y_{i0}=1$, $\boldsymbol{\beta}_{\boldsymbol{vdj}}$ is a $\left( j+1 \right)\times1$ vector of unknown parameters to be estimated, and $\emptyset\left( . \right)$ is the Gaussian cumulative distribution function (CDF) linking the probabilities $p_{ivd_{i}j}$ with the linear component $\boldsymbol{y}_{\boldsymbol{i}}^{\boldsymbol{T}}\boldsymbol{\beta}_{\boldsymbol{vdj}}$ [3]. The unknown parameters $\boldsymbol{\beta}_{\boldsymbol{vdj}}$ were assigned $g\left( \boldsymbol{\beta}_{\boldsymbol{vdj}} \right)=N\left( \boldsymbol{\mu}_{\boldsymbol{vdj}}, \boldsymbol{\sigma}_{\boldsymbol{vdj}}^{\boldsymbol{2}}I_{j+1} \right)$priors, where $I_{j+1}$is an identity matrix of dimension$\left( j+1 \right)$ and $\boldsymbol{\sigma}_{\boldsymbol{vdj}}^{\boldsymbol{2}}$ is a $\left( j+1 \right)\times1$ vector of variances among the subjects whose true PTB status is$\boldsymbol{d}$. The variances may not necessarily be the same (S9 Table and S10 Table ). Similarly, the unobserved PTB status is a Bernoulli distributed latent variable. Therefore, the probability $\theta_{ivd}$ that the $i^{th}$ subject has PTB is related to a constant or a (set of) covariate(s) as follows: $\theta_{i11}=\emptyset\left( \boldsymbol{x}_{\boldsymbol{i}}^{\boldsymbol{T}}\boldsymbol{\omega} \right),$ where $\boldsymbol{x}_{\boldsymbol{i}}^{\boldsymbol{T}}\boldsymbol{=}\left( x_{i0},x_{i1},\cdots,x_{ip} \right)$ is a $\left( p+1 \right)\times1$ vector of observed covariates $(x_{i0}=1)$, $\boldsymbol{\omega}$ is a $\left( p+1 \right)\times1$ vector of unknown parameters to be estimated, and $\emptyset\left( . \right)$ is the Gaussian CDF linking the probabilities $\theta_{i11}$ with the linear component$\boldsymbol{x}_{\boldsymbol{i}}^{\boldsymbol{T}}\boldsymbol{\omega}$ . The unknown parameter(s) $\boldsymbol{\omega}$ were assigned $g\left( \boldsymbol{\omega} \right)=N\left( \boldsymbol{\mu}_{\boldsymbol{\theta}}, \boldsymbol{\sigma}_{\boldsymbol{\theta}}^{\boldsymbol{2}}\boldsymbol{I}_{p+1} \right)$ priors, where $I_{p+1}$is an identity matrix of dimension$p+1$ and $\boldsymbol{\sigma}_{\boldsymbol{\theta}}^{\boldsymbol{2}}$ is a $\left( p+1 \right)\times1$ vector of variances (for now we are working with $p+1=1$). The marginal probability of being verified was assigned a prior from Gaussian distribution. Thus, the posterior distribution $g\left( \boldsymbol{\omega}, \boldsymbol{\beta}_{\boldsymbol{vdj}}\boldsymbol{,\gamma|}\boldsymbol{y}_{\boldsymbol{i}},d \right)$ is proportional to

$$g\left( \boldsymbol{\gamma} \right)\prod_{i=1}^{N} \pi^{v_{i}}\left( 1-\pi\right)^{1-v_{i}}$$

$$\times g\left( \boldsymbol{\omega} \right)\prod_{i=1}^{N} \left\{ {\emptyset\left( \boldsymbol{x}_{\boldsymbol{i}}^{\boldsymbol{T}}\boldsymbol{\omega} \right)}^{d_{i}}\left( 1-\emptyset\left( \boldsymbol{x}_{\boldsymbol{i}}^{\boldsymbol{T}}\boldsymbol{\omega} \right) \right)^{1-d_{i}} \right\}^{v_{i}}\times g\left( \boldsymbol{\tau} \right)\prod_{i=1}^{N} \left\{ \left( \tau\emptyset\left( \boldsymbol{x}_{\boldsymbol{i}}^{\boldsymbol{T}}\boldsymbol{\omega} \right) \right)^{d_{i}}\left( 1-\tau\emptyset\left( \boldsymbol{x}_{\boldsymbol{i}}^{\boldsymbol{T}}\boldsymbol{\omega} \right) \right)^{1-d_{i}} \right\}^{{1-v}_{i}}\times\left[ \prod_{j=1}^{J} g\left( \boldsymbol{\beta}_{\mathbf{11}\boldsymbol{j}} \right)\prod_{i=1}^{N} \left\{ \left( \emptyset\left( \boldsymbol{y}_{\boldsymbol{i}}^{\boldsymbol{T}}\boldsymbol{\beta}_{\mathbf{11}\boldsymbol{j}} \right) \right)^{y_{ij}}\left( 1-\emptyset\left( \boldsymbol{y}_{\boldsymbol{i}}^{\boldsymbol{T}}\boldsymbol{\beta}_{\mathbf{11}\boldsymbol{j}} \right) \right)^{\left( {1-y}_{ij} \right)} \right\}^{d_{i}}\times\prod_{j=1}^{J} g\left( \boldsymbol{\beta}_{\mathbf{10}\boldsymbol{j}} \right)\prod_{i=1}^{N} \left\{ \left( \emptyset\left( \boldsymbol{y}_{\boldsymbol{i}}^{\boldsymbol{T}}\boldsymbol{\beta}_{\mathbf{10}\boldsymbol{j}} \right) \right)^{y_{ij}}\left( 1-\emptyset\left( \boldsymbol{y}_{\boldsymbol{i}}^{\boldsymbol{T}}\boldsymbol{\beta}_{\mathbf{10}\boldsymbol{j}} \right) \right)^{\left( {1-y}_{ij} \right)} \right\}^{{1-d}_{i}} \right]^{v_{i}}\times\left[ \prod_{j=1}^{J} g\left( \boldsymbol{\beta}_{\mathbf{01}\boldsymbol{j}} \right)\prod_{i=1}^{N} \left\{ \left( \emptyset\left( \boldsymbol{y}_{\boldsymbol{i}}^{\boldsymbol{T}}\boldsymbol{\beta}_{\mathbf{01}\boldsymbol{j}} \right) \right)^{y_{ij}}\left( 1-\emptyset\left( \boldsymbol{y}_{\boldsymbol{i}}^{\boldsymbol{T}}\boldsymbol{\beta}_{\mathbf{01}\boldsymbol{j}} \right) \right)^{\left( {1-y}_{ij} \right)} \right\}^{d_{i}}\times\prod_{j=1}^{J} g\left( \boldsymbol{\beta}_{\mathbf{00}\boldsymbol{j}} \right)\prod_{i=1}^{N} \left\{ \left( \emptyset\left( \boldsymbol{y}_{\boldsymbol{i}}^{\boldsymbol{T}}\boldsymbol{\beta}_{\mathbf{00}\boldsymbol{j}} \right) \right)^{y_{ij}}\left( 1-\emptyset\left( \boldsymbol{y}_{\boldsymbol{i}}^{\boldsymbol{T}}\boldsymbol{\beta}_{\mathbf{00}\boldsymbol{j}} \right) \right)^{\left( {1-y}_{ij} \right)} \right\}^{{1-d}_{i}} \right]^{{1-v}_{i}}\ldots..\ldots\left( 10 \right)$$

Where

$$\int g\left( \boldsymbol{\gamma} \right)\prod_{i=1}^{N} \pi^{v_{i}}\left( 1-\pi\right)^{1-v_{i}}d\pi$$

$$\int g\left( \boldsymbol{\beta}_{\boldsymbol{dj}} \right)\prod_{i=1}^{N} \left( \emptyset\left( \boldsymbol{y}_{\boldsymbol{i}}^{\boldsymbol{T}}\boldsymbol{\beta}_{\boldsymbol{dj}} \right) \right)^{y_{ij}}\left( 1-\emptyset\left( \boldsymbol{y}_{\boldsymbol{i}}^{\boldsymbol{T}}\boldsymbol{\beta}_{\boldsymbol{dj}} \right) \right)^{{1-y}_{ij}}d\boldsymbol{\beta}_{\boldsymbol{dj}}, d\epsilon\left\{ 0,1 \right\}$$

$$\int g\left( \boldsymbol{\omega} \right)\prod_{i=1}^{N} \left( \emptyset\left( \boldsymbol{x}_{\boldsymbol{i}}^{\boldsymbol{T}}\boldsymbol{\omega} \right) \right)^{y_{ij}}\left( 1-\emptyset\left( \boldsymbol{x}_{\boldsymbol{i}}^{\boldsymbol{T}}\boldsymbol{\omega} \right) \right)^{{1-y}_{ij}}d\boldsymbol{\omega}$$

are not easy to determine analytically. In this case the unknown parameters $\pi, \boldsymbol{\omega,}$ and $\boldsymbol{\beta}_{\boldsymbol{dj}}$,$j=1,2,\ldots,J$ will be estimated using Markov Chain Monte Carlo (MCMC) approach.

Note that we assumed $p_{0dj}= {\emptyset\left( \epsilon_{jd} \right)p}_{1dj}, j\in\left\{ 5,6 \right\}\text{, }d\in\left\{ 0,1 \right\} \text{and} Pr\left( D=1 | V=0 \right)= \emptyset\left( \tau\right)Pr\left( D=1 | V=1 \right) \text{i.e} \theta_{01}=\emptyset\left( \tau\right)\theta_{11}$ where $0\leq\emptyset\left( \epsilon_{jd} \right) \leq1\text{ and 0≤ }\text{∅}\left( \tau\right)\text{ ≤1}$.

Thus, we fit $p_{{ivd}_{i}j}=\emptyset\left( \boldsymbol{y}_{\boldsymbol{i}}^{\boldsymbol{T}}\boldsymbol{\beta}_{\boldsymbol{vdj}} \right), j=5,6$ and $\theta_{i11}= Pr\left( D=1 | V=1 \right)$ among the subjects who were verified and use the model to impute the conditional probabilities for subjects who were not verified. The estimates of $p_{{i0d}_{i}j}$ and $\theta_{i01}$among the unverified subjects are assumed to be $\emptyset\left( \epsilon_{jd} \right)\emptyset\left( \boldsymbol{y}_{\boldsymbol{i}}^{\boldsymbol{T}}\boldsymbol{\beta}_{\boldsymbol{vdj}} \right)$ and $\emptyset\left( \tau\right)\emptyset\left( \boldsymbol{x}_{\boldsymbol{i}}^{\boldsymbol{T}}\boldsymbol{\omega} \right)$ respectively, where the parameters $\epsilon_{jd}$and $\text{τ}$ are assigned priors from Gaussian distribution. For the purpose of this analysis, the unverified group was split into two: eligible but unverified and ineligible and unverified cases.

**Missing Data Mechanism and imputation**

We handled imputation of missing data and posterior inferences simultaneously in one MCMC algorithm.[4] The unknown Xpert Ultra and culture status were imputed within the latent class model under the assumption that Xpert Ultra and culture test results among the individuals who were not bacteriologically tested (or verified) were: (1) missing at random (MAR), and (2) missing not at random (MNAR).[5] Under the MAR assumption, we assumed that only the earlier diagnostic tests and the measured covariates determined bacteriological testing using Xpert Ultra and culture. Cognizant of the dependencies between the diagnostic tests as explained and depicted in Fig 1 in the main manuscript, we fitted a probit regression model of the probability of Xpert Ultra positive for the individuals who were microbiologically tested and truly have PTB. We then used the model to impute the missing Xpert Ultra test results among the true have PTB. We also fitted a probit regression model of the probability of culture positive conditional on Xpert Ultra among the individuals who were microbiologically tested and truly have PTB. We then used the model to impute the culture test results among the individuals who truly have PTB. Similarly, we fitted a probit regression model for Xpert Ultra positive and another for culture positive among the true non-PTB cases and used them to impute the missing Xpert Ultra and culture test results among the true non-PTB cases. In a separate analysis we fitted the same models adjusted for the measured covariates. In our model presented above, $\emptyset\left( \epsilon_{jd} \right)=1$ or simply $\epsilon_{jd}=0$ for $j\in\left\{ 5,6 \right\}$ under the MAR assumption. Under the MNAR assumption, we assume that other than the earlier diagnostic tests and the measured covariates, there are some unmeasured variables that additionally determine microbiological testing e.g., unmeasured bacterial load among other possible unmeasured variables represented by W in Fig 1 of the main manuscript. Under this assumption, we fitted similar regressive probit models as done under MAR assumption but allowed the unmeasured factor(s) to scale the conditional probability of Xpert Ultra and culture positive among the individuals who were not microbiologically tested as defined in our model above. The effect of the unmeasured variables was defined as $0\leq\emptyset\left( \epsilon_{jd} \right) \leq1$. This was based on the argument that the microbiologically untested individuals have a low marginal probability of TB by design i.e., based on the eligibility criteria for microbiological testing. Therefore, the conditional probability of a positive Xpert Ultra and culture among the microbiologically untested individuals are bound to be lower or equal to the conditional probability of a positive Xpert Ultra and culture among the microbiologically tested individuals.

The third option for imputing the unknown Xpert Ultra and culture test results was the multivariate imputation via chained equations (MICE).[6] This approach assumes the missing data are MAR. We imputed 100 datasets after allowing 50 iterations for each variable during imputation. The prediction model for Xpert Ultra comprised of any TB symptom, chest X-ray abnormality suggestive of active TB, CAD4TBv5 (continuous), CAD4TBv6 (continuous), culture, age (categorical), sex and HIV status as covariates in the model. Similarly, the prediction model for culture comprised of any TB symptom, chest X-ray abnormality suggestive of active TB, CAD4TBv5 (continuous), CAD4TBv6 (continuous), Xpert Ultra, age (categorical), sex and HIV status as covariates in the model. Xpert Ultra, culture and HIV status were imputed using logistic regression model while age was imputed using polytomous regression model treating the outcome as an ordinal variable. Any chest X-ray abnormality was not used as a covariate in the model in order to minimize incorrect prediction as a result of abnormality that is not suggestive of active TB. Essentially, we adopted the approach usually used in prevalence surveys to ascertain the correct Xpert Ultra status that usually includes chest X-ray abnormality suggestive of active TB in the algorithm.[7] Individuals with missing age and HIV status were imputed as well but were excluded from the analysis to ensure comparison of the models with the same number of cases. The columns of the matrix below (S8 Table) show the variables included as covariates in the imputation models while the rows depict the variables being imputed (outcomes). A value of 1 indicates the variables was included as a predictor. A value of zero indicates that the variables was not included as a predictor in the model. Though included in the rows of the predictor matrix, any TB symptom, chest X-ray abnormality suggestive of active TB, CAD4TBv5 (continuous), CAD4TBv6 (continuous), and sex were not imputed.

**S8 Table: Predictor matrix showing the variables used to predict the missing data**

|  | **Y1** | **Y2** | **Y4** | **Y5** | **Y6** | **X1** | **X2** | **X3** | **X4** | **X5** |
| --- | --- | --- | --- | --- | --- | --- | --- | --- | --- | --- |
| **Y1** | 0 | 1 | 0 | 1 | 1 | 1 | 1 | 1 | 1 | 1 |
| **Y2** | 1 | 0 | 0 | 1 | 1 | 1 | 1 | 1 | 1 | 1 |
| **Y4** | 1 | 1 | 0 | 1 | 1 | 1 | 1 | 1 | 1 | 1 |
| **Y5** | 1 | 1 | 0 | 0 | 1 | 1 | 1 | 1 | 1 | 1 |
| **Y6** | 1 | 1 | 0 | 1 | 0 | 1 | 1 | 1 | 1 | 1 |
| **X1** | 1 | 1 | 0 | 1 | 1 | 0 | 1 | 1 | 1 | 1 |
| **X2** | 1 | 1 | 0 | 1 | 1 | 1 | 0 | 1 | 1 | 1 |
| **X3** | 1 | 1 | 0 | 1 | 1 | 1 | 1 | 0 | 1 | 1 |
| **X4** | 1 | 1 | 0 | 1 | 1 | 1 | 1 | 1 | 0 | 1 |
| **X5** | 1 | 1 | 0 | 1 | 1 | 1 | 1 | 1 | 1 | 0 |

$Y_{1}$ – Any TB symptom, $Y_{2}$ – Chest X-ray abnormality suggestive of active TB, $Y_{4}$ – CAD4TBv7 (binary) $Y_{5}$ –Xpert Ultra, $Y_{6}$ – Culture, $X_{1}$ – Age (categorized), $X_{2}$ – Sex, $X_{3}$ – HIV status, $X_{4}-$CAD4TBv5 (continuous), $X_{5}-$CAD4TBv6 (continuous)

CAD4TBv6 and CAD4TBv7 became available later and were used to retrospectively analyze the digital chest X-ray images. Hence, we avoided using CAD4TBv7 to impute the missing bacteriological test results. Nonetheless, because we were not evaluating CAD4TBv6, we included it to predict the missing Xpert Ultra and culture. Note that any TB symptom, radiologist conclusion and CAD4TBv5 were included because they determined bacteriological testing. We generated trace plots of the imputed variables to help assess the quality of the imputed data. The trace plots below reveal good convergence of the imputation models for Xpert Ultra, culture and HIV status. The trace plot for age is not great with a variance of zero because there was only one individual who was missing. Hence it is not shown.


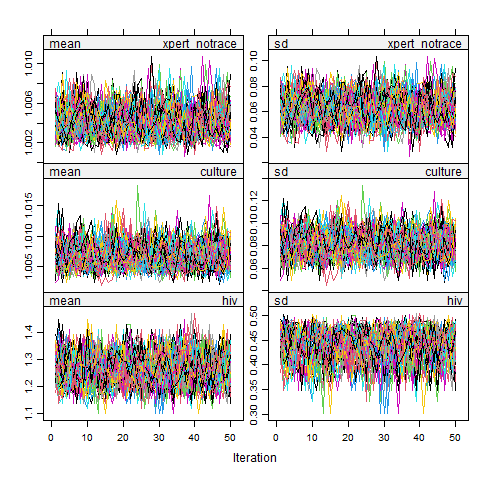


**S3 Fig: Trace plots depicting convergence in the imputation models among the microbiologically untested individuals**

**Prior distributions**

S9 Table and S10 Table present the prior distributions for the parameters of the probit regression models. Column 1 (with V = 1) presents the priors for the complete-case analysis, analysis assuming bacteriologically unverified individuals are negative for Xpert Ultra and culture, and multiply-imputed data (Vukuzazi data only). The model that simultaneously imputes the missing Xpert Ultra and culture test results among the individuals who were not bacteriologically tested under the MAR and MNAR assumptions used the priors presented in the columns with V = 1 and V = 0. The unverified participants were split into two groups: eligible but unverified (G=2) and the ineligible and unverified (G=3). These two groups use similar priors specified in the column with V = 0. The verified group is denoted G=1.

**S9 Table: Prior distributions for the parameters of the probit regression models for complete-case analysis, assuming the bacteriologically unverified individuals are negative for Xpert Ultra and culture, multiply-imputed data and simultaneous imputation of missing Xpert Ultra and culture test results under MAR and MNAR assumptions as used in the analysis of Vukuzazi data (not adjusted for measured covariates) and simulated data**

|  | Model structure | V = 1 or G=1 | V = 0 or G=2 & 3 |
| --- | --- | --- | --- |
| D | $Pr\left( D=1\vert V=1 \right)=\emptyset\left( \omega_{0} \right)$ | $g\left( \omega_{0} \right)\sim\text{N(-3,0.1)}$ |  |
|  | $Pr\left( D=1\vert V=0 \right)=\emptyset\left( \tau\right) Pr\left( D=1\vert V=1 \right)$ |  | $g\left( \tau\right)\sim N\left( 0,10 \right)$ |
| $Y_{1}$ | $Pr\left( Y_{i1}=1 \vert D_{i}=1\text{, V=v} \right)=\emptyset\left( \beta_{11} \right)$ | $g\left( \beta_{11} \right)\sim\text{N(0,1)}$ | $g\left( \beta_{11} \right)\sim\text{N(0,1)}$ |
|  | $Pr\left( Y_{i1}=1 \vert D_{i}=0\text{, V=v} \right)=\emptyset\left( \alpha_{11} \right)$ | $g\left( \alpha_{11} \right)\sim\text{N(0,1)}$ | $g\left( \alpha_{11} \right)\sim\text{N(0,1)}$ |
| $Y_{2}$ | $Pr\left( Y_{i2}=1 \vert D_{i}\text{=1, V=v} \right)=\emptyset\left( \beta_{12} \right)$ | $g\left( \beta_{12} \right)\sim\text{N(0,1)}$ | $g\left( \beta_{12} \right)\sim\text{N(0,}1\text{)}$ |
|  | $Pr\left( Y_{i2}=1 \vert{Y_{i1},D}_{i}\text{=0, V=v} \right)=\emptyset\left( \alpha_{12}\text{+ }\alpha_{22}y_{i1} \right)$ | $g\left( \alpha_{12} \right)\sim\text{N(0,1)}$  $g\left( \alpha_{22} \right)\sim\text{N(0,1)}$ | $g\left( \alpha_{12} \right)\sim\text{N(0,1)}$  $g\left( \alpha_{22} \right)\sim\text{N(0,1)}$ |
| $Y_{4}$ | $Pr\left( Y_{i4}=1 \vert Y_{i2},D_{i}\text{=1, V=v} \right)= \emptyset\left( \beta_{14}\text{+ }\beta_{24}y_{i2} \right)$ | $g\left( \beta_{14} \right)\sim\text{N(0,1)}$  $g\left( \beta_{24} \right)\sim\text{N(0,1)}$ | $g\left( \beta_{14} \right)\sim\text{N(0,1)}$  $g\left( \beta_{24} \right)\sim\text{N(0,1)}$ |
|  | $Pr(Y_{i4}=1\vert{Y_{i1},Y_{i2},D}_{i}\text{=0, V=v})=\emptyset\left( \alpha_{14}\text{+ }\alpha_{24}y_{i1}\text{+ }\alpha_{34}y_{i2} \right)$ | $g\left( \alpha_{14} \right)\sim\text{N(0,1)}$  $g\left( \alpha_{24} \right)\sim\text{N(0,1)}$  $g\left( \alpha_{34} \right)\sim\text{N(0,1)}$ | $g\left( \alpha_{14} \right)\sim\text{N(0,1)}$  $g\left( \alpha_{24} \right)\sim\text{N(0,1)}$  $g\left( \alpha_{34} \right)\sim\text{N(0,1)}$ |
| $Y_{5}$ | $Pr\left( Y_{i5}=1 \vert D_{i}\text{=1, V=1} \right)=\emptyset\left( \beta_{15} \right)$ | $g\left( \beta_{15} \right)\sim\text{N(0,1)}$ |  |
|  | $Pr\left( Y_{i5}=1 \vert D_{i}\text{=1, V=0} \right)$  $=\emptyset\left( \epsilon_{51} \right)Pr\left( Y_{i5}=1 \vert D_{i}\text{=1, V=1} \right)$ |  | $g\left( \epsilon_{51} \right)\sim N\left( 0,1 \right)$ |
|  | $Pr(Y_{i5}=1\vert D_{i}\text{=0, V=1})=\emptyset\left( \alpha_{15} \right)$ | $g\left( \alpha_{15} \right)\sim\text{N(-3,0.1)}$ |  |
|  | $\Pr\left( Y_{i5}=1 \vert D_{i}\text{=0, V=0} \right)=$  $\emptyset\left( \epsilon_{50} \right)Pr(Y_{i5}=1\vert D_{i}\text{=0, V=1})$ |  | $g\left( \epsilon_{50} \right)\sim N\left( 0,1 \right)$ |
| $Y_{6}$ | $Pr\left( Y_{i6}=1 \vert Y_{i5},D_{i}\text{=1, V=1} \right)=\emptyset\left( \beta_{16}\text{+ }\beta_{26}y_{i5} \right)$ | $g\left( \beta_{16} \right)\sim\text{N(0,1)}$  $g\left( \beta_{26} \right)\sim\text{N(0,1)}$ |  |
|  | $Pr\left( Y_{i6}=1 \vert Y_{i5},D_{i}\text{=1, V=0} \right)$  $=\emptyset\left( \epsilon_{61} \right)Pr\left( Y_{i6}=1 \vert Y_{i5},D_{i}\text{=1, V=1} \right)$ |  | $g\left( \epsilon_{61} \right)\sim N\left( 0,1 \right)$ |
|  | $Pr(Y_{i6}=1\vert D_{i}\text{=0, V=1})=\emptyset\left( \alpha_{16} \right)$ | $g\left( \alpha_{16} \right)\sim\text{N(-3,0.1)}$ |  |
|  | $\Pr\left( Y_{i6}=1 \vert D_{i}\text{=0, V=0} \right)$  $=\emptyset\left( \epsilon_{60} \right)Pr(Y_{i6}=1\vert D_{i}\text{=0, V=1})$ |  | $g\left( \epsilon_{60} \right)\sim N\left( 0,1 \right)$ |

D – Disease (PTB), $Y_{1}$ – any TB symptom, $Y_{2}$ – Radiologist conclusion, $Y_{4}$ – CAD4TBv7 ≥18.28, $Y_{5}$ – Xpert Ultra, $Y_{6}$ – Culture. Complete-case analysis, analysis assuming bacteriologically unverified individuals are negative for Xpert Ultra and culture, and multiply-imputed data. The model that simultaneously imputes the missing Xpert Ultra and culture test results among the individuals who were not bacteriologically tested under MAR assumption used the priors presented in the columns with V=1 but models $Pr\left( D=1|V=1 \right)=\emptyset\left( \omega_{0}+\omega_{1}EU{+\omega}_{2}IU \right)$, EU – Eligible unverified, IU – Ineligible unverified, with $g\left( \omega_{0} \right)\sim\text{N(-3,0.1)}$*,* $g\left( \omega_{1} \right)\sim\text{N(0,0.1)}$*,* $g\left( \omega_{2} \right)\sim\text{N(-3,0.1) }$and assumes $g\left( \tau\right)=g\left( \epsilon_{51} \right)$=$g\left( \epsilon_{50} \right)$ = $g\left( \epsilon_{61} \right)$ = $g\left( \epsilon_{60} \right)$=1, and under MNAR the model uses $g\left( \tau\right)=N\left( 0,10 \right)$ for the EU and $g\left( \tau\right)=N\left( -1,10 \right)$ for the IU and uses priors in the columns with V=1 and V=0.

**S10 Table: Prior distributions for the model with simultaneous imputation of missing Xpert Ultra and culture test results under MAR and MNAR assumptions as used in the analysis of Vukuzazi data, adjusted for measured covariates**

|  | Model structure | V = 1 or G=1 | V = 0 or G=2 & 3 |
| --- | --- | --- | --- |
| D | $Pr\left( D=1 \vert X_{i1},X_{i2},X_{i3}, V=1 \right)$= $\emptyset\left( \begin{aligned} \omega_{0}+\omega_{1}x_{i3}+\omega_{2}x_{i2}+\omega_{3}I\left( x_{i1}=2 \right)+ \\ \omega_{4}I\left( x_{i1}=3 \right)+\omega_{5}I\left( x_{i1}=4 \right) \end{aligned} \right)$ | $g\left( \omega_{0} \right)\sim\text{N(-3,0.1)}$  $g\left( \omega_{1} \right)\sim\text{N(0,0.1),}$…,$g\left( \omega_{5} \right)\sim\text{N(}\text{0,0.1)}$ |  |
|  | $Pr\left( D=1 \vert X_{i1},X_{i2},X_{i3}, V=0 \right)$  $= \emptyset\left( \tau\right)Pr\left( D=1 \vert X_{i1},X_{i2},X_{i3}, V=1 \right)$ |  | $g\left( \tau\right)\sim\text{N(0,0.1)}$ |
| $Y_{1}$ | $Pr\left( Y_{i1}=1 \vert{X_{i1},X_{i2},X_{i3},D}_{i}=1, V=v \right)=\emptyset\left( \begin{aligned} \beta_{11}+\beta_{21}x_{i3}+\beta_{31}x_{i2}+\beta_{41}I\left( x_{i1}=2 \right)+ \\ \beta_{51}I\left( x_{i1}=3 \right)+\beta_{61}I\left( x_{i1}=4 \right) \end{aligned} \right)$ | $g\left( \beta_{11} \right)\sim\text{N(-1,0.2)}$  $g\left( \beta_{21} \right),\ldots,g\left( \beta_{61} \right)\sim\text{N(0,0.2)}$ | $g\left( \beta_{11} \right)\sim\text{N(-1,0.2)}$  $g\left( \beta_{21} \right),\ldots,g\left( \beta_{61} \right)\sim\text{N(0,0.2)}$ |
|  | $Pr\left( Y_{i1}=1 \vert X_{i1},X_{i2},X_{i3},D_{i}=0, V=v \right)$  $=\emptyset\left( \begin{aligned} \alpha_{11}\text{+ }\alpha_{21}x_{i3}+\alpha_{31}x_{i2}+\alpha_{41}I\left( x_{i1}=2 \right) \\ +\alpha_{51}I\left( x_{i1}=3 \right)+ \alpha_{61}I\left( x_{i1}=4 \right) \end{aligned} \right)$ | $g\left( \alpha_{11} \right)\sim\text{N(0,1)}$  $g\left( \alpha_{21} \right),\ldots,g\left( \alpha_{61} \right)\sim\text{N(0,}1\text{)}$ | $g\left( \alpha_{11} \right)\sim\text{N(0,1)}$  $g\left( \alpha_{21} \right),\ldots,g\left( \alpha_{61} \right)\sim\text{N(0,}1\text{)}$ |
| $Y_{2}$ | $Pr\left( Y_{i2}=1 \vert{X_{i1},X_{i2},X_{i3},D}_{i}\text{=1}, V=v \right)=$  $\emptyset\left( \begin{aligned} \beta_{12}+\beta_{22}x_{i3}+\beta_{32}x_{i2}+\beta_{42}I\left( x_{i1}=2 \right)+ \\ \beta_{52}I\left( x_{i1}=3 \right)+\beta_{62}I\left( x_{i1}=4 \right) \end{aligned} \right)$ | $g\left( \beta_{12} \right)\sim\text{N(1,0.2)}$  $g\left( \beta_{22} \right),\ldots,g\left( \beta_{62} \right)\sim\text{N(0,0.2)}$ | $g\left( \beta_{12} \right)\sim\text{N(1,0.2)}$  $g\left( \beta_{22} \right),\ldots,g\left( \beta_{62} \right)\sim\text{N(0,0.2)}$ |
|  | $Pr\left( Y_{i2}=1 \vert{Y_{i1},X_{i1},X_{i2},X_{i3},D}_{i}\text{=0}, V=v \right)=$  $\emptyset\left( \begin{aligned} \alpha_{12}\text{+ }\alpha_{22}y_{i1}+\alpha_{32}x_{i3}+\alpha_{42}x_{i2}+\alpha_{52}I\left( x_{i1}=2 \right) \\ +\alpha_{62}I\left( x_{i1}=3 \right)+ \alpha_{72}I\left( x_{i1}=4 \right) \end{aligned} \right)$ | $g\left( \alpha_{12} \right),g\left( \alpha_{22} \right)\sim\text{N(0,1)}$  $g\left( \alpha_{32} \right),\ldots,g\left( \alpha_{72} \right)\sim\text{N(0,}1\text{)}$ | $g\left( \alpha_{12} \right),g\left( \alpha_{22} \right)\sim\text{N(0,1)}$  $g\left( \alpha_{32} \right),\ldots,g\left( \alpha_{72} \right)\sim\text{N(0,}1\text{)}$ |
| $Y_{4}$ | $Pr\left( Y_{i4}=1 \vert Y_{i2},{X_{i1},X_{i2},X_{i3},D}_{i}\text{=1}, V=v \right)=$  $\emptyset\left( \begin{aligned} \beta_{14}\text{+ }\beta_{24}y_{i2}+\beta_{34}x_{i3}+\beta_{44}x_{i2}+\beta_{54}I\left( x_{i1}=2 \right) \\ +\beta_{63}I\left( x_{i1}=3 \right)+\beta_{73}I\left( x_{i1}=4 \right) \end{aligned} \right)$ | $g\left( \beta_{14} \right)\sim\text{N(1,0.2)}$  $g\left( \beta_{24} \right),\ldots,g\left( \beta_{74} \right)\sim\text{N(0,0.2)}$ | $g\left( \beta_{14} \right)\sim\text{N(1,0.2)}$  $g\left( \beta_{24} \right),\ldots,g\left( \beta_{74} \right)\sim\text{N(0,0.2)}$ |
|  | $\Pr\left( Y_{i4}=1 \vert{Y_{i1},Y_{i2},X_{i1},X_{i2},X_{i3},D}_{i}\text{=0}, V=v \right)=$  $\emptyset\left( \begin{aligned} \alpha_{14}\text{+ }\alpha_{24}y_{i1}\text{+ }\alpha_{34}y_{i2}+\alpha_{44}x_{i3}+\alpha_{54}x_{i2} \\ +\alpha_{64}I\left( x_{i1}=2 \right)+\alpha_{74}I\left( x_{i1}=3 \right){+\alpha}_{84}I\left( x_{i1}=4 \right) \end{aligned} \right)$ | $g\left( \alpha_{14} \right),\ldots,g\left( \alpha_{34} \right)\sim\text{N(0,1)}$  $g\left( \alpha_{44} \right),\ldots,g\left( \alpha_{84} \right)\sim\text{N(0,}1\text{)}$ | $g\left( \alpha_{14} \right),\ldots,g\left( \alpha_{34} \right)\sim\text{N(0,1)}$  $g\left( \alpha_{44} \right),\ldots,g\left( \alpha_{84} \right)\sim\text{N(0,}1\text{)}$ |
| $Y_{5}$ | $Pr\left( Y_{i5}=1 \vert X_{i1},X_{i2},X_{i3},D_{i}\text{=1}, V=1 \right)=$  $\emptyset\left( \begin{aligned} \beta_{15}\text{+ }\beta_{25}x_{i3}+\beta_{35}x_{i2}+\beta_{45}I\left( x_{i1}=2 \right)+\beta_{55} \\ I\left( x_{i1}=3 \right)+\beta_{65}I\left( x_{i1}=4 \right) \end{aligned} \right)$ | $g\left( \beta_{15} \right)\sim\text{N(0.6,0.2)}$  $g\left( \beta_{25} \right),\ldots,g\left( \beta_{65} \right)\sim\text{N(0,0.2)}$ |  |
|  | $Pr\left( Y_{i5}=1 \vert X_{i1},X_{i2},X_{i3},V=0,D_{i}\text{=1} \right)$  $=\emptyset\left( \epsilon_{51} \right)Pr\left( Y_{i5}=1 \vert X_{i1},X_{i2},X_{i3},V=1,D_{i}\text{=1} \right)$ |  | $g\left( \epsilon_{51} \right)\sim N\left( 0,1 \right)$ |
|  | $Pr\left( Y_{i5}=1 \vert D_{i}\text{=0}, V=1 \right)=\emptyset\left( \alpha_{15} \right)$ | $g\left( \alpha_{15} \right)\sim\text{N(-3,0.1)}$ |  |
|  | $Pr\left( Y_{i5}=1 \vert{V=0,D}_{i}\text{=0} \right)=\emptyset\left( \epsilon_{50} \right)Pr\left( Y_{i5}=1 \vert{V=1,D}_{i}\text{=0} \right)$ |  | $g\left( \epsilon_{50} \right)\sim N\left( 0,1 \right)$ |
| $Y_{6}$ | $Pr\left( Y_{i6}=1 \vert Y_{i5},{X_{i1},X_{i2},X_{i3},D}_{i}\text{=1}, V=1 \right)=$  $\emptyset\left( \begin{aligned} \beta_{16}\text{+ }\beta_{26}y_{i5}+\beta_{36}x_{i3}+\beta_{46}x_{i2}+\beta_{56}I\left( x_{i1}=2 \right)+ \\ \beta_{66}I\left( x_{i1}=3 \right)+\beta_{76}I\left( x_{i1}=4 \right) \end{aligned} \right)$ | $g\left( \beta_{16} \right)\sim\text{N(0.6,0.2)}$  $g\left( \beta_{26} \right),\ldots,g\left( \beta_{76} \right)\sim\text{N(0,0.2)}$ |  |
|  | $Pr\left( Y_{i6}=1 \vert Y_{i5},{X_{i1},X_{i2},X_{i3}, V=0,D}_{i}\text{=1} \right)$  $=\emptyset\left( \epsilon_{61} \right)Pr\left( Y_{i6}=1 \vert Y_{i5},{X_{i1},X_{i2},X_{i3}, V=1,D}_{i}\text{=1} \right)$ |  | $g\left( \epsilon_{61} \right)\sim N\left( 0,1 \right)$ |
|  | $Pr\left( Y_{i6}=1 \vert D_{i}\text{=0}, V=1 \right)=\emptyset\left( \alpha_{16} \right)$ | $g\left( \alpha_{16} \right)\sim\text{N(-3,0.1)}$ |  |
|  | $Pr\left( Y_{i6}=1 \vert{V=0,D}_{i}\text{=0} \right)=\emptyset\left( \epsilon_{60} \right)Pr\left( Y_{i6}=1 \vert{V=1,D}_{i}\text{=0} \right)$ |  | $g\left( \epsilon_{60} \right)\sim N\left( 0,1 \right)$ |

D – Disease (PTB), $Y_{1}$ – any TB symptom, $Y_{2}$ – Radiologist conclusion, $Y_{4}$ – CAD4TBv7 ≥18.28, $Y_{5}$ – Xpert Ultra, $Y_{6}$ – Culture. Assuming the missing data is MAR the model used the priors presented in the columns with V=1 but models $Pr\left( D=1|X,V=1 \right)=\emptyset\left( \omega_{0}+\omega_{1}EU{+\omega}_{2}IU+\omega_{x}X \right)$, EU – Eligible unverified, IU – Ineligible unverified, with $g\left( \omega_{0} \right)\sim\text{N(-3,0.1)}$*,* $g\left( \omega_{1} \right)\sim\text{N(0,0.1)}$*,* $g\left( \omega_{2} \right)\sim\text{N(-1,0.1) }$*,* $g\left( \omega_{x} \right)\sim\text{N(0,0.1), }\omega_{x}=5\times1$ vector and $g\left( \tau\right)=g\left( \epsilon_{51} \right)$=$g\left( \epsilon_{50} \right)$ = $g\left( \epsilon_{61} \right)$ = $g\left( \epsilon_{60} \right)$=1, and assuming the missing data is MNAR the model uses $g\left( \tau\right)=N\left( 0,10 \right)$ for the EU and $g\left( \tau\right)=N\left( -1,10 \right)$ for the IU and uses the priors in the columns with V=1 and V=0 for all the other parameters.

Next, we present the model results for the analysis of the simulated data and analysis of Vukuzazi data. We present the model estimates alongside the statistics used to assess convergence of the parameters under evaluation. First, we present the model results for the simulated data. For this, we present the results of a randomly chosen dataset from the set of 100 replicate datasets. As reported in the methods section of the main document, the results of the 100 replicate datasets were combined to obtain the results reported in the Table 2 of the main document.

1. Analysis of simulated data (Table 2 in the main document)
2. Model results for the simulated data based on the composite reference standard.
   - 1. Complete-case analysis (Results of the 58^th^ replicate dataset randomly chosen from among the model results of the 100 replicate datasets)

Inference for Bugs model at "model.naive", fit using jags,

3 chains, each with 50000 iterations (first 25000 discarded), n.thin = 10 n.sims = 7500 iterations saved

mu.vect sd.vect 50% 2.5% 97.5% Rhat n.eff

pi 0.047 0.003 0.047 0.041 0.054 1.002 2000

se[1] 0.167 0.025 0.166 0.120 0.217 1.001 7500

se[2] 0.577 0.033 0.578 0.514 0.641 1.001 7500

se[3] 0.746 0.029 0.747 0.687 0.801 1.001 7500

se[4] 0.640 0.032 0.641 0.576 0.702 1.001 7500

se[5] 0.787 0.027 0.788 0.732 0.838 1.001 4200

sp[1] 0.780 0.006 0.780 0.767 0.792 1.001 7500

sp[2] 0.609 0.007 0.609 0.595 0.623 1.001 7500

sp[3] 0.515 0.007 0.515 0.501 0.530 1.001 7500

sp[4] 1.000 0.000 1.000 0.999 1.000 1.001 7500

sp[5] 1.000 0.000 1.000 0.999 1.000 1.001 6900

deviance 4590.548 5.912 4589.802 4581.042 4603.971 1.001 4700

For each parameter, n.eff is a crude measure of effective sample size, and Rhat is the potential scale reduction factor (at convergence, Rhat=1).

DIC info (using the rule, pD = var(deviance)/2)

pD = 17.5 and DIC = 4608.0

DIC is an estimate of expected predictive error (lower deviance is better).

- - 1. Analysis assuming the participants with missing values in Y5 and Y6 were negative for Y5 and Y6 (Results of the 88th replicate dataset randomly chosen from among the model results of the 100 replicate datasets)

Inference for Bugs model at "model.naive", fit using jags,

3 chains, each with 50000 iterations (first 25000 discarded), n.thin = 10 n.sims = 7500 iterations saved

mu.vect sd.vect 50% 2.5% 97.5% Rhat n.eff

pi 0.020 0.001 0.020 0.017 0.023 1.001 7500

se[1] 0.268 0.032 0.267 0.208 0.332 1.001 6200

se[2] 0.585 0.035 0.585 0.516 0.652 1.001 5300

se[3] 0.752 0.031 0.753 0.690 0.810 1.001 7300

se[4] 0.683 0.033 0.683 0.616 0.746 1.001 7500

se[5] 0.753 0.031 0.754 0.689 0.809 1.001 5100

sp[1] 0.878 0.003 0.878 0.871 0.884 1.001 7500

sp[2] 0.776 0.004 0.776 0.768 0.784 1.002 2000

sp[3] 0.699 0.005 0.699 0.690 0.708 1.001 7500

sp[4] 1.000 0.000 1.000 0.999 1.000 1.001 7500

sp[5] 1.000 0.000 1.000 0.999 1.000 1.001 5400

deviance 8467.611 5.998 8467.020 8457.816 8481.066 1.001 6600

For each parameter, n.eff is a crude measure of effective sample size, and Rhat is the potential scale reduction factor (at convergence, Rhat=1).

DIC info (using the rule, pD = var(deviance)/2)

pD = 18.0 and DIC = 8485.6

DIC is an estimate of expected predictive error (lower deviance is better).

1. Model results for the simulated data based on Bayesian LCA
   1. Complete case analysis (Results based on the analysis of the 23^rd^ replicate dataset randomly chosen from among the model results of the 100 replicate datasets)

Inference for Bugs model at "sim_model1_CC.txt", fit using jags,

3 chains, each with 50000 iterations (first 25000 discarded), n.thin = 10 n.sims = 7500 iterations saved

mu.vect sd.vect 50% 2.5% 97.5% Rhat n.eff

a_prev -1.842 0.071 -1.851 -1.956 -1.670 1.002 7500

alpha[1,1] -0.787 0.021 -0.787 -0.828 -0.747 1.001 7500

alpha[1,2] -0.522 0.023 -0.522 -0.567 -0.478 1.001 3600

alpha[2,2] 0.797 0.047 0.797 0.707 0.890 1.001 3000

alpha[1,3] -1.322 0.036 -1.322 -1.394 -1.252 1.001 7500

alpha[2,3] 1.646 0.074 1.645 1.500 1.791 1.001 7500

alpha[3,3] 3.185 0.086 3.184 3.023 3.356 1.001 4100

alpha[1,4] -2.531 0.080 -2.526 -2.704 -2.389 1.001 7500

alpha[1,5] -2.629 0.161 -2.606 -3.005 -2.383 1.001 7500

beta[1,1] -0.778 0.123 -0.778 -1.024 -0.534 1.001 7500

beta[1,2] 0.530 0.120 0.530 0.300 0.771 1.001 7500

beta[1,3] 0.354 0.221 0.350 -0.077 0.792 1.001 7500

beta[2,3] 1.391 0.347 1.387 0.731 2.104 1.001 5000

beta[1,4] 0.384 0.224 0.401 -0.107 0.784 1.001 7500

beta[1,5] 1.014 0.741 0.980 -0.313 2.585 1.001 7500

beta[2,5] 1.144 0.754 1.143 -0.295 2.618 1.001 7500

pi 0.033 0.006 0.032 0.025 0.047 1.001 7500

se[1,1] 0.220 0.036 0.218 0.153 0.297 1.001 7500

se[2,1] 0.701 0.041 0.702 0.618 0.780 1.001 7500

se[3,1] 0.858 0.035 0.860 0.785 0.918 1.001 7500

se[4,1] 0.646 0.083 0.656 0.457 0.783 1.002 6500

se[5,1] 0.897 0.093 0.924 0.645 0.997 1.003 3500

sp[1,1] 0.784 0.006 0.784 0.772 0.796 1.001 7500

sp[2,1] 0.633 0.007 0.633 0.619 0.647 1.001 7500

sp[3,1] 0.536 0.008 0.536 0.521 0.551 1.001 3100

sp[4,1] 0.994 0.001 0.994 0.992 0.997 1.001 7500

sp[5,1] 0.995 0.002 0.995 0.991 0.999 1.001 7500

deviance 132.174 5.603 131.419 123.292 144.753 1.001 7500

For each parameter, n.eff is a crude measure of effective sample size,

and Rhat is the potential scale reduction factor (at convergence, Rhat=1).

DIC info (using the rule, pD = var(deviance)/2)

pD = 15.7 and DIC = 147.9

DIC is an estimate of expected predictive error (lower deviance is better).

- 1. Analysis assuming the participants with missing values in Y_5_ and Y_6_ were negative for Y_5_ and Y_6_ (Results based on the analysis of 68^th^ replicate dataset randomly chosen from among the model results of the 100 replicate datasets)

Inference for Bugs model at "sim_model1_NN.txt", fit using jags,

3 chains, each with 50000 iterations (first 25000 discarded), n.thin = 10 n.sims = 7500 iterations saved

mu.vect sd.vect 50% 2.5% 97.5% Rhat n.eff

a_prev -1.969 0.088 -1.975 -2.121 -1.772 1.002 1600

alpha[1,1] -1.157 0.016 -1.157 -1.190 -1.125 1.001 7500

alpha[1,2] -0.970 0.018 -0.970 -1.007 -0.937 1.001 3600

alpha[2,2] 1.217 0.040 1.216 1.140 1.297 1.001 7500

alpha[1,3] -1.486 0.023 -1.486 -1.532 -1.441 1.001 7500

alpha[2,3] 1.710 0.062 1.711 1.592 1.831 1.001 7500

alpha[3,3] 3.475 0.176 3.454 3.288 3.759 1.041 1400

alpha[1,4] -2.809 0.085 -2.804 -2.989 -2.656 1.001 4000

alpha[1,5] -2.753 0.085 -2.748 -2.934 -2.603 1.001 7500

beta[1,1] -0.846 0.123 -0.846 -1.088 -0.605 1.001 4600

beta[1,2] 0.714 0.178 0.701 0.407 1.095 1.001 7500

beta[1,3] 0.479 0.217 0.474 0.068 0.910 1.001 4700

beta[2,3] 0.188 0.577 0.156 -0.856 1.358 1.001 6600

beta[1,4] 0.458 0.247 0.479 -0.088 0.883 1.003 1800

beta[1,5] 0.273 0.540 0.243 -0.709 1.405 1.002 2400

beta[2,5] 0.719 0.555 0.739 -0.428 1.742 1.002 2600

pi 0.025 0.005 0.024 0.017 0.038 1.002 1700

se[1,1] 0.201 0.034 0.199 0.138 0.273 1.001 5000

se[2,1] 0.759 0.053 0.758 0.658 0.863 1.001 7500

se[3,1] 0.703 0.127 0.722 0.418 0.893 1.001 7500

se[4,1] 0.672 0.089 0.684 0.465 0.811 1.006 1200

se[5,1] 0.744 0.093 0.757 0.519 0.884 1.008 1100

sp[1,1] 0.876 0.003 0.876 0.870 0.883 1.001 7500

sp[2,1] 0.781 0.005 0.781 0.772 0.790 1.002 3700

sp[3,1] 0.705 0.005 0.705 0.695 0.715 1.001 5800

sp[4,1] 0.997 0.001 0.997 0.996 0.999 1.001 6500

sp[5,1] 0.997 0.001 0.997 0.995 0.998 1.001 7500

deviance 134.303 5.555 133.653 125.437 146.878 1.001 7500

For each parameter, n.eff is a crude measure of effective sample size,

and Rhat is the potential scale reduction factor (at convergence, Rhat=1).

DIC info (using the rule, pD = var(deviance)/2)

pD = 15.4 and DIC = 149.7

DIC is an estimate of expected predictive error (lower deviance is better).

- 1. Analysis with simultaneous imputation of missing values in Y5 and Y6 assuming the data were MAR (Results based on the analysis of the 67th replicate dataset randomly chosen from among the model results of the 100 replicate datasets)

Inference for Bugs model at "sim_model1_MAR_3_groups.txt", fit using jags, 3 chains, each with 20000 iterations (first 10000 discarded), n.thin = 10 n.sims = 3000 iterations saved

mu.vect sd.vect 50% 2.5% 97.5% Rhat n.eff

a_prev[1] -1.823 0.053 -1.823 -1.925 -1.718 1.001 3000

a_prev[2] -0.390 0.382 -0.384 -1.132 0.394 1.007 370

a_prev[3] -1.663 0.268 -1.649 -2.237 -1.184 1.001 3000

alpha[1,1,1] -0.785 0.019 -0.785 -0.823 -0.748 1.001 3000

alpha[1,2,1] -0.544 0.021 -0.544 -0.586 -0.503 1.001 3000

alpha[2,2,1] 0.801 0.042 0.802 0.723 0.885 1.001 3000

alpha[1,3,1] -1.336 0.033 -1.336 -1.400 -1.273 1.003 720

alpha[2,3,1] 1.604 0.067 1.605 1.470 1.734 1.002 1200

alpha[3,3,1] 3.131 0.076 3.131 2.983 3.283 1.003 790

alpha[1,4,1] -2.604 0.101 -2.596 -2.818 -2.427 1.001 2000

alpha[1,5,1] -2.599 0.126 -2.584 -2.872 -2.395 1.001 3000

alpha[1,1,2] -0.987 0.065 -0.984 -1.124 -0.869 1.005 460

alpha[1,2,2] -0.563 0.056 -0.562 -0.677 -0.456 1.001 2800

alpha[2,2,2] 0.724 0.145 0.721 0.458 1.016 1.001 3000

alpha[1,3,2] -1.029 0.081 -1.026 -1.198 -0.883 1.008 460

alpha[2,3,2] 0.983 0.205 0.987 0.562 1.369 1.001 3000

alpha[3,3,2] 2.940 0.243 2.917 2.525 3.483 1.003 830

alpha[1,4,2] -2.604 0.101 -2.596 -2.818 -2.427 1.001 2000

alpha[1,5,2] -2.599 0.126 -2.584 -2.872 -2.395 1.001 3000

alpha[1,1,3] -3.593 0.322 -3.550 -4.332 -3.084 1.001 3000

alpha[1,2,3] -3.597 0.313 -3.565 -4.304 -3.084 1.001 3000

alpha[2,2,3] 0.006 1.022 0.030 -2.069 1.912 1.001 3000

alpha[1,3,3] -1.639 0.034 -1.639 -1.708 -1.573 1.001 2200

alpha[2,3,3] -0.004 0.989 -0.002 -1.968 1.934 1.001 3000

alpha[3,3,3] -0.010 0.980 -0.007 -1.917 1.951 1.001 2300

alpha[1,4,3] -2.604 0.101 -2.596 -2.818 -2.427 1.001 2000

alpha[1,5,3] -2.599 0.126 -2.584 -2.872 -2.395 1.001 3000

beta[1,1,1] -0.949 0.124 -0.947 -1.195 -0.711 1.001 3000

beta[1,2,1] 0.507 0.114 0.506 0.293 0.737 1.001 3000

beta[1,3,1] 0.657 0.200 0.651 0.272 1.055 1.001 2400

beta[2,3,1] 1.051 0.258 1.047 0.541 1.561 1.001 3000

beta[1,4,1] 0.436 0.166 0.437 0.108 0.762 1.001 2500

beta[1,5,1] 0.551 0.328 0.547 -0.067 1.234 1.001 3000

beta[2,5,1] 0.600 0.326 0.606 -0.026 1.224 1.001 2300

beta[1,1,2] -0.095 0.448 -0.089 -0.963 0.821 1.004 600

beta[1,2,2] -0.139 0.465 -0.138 -1.041 0.771 1.001 3000

beta[1,3,2] 0.182 0.450 0.184 -0.694 1.046 1.002 1200

beta[2,3,2] 0.187 0.463 0.189 -0.721 1.090 1.003 950

beta[1,4,2] 0.436 0.166 0.437 0.108 0.762 1.001 2500

beta[1,5,2] 0.551 0.328 0.547 -0.067 1.234 1.001 3000

beta[2,5,2] 0.600 0.326 0.606 -0.026 1.224 1.001 2300

beta[1,1,3] -0.041 0.455 -0.043 -0.924 0.854 1.001 3000

beta[1,2,3] -0.037 0.453 -0.044 -0.919 0.843 1.002 2000

beta[1,3,3] 0.005 0.448 0.005 -0.885 0.869 1.002 2000

beta[2,3,3] 0.000 0.454 0.001 -0.886 0.892 1.002 1000

beta[1,4,3] 0.436 0.166 0.437 0.108 0.762 1.001 2500

beta[1,5,3] 0.551 0.328 0.547 -0.067 1.234 1.001 3000

beta[2,5,3] 0.600 0.326 0.606 -0.026 1.224 1.001 2300

pi[1] 0.034 0.004 0.034 0.027 0.043 1.001 3000

pi[2] 0.019 0.019 0.014 0.001 0.075 1.006 410

pi[3] 0.000 0.000 0.000 0.000 0.001 1.001 3000

pi[4] 0.020 0.004 0.019 0.014 0.029 1.008 400

pv[1] 0.484 0.005 0.484 0.474 0.494 1.001 3000

pv[2] 0.150 0.004 0.150 0.143 0.157 1.001 3000

pv[3] 0.366 0.005 0.366 0.357 0.375 1.001 3000

se[1,1] 0.173 0.031 0.172 0.116 0.239 1.001 3000

se[2,1] 0.693 0.040 0.694 0.615 0.769 1.001 3000

se[3,1] 0.887 0.028 0.889 0.827 0.936 1.001 3000

se[4,1] 0.667 0.059 0.669 0.543 0.777 1.002 2000

se[5,1] 0.810 0.060 0.814 0.683 0.914 1.002 1100

se[1,2] 0.465 0.163 0.464 0.168 0.794 1.004 580

se[2,2] 0.450 0.168 0.445 0.149 0.780 1.001 3000

se[3,2] 0.592 0.174 0.603 0.237 0.893 1.003 870

se[4,2] 0.667 0.059 0.669 0.543 0.777 1.002 2000

se[5,2] 0.810 0.060 0.814 0.683 0.914 1.002 1100

se[1,3] 0.485 0.166 0.483 0.178 0.803 1.001 3000

se[2,3] 0.486 0.165 0.482 0.179 0.800 1.002 1600

se[3,3] 0.502 0.177 0.503 0.168 0.830 1.002 1000

se[4,3] 0.667 0.059 0.669 0.543 0.777 1.002 2000

se[5,3] 0.810 0.060 0.814 0.683 0.914 1.002 1100

se[1,4] 0.214 0.047 0.208 0.138 0.325 1.004 590

se[2,4] 0.656 0.054 0.660 0.531 0.745 1.003 1000

se[3,4] 0.851 0.038 0.855 0.764 0.914 1.002 1100

se[4,4] 0.667 0.059 0.669 0.543 0.777 1.002 2000

se[5,4] 0.810 0.060 0.814 0.683 0.914 1.002 1100

sp[1,1] 0.784 0.006 0.784 0.773 0.795 1.001 3000

sp[2,1] 0.640 0.007 0.640 0.627 0.653 1.001 3000

sp[3,1] 0.546 0.007 0.546 0.533 0.559 1.001 3000

sp[4,1] 0.995 0.001 0.995 0.992 0.998 1.002 2000

sp[5,1] 0.995 0.002 0.995 0.992 0.998 1.001 3000

sp[1,2] 0.838 0.016 0.837 0.808 0.870 1.005 480

sp[2,2] 0.668 0.018 0.668 0.632 0.705 1.001 3000

sp[3,2] 0.550 0.020 0.549 0.513 0.593 1.004 760

sp[4,2] 0.995 0.001 0.995 0.992 0.998 1.002 2000

sp[5,2] 0.995 0.002 0.995 0.992 0.998 1.001 3000

sp[1,3] 1.000 0.000 1.000 0.999 1.000 1.002 1600

sp[2,3] 1.000 0.000 1.000 0.999 1.000 1.001 3000

sp[3,3] 0.949 0.004 0.949 0.942 0.956 1.001 2200

sp[4,3] 0.995 0.001 0.995 0.992 0.998 1.002 2000

sp[5,3] 0.995 0.002 0.995 0.992 0.998 1.001 3000

sp[1,4] 0.873 0.004 0.873 0.865 0.880 1.004 650

sp[2,4] 0.779 0.005 0.779 0.769 0.788 1.001 3000

sp[3,4] 0.697 0.005 0.697 0.687 0.708 1.003 800

sp[4,4] 0.995 0.001 0.995 0.992 0.998 1.002 2000

sp[5,4] 0.995 0.002 0.995 0.992 0.998 1.001 3000

deviance 20188.474 63.250 20190.064 20055.798 20307.394 1.002 1900

For each parameter, n.eff is a crude measure of effective sample size,

and Rhat is the potential scale reduction factor (at convergence, Rhat=1).

DIC info (using the rule, pD = var(deviance)/2)

pD = 1999.6 and DIC = 22188.0

DIC is an estimate of expected predictive error (lower deviance is better).

- 1. Analysis with simultaneous imputation of missing values in Y5 and Y6 assuming the data were MNAR (Results based on the analysis of the 6^th^ replicate dataset randomly chosen from among the model results of the 100 replicate datasets)

Inference for Bugs model at "sim_model1_MNAR_3_groups.txt", fit using jags, 3 chains, each with 18000 iterations (first 9000 discarded), n.thin = 10 n.sims = 2700 iterations saved

mu.vect sd.vect 50% 2.5% 97.5% Rhat n.eff

a_prev -1.846 0.056 -1.847 -1.953 -1.736 1.002 990

alpha[1,1,1] -0.821 0.020 -0.820 -0.861 -0.782 1.003 730

alpha[1,2,1] -0.515 0.020 -0.515 -0.554 -0.476 1.001 2700

alpha[2,2,1] 0.752 0.042 0.752 0.669 0.832 1.001 2700

alpha[1,3,1] -1.245 0.032 -1.245 -1.308 -1.182 1.002 1200

alpha[2,3,1] 1.523 0.068 1.523 1.390 1.658 1.002 1400

alpha[3,3,1] 3.153 0.081 3.152 3.003 3.318 1.003 770

alpha[1,4,1] -2.577 0.096 -2.569 -2.784 -2.415 1.001 2700

alpha[1,5,1] -2.393 0.100 -2.386 -2.622 -2.222 1.001 2700

alpha[1,1,2] -0.841 0.054 -0.840 -0.952 -0.742 1.001 2700

alpha[1,2,2] -0.542 0.054 -0.541 -0.650 -0.440 1.001 2700

alpha[2,2,2] 0.831 0.117 0.833 0.599 1.055 1.002 1000

alpha[1,3,2] -1.212 0.081 -1.211 -1.373 -1.055 1.001 2700

alpha[2,3,2] 1.498 0.193 1.500 1.123 1.874 1.001 2700

alpha[3,3,2] 3.047 0.224 3.030 2.665 3.534 1.001 2000

alpha[1,4,2] -2.577 0.096 -2.569 -2.784 -2.415 1.001 2700

alpha[1,5,2] -2.393 0.100 -2.386 -2.622 -2.222 1.001 2700

alpha[1,1,3] -3.601 0.331 -3.555 -4.397 -3.073 1.001 2700

alpha[1,2,3] -3.604 0.317 -3.560 -4.327 -3.103 1.001 1900

alpha[2,2,3] -0.012 0.979 -0.007 -1.899 1.877 1.002 1200

alpha[1,3,3] -1.613 0.034 -1.612 -1.680 -1.549 1.001 2700

alpha[2,3,3] -0.019 0.993 -0.019 -1.989 1.906 1.001 2700

alpha[3,3,3] 0.004 1.010 -0.004 -1.879 1.957 1.001 2700

alpha[1,4,3] -2.577 0.096 -2.569 -2.784 -2.415 1.001 2700

alpha[1,5,3] -2.393 0.100 -2.386 -2.622 -2.222 1.001 2700

b_psi[1,1] -0.001 0.450 -0.003 -0.916 0.846 1.002 1400

b_psi[2,1] -0.008 0.444 -0.002 -0.868 0.870 1.001 2700

b_psi[1,2] 0.001 0.441 0.001 -0.883 0.886 1.000 2700

b_psi[2,2] 0.010 0.444 0.014 -0.893 0.852 1.001 2700

b_psi[1,3] 0.017 0.435 0.019 -0.831 0.900 1.001 2700

b_psi[2,3] 0.002 0.443 0.008 -0.865 0.868 1.002 2700

b_psi[1,4] -0.002 0.446 0.002 -0.875 0.849 1.001 2700

b_psi[2,4] -0.018 0.439 -0.027 -0.857 0.840 1.001 2700

b_tau[1] -0.165 0.456 -0.173 -1.037 0.729 1.001 2600

b_tau[2] -1.992 0.282 -1.988 -2.556 -1.452 1.001 2700

beta[1,1,1] -0.918 0.130 -0.915 -1.180 -0.675 1.001 2700

beta[1,2,1] 0.449 0.113 0.449 0.225 0.669 1.001 2300

beta[1,3,1] 0.446 0.192 0.445 0.090 0.823 1.000 2700

beta[2,3,1] 1.238 0.260 1.224 0.745 1.760 1.001 2700

beta[1,4,1] 0.583 0.197 0.579 0.199 0.972 1.002 1000

beta[1,5,1] 0.640 0.346 0.642 0.002 1.321 1.005 470

beta[2,5,1] 0.719 0.345 0.722 0.038 1.404 1.003 860

beta[1,1,2] -0.099 0.451 -0.105 -0.961 0.809 1.003 700

beta[1,2,2] -0.131 0.460 -0.132 -1.006 0.816 1.001 2700

beta[1,3,2] 0.156 0.449 0.150 -0.733 1.040 1.001 2700

beta[2,3,2] 0.158 0.451 0.171 -0.782 1.016 1.002 1100

beta[1,4,2] 0.583 0.197 0.579 0.199 0.972 1.002 1000

beta[1,5,2] 0.640 0.346 0.642 0.002 1.321 1.005 470

beta[2,5,2] 0.719 0.345 0.722 0.038 1.404 1.003 860

beta[1,1,3] -0.138 0.450 -0.136 -1.003 0.777 1.001 2700

beta[1,2,3] -0.133 0.456 -0.128 -1.000 0.782 1.000 2700

beta[1,3,3] 0.007 0.446 0.009 -0.867 0.900 1.001 1900

beta[2,3,3] -0.010 0.452 -0.016 -0.890 0.844 1.004 590

beta[1,4,3] 0.583 0.197 0.579 0.199 0.972 1.002 1000

beta[1,5,3] 0.640 0.346 0.642 0.002 1.321 1.005 470

beta[2,5,3] 0.719 0.345 0.722 0.038 1.404 1.003 860

pi[1] 0.033 0.004 0.032 0.025 0.041 1.002 990

pi[2] 0.014 0.006 0.014 0.005 0.026 1.002 1500

pi[3] 0.001 0.001 0.001 0.000 0.002 1.001 2700

pi[4] 0.018 0.002 0.018 0.014 0.023 1.003 900

psi[1,1] 0.500 0.164 0.499 0.180 0.801 1.002 1400

psi[2,1] 0.497 0.162 0.499 0.193 0.808 1.001 2700

psi[1,2] 0.500 0.161 0.500 0.189 0.812 1.002 2700

psi[2,2] 0.504 0.162 0.506 0.186 0.803 1.001 2700

psi[1,3] 0.506 0.159 0.508 0.203 0.816 1.001 2700

psi[2,3] 0.501 0.161 0.503 0.193 0.807 1.002 2700

psi[1,4] 0.499 0.163 0.501 0.191 0.802 1.001 2700

psi[2,4] 0.493 0.161 0.489 0.196 0.800 1.002 1700

pv[1] 0.476 0.005 0.476 0.466 0.486 1.001 2700

pv[2] 0.150 0.004 0.150 0.143 0.157 1.001 2700

pv[3] 0.374 0.005 0.374 0.365 0.383 1.002 1600

se[1,1] 0.181 0.034 0.180 0.119 0.250 1.001 2700

se[2,1] 0.672 0.041 0.673 0.589 0.748 1.001 2700

se[3,1] 0.858 0.032 0.860 0.790 0.914 1.001 2700

se[4,1] 0.716 0.065 0.719 0.579 0.835 1.002 1100

se[5,1] 0.853 0.056 0.858 0.727 0.945 1.004 630

se[1,2] 0.464 0.164 0.458 0.168 0.791 1.003 690

se[2,2] 0.452 0.166 0.448 0.157 0.793 1.001 2700

se[3,2] 0.582 0.173 0.590 0.219 0.881 1.001 2600

se[4,2] 0.716 0.065 0.719 0.579 0.835 1.002 1100

se[5,2] 0.853 0.056 0.858 0.727 0.945 1.004 630

se[1,3] 0.450 0.163 0.446 0.158 0.781 1.002 2700

se[2,3] 0.451 0.166 0.449 0.159 0.783 1.000 2700

se[3,3] 0.501 0.173 0.500 0.188 0.834 1.001 2700

se[4,3] 0.716 0.065 0.719 0.579 0.835 1.002 1100

se[5,3] 0.853 0.056 0.858 0.727 0.945 1.004 630

se[1,4] 0.219 0.037 0.218 0.152 0.294 1.001 2700

se[2,4] 0.641 0.042 0.642 0.555 0.720 1.001 2700

se[3,4] 0.819 0.036 0.821 0.743 0.883 1.001 2300

se[4,4] 0.716 0.065 0.719 0.579 0.835 1.002 1100

se[5,4] 0.853 0.056 0.858 0.727 0.945 1.004 630

sp[1,1] 0.794 0.006 0.794 0.783 0.805 1.003 720

sp[2,1] 0.637 0.007 0.637 0.624 0.649 1.002 1500

sp[3,1] 0.534 0.007 0.534 0.520 0.547 1.002 980

sp[4,1] 0.995 0.001 0.995 0.992 0.997 1.001 2700

sp[5,1] 0.991 0.002 0.991 0.987 0.996 1.001 2700

sp[1,2] 0.799 0.015 0.799 0.771 0.829 1.001 2700

sp[2,2] 0.642 0.018 0.642 0.608 0.677 1.000 2700

sp[3,2] 0.539 0.019 0.539 0.505 0.577 1.000 2700

sp[4,2] 0.995 0.001 0.995 0.992 0.997 1.001 2700

sp[5,2] 0.991 0.002 0.991 0.987 0.996 1.001 2700

sp[1,3] 1.000 0.000 1.000 0.999 1.000 1.001 2700

sp[2,3] 1.000 0.000 1.000 0.999 1.000 1.001 2700

sp[3,3] 0.946 0.004 0.946 0.939 0.954 1.001 2700

sp[4,3] 0.995 0.001 0.995 0.992 0.997 1.001 2700

sp[5,3] 0.991 0.002 0.991 0.987 0.996 1.001 2700

sp[1,4] 0.873 0.004 0.873 0.866 0.880 1.003 860

sp[2,4] 0.776 0.004 0.776 0.767 0.785 1.002 1300

sp[3,4] 0.692 0.005 0.691 0.682 0.702 1.003 870

sp[4,4] 0.995 0.001 0.995 0.992 0.997 1.001 2700

sp[5,4] 0.991 0.002 0.991 0.987 0.996 1.001 2700

tau[1] 0.440 0.164 0.431 0.150 0.767 1.001 2700

tau[2] 0.028 0.018 0.023 0.005 0.073 1.001 2700

deviance 20283.066 60.924 20284.812 20154.844 20397.994 1.001 2700

For each parameter, n.eff is a crude measure of effective sample size,

and Rhat is the potential scale reduction factor (at convergence, Rhat=1).

DIC info (using the rule, pD = var(deviance)/2)

pD = 1856.7 and DIC = 22139.8

DIC is an estimate of expected predictive error (lower deviance is better).

1. Analysis of Vukuzazi data
2. Model results for the Vukuzazi data based on the composite reference standard (Table 4 in the main document).
   - 1. Complete-case analysis

Inference for Bugs model at "model.naive.txt", fit using jags,

3 chains, each with 50000 iterations (first 25000 discarded), n.thin = 10 n.sims = 7500 iterations saved

mu.vect sd.vect 50% 2.5% 97.5% Rhat n.eff

pi 0.014 0.002 0.014 0.011 0.017 1.001 7500

se[1] 0.181 0.046 0.177 0.101 0.278 1.001 5400

se[2] 0.822 0.046 0.825 0.724 0.904 1.001 4700

se[3] 0.898 0.036 0.901 0.816 0.957 1.001 3500

se[4] 0.577 0.059 0.578 0.462 0.689 1.001 7500

se[5] 0.726 0.054 0.728 0.614 0.825 1.001 7500

sp[1] 0.830 0.005 0.830 0.820 0.841 1.001 7500

sp[2] 0.671 0.007 0.671 0.658 0.684 1.001 7300

sp[3] 0.546 0.007 0.546 0.532 0.560 1.001 7500

sp[4] 1.000 0.000 1.000 0.999 1.000 1.001 7500

sp[5] 1.000 0.000 1.000 0.999 1.000 1.001 7500

deviance 534.490 5.976 533.823 524.770 547.828 1.001 5800

For each parameter, n.eff is a crude measure of effective sample size,

and Rhat is the potential scale reduction factor (at convergence, Rhat=1).

DIC info (using the rule, pD = var(deviance)/2)

pD = 17.9 and DIC = 552.3

DIC is an estimate of expected predictive error (lower deviance is better).

- - 1. Analysis assuming the participants with missing Xpert Ultra and culture test results were negative for Xpert Ultra and culture

Inference for Bugs model at "model.naive.txt", fit using jags,

3 chains, each with 50000 iterations (first 25000 discarded), n.thin = 10 n.sims = 7500 iterations saved

mu.vect sd.vect 50% 2.5% 97.5% Rhat n.eff

pi 0.007 0.001 0.007 0.005 0.009 1.001 7500

se[1] 0.179 0.046 0.176 0.097 0.277 1.001 7500

se[2] 0.819 0.046 0.822 0.721 0.898 1.001 7500

se[3] 0.895 0.037 0.899 0.815 0.957 1.001 2900

se[4] 0.576 0.060 0.576 0.458 0.689 1.001 6000

se[5] 0.723 0.054 0.726 0.612 0.822 1.001 7500

sp[1] 0.894 0.003 0.895 0.888 0.900 1.001 7500

sp[2] 0.805 0.004 0.805 0.797 0.813 1.001 7500

sp[3] 0.647 0.005 0.647 0.638 0.657 1.001 7500

sp[4] 1.000 0.000 1.000 0.999 1.000 1.001 7500

sp[5] 1.000 0.000 1.000 0.999 1.000 1.001 3600

deviance 1094.781 6.082 1094.234 1084.886 1108.526 1.001 7500

For each parameter, n.eff is a crude measure of effective sample size,

and Rhat is the potential scale reduction factor (at convergence, Rhat=1).

DIC info (using the rule, pD = var(deviance)/2)

pD = 18.5 and DIC = 1113.3

DIC is an estimate of expected predictive error (lower deviance is better).

- - 1. Analysis following multiple imputation of missing Xpert Ultra and culture test results for ALL the participants with unconfirmed TB status (Results below based on the 37^th^ imputed dataset randomly chosen from among the model results of the 100 imputed datasets)

Inference for Bugs model at "model.naive", fit using jags,

3 chains, each with 50000 iterations (first 25000 discarded), n.thin = 10 n.sims = 7500 iterations saved

mu.vect sd.vect 50% 2.5% 97.5% Rhat n.eff

pi 0.011 0.001 0.011 0.009 0.013 1.001 7500

se[1] 0.167 0.036 0.165 0.104 0.244 1.001 4500

se[2] 0.632 0.046 0.633 0.539 0.720 1.002 2300

se[3] 0.708 0.043 0.709 0.619 0.787 1.001 5300

se[4] 0.480 0.048 0.479 0.387 0.573 1.001 7500

se[5] 0.753 0.041 0.754 0.668 0.829 1.001 6700

sp[1] 0.895 0.003 0.895 0.888 0.901 1.001 7400

sp[2] 0.806 0.004 0.806 0.798 0.814 1.001 7500

sp[3] 0.648 0.005 0.648 0.638 0.657 1.001 7500

sp[4] 1.000 0.000 1.000 0.999 1.000 1.001 6600

sp[5] 1.000 0.000 1.000 0.999 1.000 1.001 7500

deviance 6854.784 6.021 6854.167 6845.138 6868.310 1.001 7500

For each parameter, n.eff is a crude measure of effective sample size,

and Rhat is the potential scale reduction factor (at convergence, Rhat=1).

DIC info (using the rule, pD = var(deviance)/2)

pD = 18.1 and DIC = 6872.9

DIC is an estimate of expected predictive error (lower deviance is better).

- - 1. Analysis following multiple imputation of missing Xpert Ultra and culture test results for **ONLY** the eligible participants with unconfirmed TB status (Results based on the 75th imputed dataset randomly chosen from among the model results of the 100 imputed datasets)

Inference for Bugs model at "model.naive", fit using jags,

3 chains, each with 50000 iterations (first 25000 discarded), n.thin = 10 n.sims = 7500 iterations saved

mu.vect sd.vect 50% 2.5% 97.5% Rhat n.eff

pi 0.008 0.001 0.008 0.007 0.010 1.001 5100

se[1] 0.149 0.039 0.147 0.082 0.234 1.001 7500

se[2] 0.774 0.046 0.777 0.678 0.857 1.001 5700

se[3] 0.888 0.035 0.891 0.812 0.948 1.001 7500

se[4] 0.570 0.054 0.571 0.462 0.674 1.001 5600

se[5] 0.742 0.048 0.744 0.643 0.830 1.001 7500

sp[1] 0.894 0.003 0.894 0.888 0.901 1.001 5800

sp[2] 0.806 0.004 0.806 0.798 0.814 1.001 7500

sp[3] 0.648 0.005 0.648 0.639 0.658 1.002 2300

sp[4] 1.000 0.000 1.000 0.999 1.000 1.001 6400

sp[5] 1.000 0.000 1.000 0.999 1.000 1.001 6900

deviance 6770.068 6.036 6769.324 6760.458 6783.258 1.001 7000

For each parameter, n.eff is a crude measure of effective sample size,

and Rhat is the potential scale reduction factor (at convergence, Rhat=1).

DIC info (using the rule, pD = var(deviance)/2)

pD = 18.2 and DIC = 6788.3

DIC is an estimate of expected predictive error (lower deviance is better).

1. Model results for the Vukuzazi data based on Bayesian LCA (Table 5 in the main document)
   - 1. Complete-case analysis

Inference for Bugs model at "model1.txt", fit using jags,

3 chains, each with 50000 iterations (first 25000 discarded), n.thin = 10 n.sims = 7500 iterations saved

mu.vect sd.vect 50% 2.5% 97.5% Rhat n.eff

a_prev -2.218 0.086 -2.228 -2.357 -2.023 1.003 2900

alpha[1,1] -0.955 0.021 -0.955 -0.996 -0.913 1.001 7500

alpha[1,2] -0.430 0.021 -0.430 -0.471 -0.390 1.001 7500

alpha[2,2] -0.109 0.051 -0.109 -0.209 -0.010 1.001 6000

alpha[1,3] -0.365 0.024 -0.365 -0.412 -0.318 1.001 7500

alpha[2,3] 0.046 0.050 0.046 -0.053 0.143 1.001 3700

alpha[3,3] 0.722 0.040 0.722 0.643 0.800 1.001 4000

alpha[1,4] -3.376 0.202 -3.361 -3.810 -3.025 1.001 3000

alpha[1,5] -2.982 0.150 -2.968 -3.314 -2.728 1.002 2700

beta[1,1] -0.881 0.199 -0.874 -1.279 -0.496 1.001 7500

beta[1,2] 1.433 0.296 1.414 0.905 2.069 1.001 7500

beta[1,3] 1.173 0.641 1.143 -0.014 2.471 1.002 2500

beta[2,3] 0.877 0.665 0.889 -0.466 2.130 1.001 3300

beta[1,4] 0.214 0.272 0.236 -0.375 0.696 1.002 1300

beta[1,5] 0.471 0.600 0.453 -0.645 1.706 1.002 1300

beta[2,5] -0.127 0.610 -0.113 -1.345 1.024 1.003 1100

pi 0.014 0.003 0.013 0.009 0.022 1.002 3100

se[1] 0.194 0.054 0.191 0.100 0.310 1.001 7500

se[2] 0.916 0.043 0.921 0.817 0.981 1.001 7100

se[3] 0.961 0.027 0.967 0.894 0.995 1.001 3000

se[4] 0.583 0.103 0.593 0.354 0.757 1.003 1200

se[5] 0.629 0.103 0.641 0.397 0.796 1.002 3000

sp[1] 0.830 0.005 0.830 0.819 0.840 1.001 7500

sp[2] 0.673 0.007 0.673 0.659 0.687 1.001 7500

sp[3] 0.547 0.007 0.547 0.533 0.561 1.001 7500

sp[4] 1.000 0.000 1.000 0.999 1.000 1.001 3700

sp[5] 0.998 0.001 0.998 0.997 1.000 1.001 4300

deviance 137.529 5.269 136.879 129.064 149.624 1.001 7500

For each parameter, n.eff is a crude measure of effective sample size,

and Rhat is the potential scale reduction factor (at convergence, Rhat=1).

DIC info (using the rule, pD = var(deviance)/2)

pD = 13.9 and DIC = 151.4

DIC is an estimate of expected predictive error (lower deviance is better).

- - 1. Analysis assuming the participants with missing Xpert Ultra and culture test results were negative for Xpert Ultra and culture

Inference for Bugs model at "model1_NN.txt", fit using jags,

3 chains, each with 50000 iterations (first 25000 discarded), n.thin = 10 n.sims = 7500 iterations saved

mu.vect sd.vect 50% 2.5% 97.5% Rhat n.eff

a_prev -2.455 0.077 -2.464 -2.582 -2.269 1.001 3700

alpha[1,1] -1.252 0.017 -1.252 -1.285 -1.219 1.001 7500

alpha[1,2] -0.907 0.016 -0.907 -0.939 -0.876 1.002 1400

alpha[2,2] 0.353 0.045 0.353 0.265 0.440 1.001 7500

alpha[1,3] -0.596 0.016 -0.596 -0.627 -0.567 1.002 2200

alpha[2,3] 0.207 0.043 0.206 0.121 0.291 1.001 7500

alpha[3,3] 0.929 0.034 0.929 0.862 0.996 1.001 7500

alpha[1,4] -3.527 0.174 -3.512 -3.898 -3.223 1.001 6100

alpha[1,5] -3.208 0.133 -3.196 -3.496 -2.974 1.001 7500

beta[1,1] -0.872 0.193 -0.869 -1.262 -0.503 1.001 3200

beta[1,2] 1.411 0.280 1.389 0.911 2.010 1.002 2800

beta[1,3] 1.278 0.629 1.250 0.120 2.565 1.001 6900

beta[2,3] 0.787 0.652 0.801 -0.523 2.005 1.001 7500

beta[1,4] 0.163 0.267 0.183 -0.417 0.639 1.003 1200

beta[1,5] 0.439 0.601 0.412 -0.649 1.705 1.003 1100

beta[2,5] -0.105 0.611 -0.078 -1.360 1.030 1.003 960

pi 0.007 0.002 0.007 0.005 0.012 1.001 3900

se[1] 0.196 0.052 0.192 0.104 0.307 1.001 3200

se[2] 0.913 0.041 0.918 0.819 0.978 1.001 2900

se[3] 0.964 0.025 0.969 0.903 0.995 1.001 7500

se[4] 0.563 0.102 0.572 0.338 0.739 1.003 1100

se[5] 0.622 0.106 0.635 0.379 0.795 1.003 1200

sp[1] 0.895 0.003 0.895 0.889 0.901 1.001 7500

sp[2] 0.806 0.004 0.806 0.798 0.815 1.002 1200

sp[3] 0.648 0.005 0.648 0.638 0.658 1.003 920

sp[4] 1.000 0.000 1.000 0.999 1.000 1.001 6100

sp[5] 0.999 0.000 0.999 0.999 1.000 1.001 7500

deviance 125.059 5.339 124.443 116.485 137.309 1.001 4800

For each parameter, n.eff is a crude measure of effective sample size,

and Rhat is the potential scale reduction factor (at convergence, Rhat=1).

DIC info (using the rule, pD = var(deviance)/2)

pD = 14.3 and DIC = 139.3

DIC is an estimate of expected predictive error (lower deviance is better).

- - 1. Analysis following multiple imputation of missing Xpert Ultra and culture test results for ALL the participants with unconfirmed TB status (Results based on the 90^th^ imputed dataset randomly chosen from among the model results of the 100 imputed datasets)

Inference for Bugs model at "model1_MICE_lca.txt", fit using jags,

3 chains, each with 50000 iterations (first 25000 discarded), n.thin = 10 n.sims = 7500 iterations saved

mu.vect sd.vect 50% 2.5% 97.5% Rhat n.eff

a_prev[1] -2.229 0.079 -2.235 -2.366 -2.059 1.002 2600

a_prev[2] -0.249 0.198 -0.246 -0.637 0.131 1.002 2400

a_prev[3] -1.185 0.332 -1.148 -1.963 -0.654 1.001 7500

alpha[1,1] -0.957 0.021 -0.956 -0.999 -0.914 1.001 7500

alpha[2,1] -0.102 0.046 -0.102 -0.193 -0.014 1.001 3100

alpha[3,1] -2.747 0.380 -2.692 -3.641 -2.169 1.001 7500

alpha[1,2] -0.441 0.020 -0.442 -0.481 -0.402 1.001 7500

alpha[2,2] -0.044 0.045 -0.044 -0.133 0.045 1.001 5600

alpha[3,2] -0.355 0.042 -0.356 -0.440 -0.274 1.001 7500

alpha[4,2] -3.184 0.334 -3.144 -3.956 -2.643 1.001 7300

alpha[1,3] -0.345 0.023 -0.345 -0.389 -0.299 1.001 7500

alpha[2,3] -0.004 0.043 -0.004 -0.089 0.079 1.001 7500

alpha[3,3] 0.685 0.036 0.685 0.614 0.754 1.001 7500

alpha[4,3] 0.140 0.039 0.140 0.066 0.215 1.001 7500

alpha[5,3] -0.650 0.034 -0.649 -0.717 -0.583 1.001 7500

alpha[1,4] -3.329 0.182 -3.319 -3.719 -3.001 1.001 7500

alpha[2,4] 0.658 0.450 0.735 -0.675 1.219 1.001 7500

alpha[3,4] 0.245 0.248 0.245 -0.222 0.714 1.001 5400

alpha[1,5] -2.974 0.146 -2.965 -3.298 -2.720 1.002 2200

alpha[2,5] 0.478 0.194 0.475 0.107 0.860 1.001 5000

alpha[3,5] 0.066 0.205 0.065 -0.314 0.459 1.002 2300

beta[1,1] -0.933 0.199 -0.929 -1.339 -0.557 1.001 7500

beta[2,1] -0.926 0.699 -0.882 -2.414 0.341 1.001 7500

beta[3,1] -0.356 0.880 -0.341 -2.128 1.365 1.001 3700

beta[1,2] 1.301 0.258 1.291 0.828 1.842 1.001 7500

beta[2,2] -0.911 0.544 -0.931 -1.920 0.199 1.001 7500

beta[3,2] -1.103 0.887 -1.116 -2.782 0.747 1.001 7000

beta[1,3] 1.313 0.626 1.283 0.154 2.602 1.001 3500

beta[2,3] 0.783 0.654 0.803 -0.533 2.032 1.001 6900

beta[3,3] 0.354 0.834 0.310 -1.158 2.082 1.002 2000

beta[4,3] 0.202 0.921 0.172 -1.590 2.079 1.001 4900

beta[1,4] 0.258 0.260 0.268 -0.287 0.747 1.001 3400

beta[2,4] 0.824 0.705 0.782 -0.452 2.331 1.001 3700

beta[3,4] 0.266 0.885 0.234 -1.382 2.080 1.001 7500

beta[1,5] 0.495 0.576 0.466 -0.568 1.699 1.001 5100

beta[2,5] -0.117 0.589 -0.095 -1.322 0.964 1.001 5400

beta[3,5] -0.004 0.703 -0.082 -1.165 1.641 1.001 7500

beta[4,5] 0.410 0.868 0.388 -1.250 2.157 1.001 7000

pi[1] 0.013 0.003 0.013 0.009 0.020 1.002 2500

pi[2] 0.008 0.005 0.007 0.002 0.018 1.001 4700

pi[3] 0.001 0.000 0.000 0.000 0.002 1.001 7500

pi[4] 0.007 0.002 0.007 0.004 0.012 1.001 7500

pv[1] 0.502 0.005 0.502 0.492 0.512 1.001 7500

pv[2] 0.144 0.004 0.144 0.137 0.151 1.001 7500

pv[3] 0.354 0.005 0.354 0.345 0.364 1.001 7500

se[1,1] 0.180 0.051 0.177 0.090 0.289 1.001 7500

se[2,1] 0.896 0.044 0.902 0.796 0.967 1.001 7500

se[3,1] 0.963 0.026 0.969 0.900 0.996 1.002 2800

se[4,1] 0.599 0.098 0.606 0.387 0.772 1.001 3300

se[5,1] 0.641 0.096 0.650 0.423 0.800 1.001 6800

se[1,2] 0.062 0.075 0.034 0.000 0.271 1.001 7400

se[2,2] 0.634 0.172 0.642 0.295 0.927 1.001 7500

se[3,2] 0.934 0.089 0.972 0.677 1.000 1.001 7500

se[4,2] 0.809 0.161 0.851 0.410 0.996 1.001 7500

se[5,2] 0.610 0.214 0.612 0.213 0.977 1.001 7500

se[1,3] 0.168 0.183 0.100 0.001 0.672 1.002 2300

se[2,3] 0.555 0.265 0.572 0.065 0.983 1.001 7500

se[3,3] 0.904 0.136 0.962 0.492 1.000 1.001 7500

se[4,3] 0.648 0.253 0.690 0.128 0.991 1.001 7500

se[5,3] 0.722 0.229 0.777 0.191 0.995 1.003 3100

se[1,4] 0.163 0.047 0.160 0.082 0.266 1.001 7500

se[2,4] 0.848 0.058 0.855 0.716 0.942 1.001 7500

se[3,4] 0.956 0.032 0.963 0.873 0.995 1.001 7500

se[4,4] 0.625 0.096 0.632 0.417 0.790 1.001 3600

se[5,4] 0.632 0.097 0.641 0.415 0.794 1.001 7500

sp[1,1] 0.831 0.005 0.831 0.820 0.841 1.001 7500

sp[2,1] 0.673 0.007 0.673 0.659 0.686 1.001 7500

sp[3,1] 0.547 0.007 0.547 0.533 0.562 1.001 7500

sp[4,1] 0.999 0.000 1.000 0.999 1.000 1.001 7500

sp[5,1] 0.998 0.001 0.998 0.997 1.000 1.001 4600

sp[1,2] 0.855 0.009 0.855 0.837 0.873 1.002 2500

sp[2,2] 0.789 0.011 0.789 0.767 0.810 1.001 7500

sp[3,2] 0.525 0.013 0.525 0.500 0.551 1.001 7500

sp[4,2] 0.995 0.003 0.995 0.989 1.000 1.001 6700

sp[5,2] 0.993 0.002 0.994 0.988 0.997 1.001 7500

sp[1,3] 1.000 0.000 1.000 0.999 1.000 1.001 7500

sp[2,3] 1.000 0.000 1.000 0.999 1.000 1.001 7500

sp[3,3] 0.840 0.006 0.840 0.828 0.852 1.001 7500

sp[4,3] 0.999 0.001 0.999 0.997 1.000 1.001 3500

sp[5,3] 0.998 0.001 0.998 0.996 0.999 1.001 7500

sp[1,4] 0.894 0.003 0.895 0.888 0.901 1.001 7500

sp[2,4] 0.806 0.004 0.806 0.798 0.814 1.001 7500

sp[3,4] 0.649 0.005 0.649 0.639 0.658 1.001 7500

sp[4,4] 0.999 0.000 0.999 0.998 1.000 1.001 3100

sp[5,4] 0.998 0.001 0.998 0.996 0.999 1.001 7500

deviance 2410.353 8.531 2409.811 2395.322 2428.505 1.001 7500

For each parameter, n.eff is a crude measure of effective sample size,

and Rhat is the potential scale reduction factor (at convergence, Rhat=1).

DIC info (using the rule, pD = var(deviance)/2)

pD = 36.4 and DIC = 2446.7

DIC is an estimate of expected predictive error (lower deviance is better).

- - 1. Analysis following multiple imputation of missing Xpert Ultra and culture test results for ONLY the eligible participants with unconfirmed TB status (Results based on the 41^st^ imputed dataset randomly chosen from among the model results of the 100 imputed datasets)

Inference for Bugs model at "model1_MICE_lca.txt", fit using jags,

3 chains, each with 50000 iterations (first 25000 discarded), n.thin = 10 n.sims = 7500 iterations saved

mu.vect sd.vect 50% 2.5% 97.5% Rhat n.eff

a_prev[1] -2.214 0.090 -2.224 -2.359 -2.015 1.002 1900

a_prev[2] -0.076 0.151 -0.075 -0.380 0.216 1.001 7500

a_prev[3] -1.701 0.483 -1.635 -2.875 -0.953 1.001 7500

alpha[1,1] -0.956 0.021 -0.956 -0.999 -0.914 1.001 5000

alpha[2,1] -0.123 0.047 -0.122 -0.217 -0.030 1.001 7500

alpha[3,1] -2.737 0.375 -2.685 -3.628 -2.159 1.001 3100

alpha[1,2] -0.440 0.021 -0.440 -0.481 -0.402 1.001 7000

alpha[2,2] -0.059 0.047 -0.058 -0.152 0.032 1.001 7500

alpha[3,2] -0.376 0.043 -0.376 -0.461 -0.290 1.001 4700

alpha[4,2] -3.187 0.338 -3.144 -3.969 -2.655 1.001 7500

alpha[1,3] -0.342 0.023 -0.341 -0.387 -0.297 1.001 3400

alpha[2,3] -0.011 0.045 -0.011 -0.099 0.075 1.001 6800

alpha[3,3] 0.680 0.037 0.680 0.607 0.751 1.001 7500

alpha[4,3] 0.137 0.039 0.137 0.060 0.213 1.001 7000

alpha[5,3] -0.652 0.034 -0.652 -0.718 -0.584 1.001 7500

alpha[1,4] -3.429 0.202 -3.415 -3.859 -3.076 1.001 7500

alpha[2,4] -0.541 0.714 -0.449 -2.134 0.597 1.001 7400

alpha[3,4] -0.860 0.640 -0.779 -2.356 0.156 1.001 7500

alpha[1,5] -2.990 0.148 -2.979 -3.318 -2.738 1.001 7500

alpha[2,5] 0.438 0.199 0.433 0.059 0.839 1.001 7500

alpha[3,5] -1.134 0.558 -1.060 -2.433 -0.247 1.001 7500

beta[1,1] -0.851 0.195 -0.849 -1.251 -0.471 1.001 7500

beta[2,1] 0.665 0.393 0.669 -0.106 1.419 1.001 7500

beta[3,1] -0.010 0.984 0.013 -1.961 1.900 1.001 7500

beta[1,2] 1.437 0.286 1.422 0.911 2.031 1.002 1300

beta[2,2] 0.122 0.623 0.075 -0.944 1.501 1.001 7500

beta[3,2] -0.028 1.000 -0.029 -2.019 1.912 1.001 7500

beta[1,3] 1.262 0.641 1.234 0.087 2.603 1.001 7500

beta[2,3] 0.843 0.667 0.857 -0.539 2.104 1.001 7500

beta[3,3] 0.490 0.791 0.444 -0.953 2.125 1.001 7500

beta[4,3] -0.004 1.000 -0.003 -1.928 1.969 1.001 7500

beta[1,4] 0.204 0.280 0.218 -0.395 0.707 1.001 5100

beta[2,4] 0.392 0.591 0.341 -0.629 1.713 1.001 4400

beta[3,4] -0.022 1.002 -0.037 -1.943 1.913 1.001 7500

beta[1,5] 0.409 0.598 0.375 -0.662 1.638 1.002 2100

beta[2,5] -0.076 0.608 -0.046 -1.319 1.022 1.002 1800

beta[3,5] -0.399 0.399 -0.405 -1.190 0.386 1.001 6300

beta[4,5] -0.012 1.012 -0.020 -2.015 1.963 1.001 7500

pi[1] 0.014 0.004 0.013 0.009 0.022 1.002 1900

pi[2] 0.012 0.005 0.011 0.005 0.024 1.002 1800

pi[3] 0.000 0.000 0.000 0.000 0.001 1.001 7500

pi[4] 0.009 0.002 0.008 0.006 0.014 1.002 1300

pv[1] 0.502 0.005 0.502 0.492 0.512 1.001 7500

pv[2] 0.144 0.004 0.144 0.137 0.151 1.001 4700

pv[3] 0.355 0.005 0.354 0.345 0.364 1.001 7500

se[1,1] 0.202 0.054 0.198 0.105 0.319 1.001 7500

se[2,1] 0.917 0.041 0.922 0.819 0.979 1.003 1200

se[3,1] 0.966 0.024 0.971 0.907 0.996 1.001 7500

se[4,1] 0.579 0.106 0.586 0.347 0.760 1.001 4600

se[5,1] 0.617 0.104 0.628 0.380 0.789 1.001 3700

se[1,2] 0.430 0.132 0.427 0.184 0.694 1.001 7500

se[2,2] 0.913 0.075 0.932 0.720 0.998 1.001 6100

se[3,2] 0.974 0.040 0.990 0.857 1.000 1.001 7500

se[4,2] 0.691 0.184 0.712 0.304 0.976 1.002 2100

se[5,2] 0.468 0.149 0.468 0.187 0.752 1.001 3400

se[1,3] 0.272 0.248 0.198 0.002 0.866 1.001 7500

se[2,3] 0.835 0.202 0.922 0.266 1.000 1.001 7500

se[3,3] 0.903 0.150 0.967 0.441 1.000 1.002 5700

se[4,3] 0.549 0.295 0.571 0.034 0.987 1.001 7500

se[5,3] 0.585 0.291 0.620 0.042 0.990 1.001 7500

se[1,4] 0.247 0.055 0.244 0.148 0.364 1.001 7500

se[2,4] 0.915 0.039 0.920 0.825 0.975 1.003 1100

se[3,4] 0.967 0.023 0.972 0.909 0.996 1.001 7500

se[4,4] 0.595 0.107 0.606 0.358 0.775 1.001 2900

se[5,4] 0.585 0.101 0.596 0.357 0.750 1.002 2800

sp[1,1] 0.831 0.005 0.831 0.820 0.841 1.001 5100

sp[2,1] 0.674 0.007 0.673 0.661 0.687 1.001 5900

sp[3,1] 0.548 0.007 0.548 0.533 0.562 1.001 5800

sp[4,1] 1.000 0.000 1.000 0.999 1.000 1.001 7500

sp[5,1] 0.998 0.001 0.999 0.997 1.000 1.001 7500

sp[1,2] 0.860 0.009 0.860 0.841 0.877 1.001 7500

sp[2,2] 0.795 0.011 0.795 0.772 0.816 1.001 7500

sp[3,2] 0.528 0.013 0.528 0.501 0.554 1.001 4700

sp[4,2] 1.000 0.001 1.000 0.998 1.000 1.003 5200

sp[5,2] 0.994 0.002 0.995 0.989 0.998 1.001 7500

sp[1,3] 1.000 0.000 1.000 0.999 1.000 1.001 7500

sp[2,3] 1.000 0.000 1.000 0.999 1.000 1.001 7500

sp[3,3] 0.840 0.006 0.840 0.827 0.851 1.001 7500

sp[4,3] 1.000 0.000 1.000 1.000 1.000 1.003 6600

sp[5,3] 1.000 0.000 1.000 1.000 1.000 1.001 7500

sp[1,4] 0.895 0.003 0.895 0.889 0.901 1.001 7000

sp[2,4] 0.808 0.004 0.807 0.800 0.816 1.001 7500

sp[3,4] 0.649 0.005 0.649 0.640 0.659 1.001 5200

sp[4,4] 1.000 0.000 1.000 0.999 1.000 1.001 7500

sp[5,4] 0.998 0.000 0.998 0.997 0.999 1.001 7500

deviance 2380.553 7.735 2379.987 2367.032 2397.497 1.001 3200

For each parameter, n.eff is a crude measure of effective sample size,

and Rhat is the potential scale reduction factor (at convergence, Rhat=1).

DIC info (using the rule, pD = var(deviance)/2)

pD = 29.9 and DIC = 2410.5

DIC is an estimate of expected predictive error (lower deviance is better).

- - 1. Analysis with simultaneous imputation of missing Xpert Ultra and culture test results assuming the data is MAR

Inference for Bugs model at "vb_model1_MAR_3_groups.txt", fit using jags, 3 chains, each with 50000 iterations (first 25000 discarded), n.thin = 10 n.sims = 7500 iterations saved

mu.vect sd.vect 50% 2.5% 97.5% Rhat n.eff

a_prev[1] -2.231 0.080 -2.237 -2.369 -2.060 1.004 7500

a_prev[2] 0.128 0.337 0.136 -0.561 0.756 1.006 440

a_prev[3] -1.265 0.244 -1.260 -1.759 -0.792 1.001 6300

alpha[1,1,1] -0.955 0.022 -0.955 -0.998 -0.914 1.001 7500

alpha[1,2,1] -0.429 0.021 -0.429 -0.470 -0.389 1.001 7500

alpha[2,2,1] -0.109 0.051 -0.109 -0.208 -0.011 1.001 7500

alpha[1,3,1] -0.364 0.024 -0.364 -0.412 -0.317 1.001 7500

alpha[2,3,1] 0.046 0.050 0.046 -0.052 0.146 1.001 7500

alpha[3,3,1] 0.722 0.040 0.722 0.643 0.800 1.001 7500

alpha[1,4,1] -3.339 0.192 -3.324 -3.743 -3.000 1.001 7500

alpha[1,5,1] -2.960 0.146 -2.947 -3.290 -2.716 1.001 7500

alpha[1,1,2] -1.110 0.074 -1.099 -1.279 -0.995 1.005 1200

alpha[1,2,2] -0.844 0.050 -0.841 -0.955 -0.755 1.002 1300

alpha[2,2,2] 0.090 0.279 0.145 -0.607 0.428 1.023 450

alpha[1,3,2] -0.137 0.042 -0.138 -0.219 -0.055 1.001 7500

alpha[2,3,2] -0.249 0.165 -0.236 -0.567 0.018 1.013 400

alpha[3,3,2] 0.523 0.124 0.522 0.297 0.761 1.015 420

alpha[1,4,2] -3.339 0.192 -3.324 -3.743 -3.000 1.001 7500

alpha[1,5,2] -2.960 0.146 -2.947 -3.290 -2.716 1.001 7500

alpha[1,1,3] -3.586 0.328 -3.538 -4.370 -3.074 1.001 7500

alpha[1,2,3] -3.579 0.319 -3.538 -4.333 -3.069 1.001 7500

alpha[2,2,3] -0.001 0.996 -0.003 -1.948 1.969 1.001 7500

alpha[1,3,3] -0.993 0.026 -0.993 -1.043 -0.943 1.001 7500

alpha[2,3,3] 0.016 0.985 0.016 -1.879 1.946 1.001 7500

alpha[3,3,3] -0.006 1.006 -0.007 -1.960 1.961 1.001 4100

alpha[1,4,3] -3.339 0.192 -3.324 -3.743 -3.000 1.001 7500

alpha[1,5,3] -2.960 0.146 -2.947 -3.290 -2.716 1.001 7500

beta[1,1,1] -0.887 0.200 -0.883 -1.282 -0.511 1.001 7500

beta[1,2,1] 1.449 0.291 1.434 0.933 2.067 1.001 7500

beta[1,3,1] 1.196 0.639 1.166 0.000 2.518 1.001 3100

beta[2,3,1] 0.844 0.668 0.852 -0.497 2.134 1.002 2600

beta[1,4,1] 0.245 0.261 0.256 -0.283 0.725 1.001 7500

beta[1,5,1] 0.519 0.581 0.501 -0.551 1.695 1.001 7500

beta[2,5,1] -0.166 0.592 -0.144 -1.370 0.912 1.001 7500

beta[1,1,2] 0.142 1.023 0.199 -1.946 2.010 1.001 3800

beta[1,2,2] 0.240 0.998 0.315 -1.835 2.103 1.002 2700

beta[1,3,2] 0.192 1.014 0.199 -1.808 2.158 1.002 1800

beta[2,3,2] 0.384 0.985 0.417 -1.614 2.280 1.002 1900

beta[1,4,2] 0.245 0.261 0.256 -0.283 0.725 1.001 7500

beta[1,5,2] 0.519 0.581 0.501 -0.551 1.695 1.001 7500

beta[2,5,2] -0.166 0.592 -0.144 -1.370 0.912 1.001 7500

beta[1,1,3] -0.186 1.017 -0.186 -2.141 1.863 1.001 7500

beta[1,2,3] -0.175 1.002 -0.205 -2.086 1.840 1.001 7500

beta[1,3,3] -0.023 1.019 -0.030 -2.048 1.950 1.001 7500

beta[2,3,3] -0.022 0.991 -0.009 -1.966 1.903 1.001 7500

beta[1,4,3] 0.245 0.261 0.256 -0.283 0.725 1.001 7500

beta[1,5,3] 0.519 0.581 0.501 -0.551 1.695 1.001 7500

beta[2,5,3] -0.166 0.592 -0.144 -1.370 0.912 1.001 7500

pi[1] 0.013 0.003 0.013 0.009 0.020 1.003 7500

pi[2] 0.023 0.019 0.018 0.003 0.073 1.005 540

pi[3] 0.000 0.000 0.000 0.000 0.001 1.001 7500

pi[4] 0.010 0.003 0.009 0.006 0.019 1.007 480

pv[1] 0.502 0.005 0.502 0.492 0.512 1.001 7500

pv[2] 0.144 0.004 0.144 0.137 0.151 1.001 7500

pv[3] 0.355 0.005 0.355 0.345 0.364 1.001 7500

se[1,1] 0.192 0.053 0.189 0.100 0.305 1.001 7500

se[2,1] 0.918 0.041 0.924 0.825 0.981 1.001 7500

se[3,1] 0.961 0.027 0.967 0.893 0.995 1.001 7500

se[4,1] 0.594 0.098 0.601 0.389 0.766 1.002 7500

se[5,1] 0.639 0.098 0.651 0.417 0.797 1.003 7500

se[1,2] 0.545 0.292 0.579 0.026 0.978 1.001 3900

se[2,2] 0.575 0.286 0.624 0.033 0.982 1.004 1500

se[3,2] 0.616 0.294 0.693 0.033 0.991 1.002 1800

se[4,2] 0.594 0.098 0.601 0.389 0.766 1.002 7500

se[5,2] 0.639 0.098 0.651 0.417 0.797 1.003 7500

se[1,3] 0.447 0.289 0.426 0.016 0.969 1.001 7500

se[2,3] 0.447 0.287 0.419 0.018 0.967 1.001 7500

se[3,3] 0.491 0.297 0.485 0.018 0.978 1.001 7500

se[4,3] 0.594 0.098 0.601 0.389 0.766 1.002 7500

se[5,3] 0.639 0.098 0.651 0.417 0.797 1.003 7500

se[1,4] 0.304 0.117 0.291 0.119 0.555 1.001 3000

se[2,4] 0.814 0.107 0.838 0.536 0.951 1.005 1800

se[3,4] 0.857 0.108 0.886 0.554 0.978 1.015 410

se[4,4] 0.594 0.098 0.601 0.389 0.766 1.002 7500

se[5,4] 0.639 0.098 0.651 0.417 0.797 1.003 7500

sp[1,1] 0.830 0.005 0.830 0.820 0.841 1.001 7500

sp[2,1] 0.673 0.007 0.673 0.659 0.686 1.001 7500

sp[3,1] 0.547 0.007 0.547 0.533 0.561 1.001 7500

sp[4,1] 0.999 0.000 1.000 0.999 1.000 1.001 6000

sp[5,1] 0.998 0.001 0.998 0.997 0.999 1.001 7500

sp[1,2] 0.866 0.015 0.864 0.840 0.900 1.004 1300

sp[2,2] 0.795 0.016 0.794 0.768 0.829 1.004 600

sp[3,2] 0.525 0.017 0.525 0.493 0.559 1.003 1100

sp[4,2] 0.999 0.000 1.000 0.999 1.000 1.001 6000

sp[5,2] 0.998 0.001 0.998 0.997 0.999 1.001 7500

sp[1,3] 1.000 0.000 1.000 0.999 1.000 1.001 7500

sp[2,3] 1.000 0.000 1.000 0.999 1.000 1.001 7500

sp[3,3] 0.839 0.006 0.839 0.827 0.852 1.001 7500

sp[4,3] 0.999 0.000 1.000 0.999 1.000 1.001 6000

sp[5,3] 0.998 0.001 0.998 0.997 0.999 1.001 7500

sp[1,4] 0.896 0.004 0.896 0.889 0.903 1.001 4500

sp[2,4] 0.807 0.004 0.807 0.798 0.816 1.002 1500

sp[3,4] 0.649 0.005 0.648 0.639 0.659 1.002 1300

sp[4,4] 0.999 0.000 1.000 0.999 1.000 1.001 6000

sp[5,4] 0.998 0.001 0.998 0.997 0.999 1.001 7500

taup[1] 2.069 1.044 1.974 0.380 4.247 1.004 720

taup[2] 0.036 0.038 0.025 0.003 0.134 1.002 1500

deviance 24880.650 108.097 24908.406 24609.595 25024.607 1.008 430

For each parameter, n.eff is a crude measure of effective sample size,

and Rhat is the potential scale reduction factor (at convergence, Rhat=1).

DIC info (using the rule, pD = var(deviance)/2)

pD = 5817.1 and DIC = 30697.7

DIC is an estimate of expected predictive error (lower deviance is better).

- - 1. Analysis with simultaneous imputation of missing Xpert Ultra and culture test results assuming the data is MNAR

Inference for Bugs model at "vb_model1_MNAR_3_groups.txt", fit using jags, 3 chains, each with 50000 iterations (first 25000 discarded), n.thin = 10 n.sims = 7500 iterations saved

mu.vect sd.vect 50% 2.5% 97.5% Rhat n.eff

a_prev -2.248 0.074 -2.252 -2.386 -2.092 1.001 4500

alpha[1,1,1] -0.955 0.021 -0.955 -0.997 -0.913 1.001 4000

alpha[1,2,1] -0.429 0.021 -0.429 -0.470 -0.388 1.001 7500

alpha[2,2,1] -0.108 0.051 -0.108 -0.209 -0.007 1.001 7500

alpha[1,3,1] -0.365 0.024 -0.365 -0.413 -0.318 1.001 5200

alpha[2,3,1] 0.046 0.050 0.047 -0.051 0.145 1.001 6500

alpha[3,3,1] 0.723 0.039 0.723 0.648 0.800 1.001 7300

alpha[1,4,1] -3.339 0.195 -3.327 -3.751 -2.995 1.001 3600

alpha[1,5,1] -2.953 0.143 -2.942 -3.266 -2.706 1.001 3700

alpha[1,1,2] -1.072 0.043 -1.072 -1.159 -0.989 1.001 7500

alpha[1,2,2] -0.831 0.042 -0.830 -0.914 -0.751 1.001 7500

alpha[2,2,2] 0.196 0.112 0.198 -0.028 0.410 1.001 7500

alpha[1,3,2] -0.140 0.040 -0.141 -0.218 -0.062 1.001 7500

alpha[2,3,2] -0.198 0.101 -0.197 -0.396 0.000 1.001 7500

alpha[3,3,2] 0.542 0.087 0.543 0.372 0.716 1.001 7500

alpha[1,4,2] -3.339 0.195 -3.327 -3.751 -2.995 1.001 3600

alpha[1,5,2] -2.953 0.143 -2.942 -3.266 -2.706 1.001 3700

alpha[1,1,3] -3.580 0.324 -3.537 -4.333 -3.071 1.001 7500

alpha[1,2,3] -3.585 0.323 -3.551 -4.319 -3.071 1.001 5300

alpha[2,2,3] 0.016 0.987 0.016 -1.877 1.931 1.001 3500

alpha[1,3,3] -0.994 0.026 -0.994 -1.043 -0.943 1.001 7500

alpha[2,3,3] 0.019 0.998 0.006 -1.937 1.987 1.001 7500

alpha[3,3,3] -0.004 1.004 -0.010 -1.967 1.964 1.001 7500

alpha[1,4,3] -3.339 0.195 -3.327 -3.751 -2.995 1.001 3600

alpha[1,5,3] -2.953 0.143 -2.942 -3.266 -2.706 1.001 3700

b_psi[1,1] 0.005 1.008 0.004 -1.963 2.012 1.001 7500

b_psi[2,1] -0.005 1.006 -0.012 -1.953 1.996 1.001 7500

b_psi[1,2] 0.017 0.991 0.023 -1.900 1.966 1.001 7500

b_psi[2,2] -0.017 1.005 -0.018 -1.956 1.976 1.001 5200

b_psi[1,3] 0.001 0.996 0.014 -1.922 1.969 1.001 7500

b_psi[2,3] -0.020 1.011 -0.006 -1.995 1.974 1.001 7500

b_psi[1,4] 0.002 1.009 0.009 -1.956 2.019 1.001 4200

b_psi[2,4] -0.001 1.007 -0.004 -1.970 1.955 1.001 3600

b_tau[1] 0.007 0.317 0.003 -0.621 0.631 1.001 6200

b_tau[2] -1.317 0.304 -1.324 -1.890 -0.708 1.001 7500

beta[1,1,1] -0.888 0.199 -0.883 -1.289 -0.512 1.001 7500

beta[1,2,1] 1.450 0.294 1.433 0.926 2.083 1.001 7500

beta[1,3,1] 1.193 0.651 1.167 -0.005 2.535 1.001 7500

beta[2,3,1] 0.852 0.672 0.868 -0.483 2.149 1.001 6100

beta[1,4,1] 0.285 0.248 0.295 -0.220 0.744 1.001 7000

beta[1,5,1] 0.607 0.579 0.575 -0.416 1.850 1.001 7500

beta[2,5,1] -0.250 0.594 -0.222 -1.467 0.818 1.001 7500

beta[1,1,2] 0.014 1.017 0.009 -1.953 2.018 1.001 7500

beta[1,2,2] 0.082 1.019 0.085 -1.913 2.011 1.001 7500

beta[1,3,2] 0.097 1.012 0.113 -1.949 2.027 1.001 7500

beta[2,3,2] 0.160 1.008 0.177 -1.858 2.083 1.001 7500

beta[1,4,2] 0.285 0.248 0.295 -0.220 0.744 1.001 7000

beta[1,5,2] 0.607 0.579 0.575 -0.416 1.850 1.001 7500

beta[2,5,2] -0.250 0.594 -0.222 -1.467 0.818 1.001 7500

beta[1,1,3] -0.738 0.954 -0.817 -2.523 1.336 1.001 7500

beta[1,2,3] -0.730 0.968 -0.801 -2.470 1.380 1.001 7500

beta[1,3,3] -0.008 0.996 -0.005 -1.901 1.929 1.001 7500

beta[2,3,3] -0.025 0.993 -0.030 -2.030 1.897 1.001 7500

beta[1,4,3] 0.285 0.248 0.295 -0.220 0.744 1.001 7000

beta[1,5,3] 0.607 0.579 0.575 -0.416 1.850 1.001 7500

beta[2,5,3] -0.250 0.594 -0.222 -1.467 0.818 1.001 7500

pi[1] 0.012 0.002 0.012 0.009 0.018 1.001 4400

pi[2] 0.006 0.002 0.006 0.003 0.011 1.001 3800

pi[3] 0.001 0.001 0.001 0.000 0.003 1.001 7500

pi[4] 0.008 0.002 0.007 0.005 0.011 1.001 4700

psi[1,1] 0.501 0.289 0.501 0.025 0.978 1.001 7500

psi[2,1] 0.498 0.290 0.495 0.025 0.977 1.001 7500

psi[1,2] 0.505 0.287 0.509 0.029 0.975 1.001 7500

psi[2,2] 0.495 0.290 0.493 0.025 0.976 1.001 6100

psi[1,3] 0.500 0.288 0.505 0.027 0.976 1.001 7500

psi[2,3] 0.494 0.291 0.497 0.023 0.976 1.001 7500

psi[1,4] 0.500 0.290 0.503 0.025 0.978 1.001 4000

psi[2,4] 0.500 0.289 0.498 0.024 0.975 1.001 3900

pv[1] 0.502 0.005 0.502 0.492 0.511 1.001 7500

pv[2] 0.144 0.004 0.144 0.137 0.151 1.001 7500

pv[3] 0.355 0.005 0.355 0.345 0.364 1.001 7500

se[1,1] 0.192 0.053 0.188 0.099 0.304 1.001 7500

se[2,1] 0.918 0.041 0.924 0.823 0.981 1.001 7500

se[3,1] 0.961 0.026 0.967 0.897 0.995 1.001 7500

se[4,1] 0.609 0.093 0.616 0.413 0.772 1.001 7500

se[5,1] 0.652 0.089 0.659 0.454 0.805 1.001 3700

se[1,2] 0.503 0.292 0.504 0.025 0.978 1.001 7500

se[2,2] 0.524 0.292 0.534 0.028 0.978 1.001 7500

se[3,2] 0.554 0.301 0.585 0.024 0.988 1.001 7500

se[4,2] 0.609 0.093 0.616 0.413 0.772 1.001 7500

se[5,2] 0.652 0.089 0.659 0.454 0.805 1.001 3700

se[1,3] 0.290 0.255 0.207 0.006 0.909 1.001 7500

se[2,3] 0.293 0.258 0.212 0.007 0.916 1.001 7500

se[3,3] 0.495 0.289 0.495 0.028 0.973 1.001 7500

se[4,3] 0.609 0.093 0.616 0.413 0.772 1.001 7500

se[5,3] 0.652 0.089 0.659 0.454 0.805 1.001 3700

se[1,4] 0.232 0.058 0.230 0.127 0.346 1.001 7500

se[2,4] 0.832 0.058 0.836 0.711 0.934 1.001 7500

se[3,4] 0.886 0.049 0.890 0.785 0.968 1.001 7500

se[4,4] 0.609 0.093 0.616 0.413 0.772 1.001 7500

se[5,4] 0.652 0.089 0.659 0.454 0.805 1.001 3700

sp[1,1] 0.830 0.005 0.830 0.819 0.841 1.001 4000

sp[2,1] 0.673 0.007 0.672 0.659 0.686 1.001 7500

sp[3,1] 0.547 0.007 0.547 0.533 0.562 1.001 7500

sp[4,1] 0.999 0.000 1.000 0.999 1.000 1.001 3100

sp[5,1] 0.998 0.001 0.998 0.997 0.999 1.001 7500

sp[1,2] 0.858 0.010 0.858 0.839 0.877 1.001 7500

sp[2,2] 0.788 0.011 0.788 0.766 0.810 1.001 7500

sp[3,2] 0.522 0.013 0.522 0.496 0.548 1.001 7500

sp[4,2] 0.999 0.000 1.000 0.999 1.000 1.001 3100

sp[5,2] 0.998 0.001 0.998 0.997 0.999 1.001 7500

sp[1,3] 1.000 0.000 1.000 0.999 1.000 1.001 7500

sp[2,3] 1.000 0.000 1.000 0.999 1.000 1.001 4000

sp[3,3] 0.840 0.006 0.840 0.827 0.851 1.001 7500

sp[4,3] 0.999 0.000 1.000 0.999 1.000 1.001 3100

sp[5,3] 0.998 0.001 0.998 0.997 0.999 1.001 7500

sp[1,4] 0.895 0.003 0.895 0.888 0.901 1.001 7200

sp[2,4] 0.806 0.004 0.806 0.798 0.814 1.001 7500

sp[3,4] 0.648 0.005 0.648 0.638 0.657 1.001 7500

sp[4,4] 0.999 0.000 1.000 0.999 1.000 1.001 3100

sp[5,4] 0.998 0.001 0.998 0.997 0.999 1.001 7500

tau[1] 0.503 0.121 0.501 0.267 0.736 1.001 5300

tau[2] 0.104 0.055 0.093 0.029 0.239 1.001 7500

taup[1] 0.819 0.176 0.822 0.463 1.149 1.001 4800

taup[2] 0.167 0.082 0.153 0.050 0.364 1.001 7500

deviance 24959.278 40.216 24959.392 24879.141 25040.068 1.001 4800

For each parameter, n.eff is a crude measure of effective sample size,

and Rhat is the potential scale reduction factor (at convergence, Rhat=1).

DIC info (using the rule, pD = var(deviance)/2)

pD = 808.5 and DIC = 25767.8

DIC is an estimate of expected predictive error (lower deviance is better).

1. Model results for the Vukuzazi data based on Bayesian LCA (Table 6 in the main document)
2. Analysis with simultaneous imputation of missing Xpert Ultra and culture test results assuming the data is MAR (Model adjusted for age, sex and HIV status)

Inference for Bugs model at "vb_model1_MAR_3_groups_adj.txt", fit using jags,3 chains, each with 50000 iterations (first 25000 discarded), n.thin = 10 n.sims = 7500 iterations saved

mu.vect sd.vect 50% 2.5% 97.5% Rhat n.eff

a_prev[1] -2.689 0.124 -2.687 -2.940 -2.452 1.001 7500

a_prev[2] -0.598 0.201 -0.591 -1.009 -0.226 1.001 7000

a_prev[3] -1.270 0.235 -1.259 -1.759 -0.838 1.001 3600

a_prev[4] 0.291 0.122 0.289 0.059 0.538 1.001 7500

a_prev[5] 0.374 0.112 0.372 0.154 0.593 1.001 7500

a_prev[6] 0.141 0.142 0.142 -0.139 0.417 1.001 7500

a_prev[7] 0.262 0.134 0.260 -0.004 0.524 1.001 7500

a_prev[8] 0.146 0.164 0.149 -0.187 0.453 1.001 7500

alpha[1,1,1] -0.874 0.051 -0.875 -0.975 -0.773 1.001 7500

alpha[2,1,1] -0.033 0.052 -0.033 -0.135 0.068 1.001 7500

alpha[3,1,1] -0.040 0.046 -0.039 -0.131 0.051 1.001 7500

alpha[4,1,1] -0.074 0.064 -0.074 -0.199 0.050 1.001 4200

alpha[5,1,1] -0.085 0.058 -0.085 -0.200 0.027 1.001 7500

alpha[6,1,1] -0.063 0.070 -0.064 -0.198 0.074 1.001 4100

alpha[1,2,1] -1.282 0.054 -1.282 -1.390 -1.176 1.001 3600

alpha[2,2,1] -0.106 0.052 -0.106 -0.206 -0.004 1.001 6300

alpha[3,2,1] 0.292 0.045 0.292 0.201 0.381 1.001 7500

alpha[4,2,1] 0.217 0.042 0.217 0.132 0.297 1.001 7100

alpha[5,2,1] 0.626 0.064 0.626 0.501 0.750 1.001 7500

alpha[6,2,1] 0.895 0.058 0.895 0.784 1.011 1.001 3300

alpha[7,2,1] 1.087 0.067 1.087 0.958 1.218 1.001 3800

alpha[1,3,1] -0.458 0.046 -0.459 -0.550 -0.367 1.001 7500

alpha[2,3,1] 0.059 0.052 0.059 -0.043 0.159 1.001 7100

alpha[3,3,1] 0.844 0.043 0.845 0.760 0.926 1.001 7500

alpha[4,3,1] 0.158 0.045 0.158 0.070 0.247 1.002 1900

alpha[5,3,1] 0.760 0.041 0.760 0.680 0.840 1.001 7500

alpha[6,3,1] -0.286 0.057 -0.287 -0.399 -0.174 1.001 7500

alpha[7,3,1] -0.439 0.053 -0.439 -0.544 -0.334 1.001 7500

alpha[8,3,1] -0.324 0.064 -0.325 -0.451 -0.199 1.001 7500

alpha[1,4,1] -3.514 0.172 -3.500 -3.879 -3.216 1.001 7500

alpha[1,5,1] -2.916 0.135 -2.908 -3.202 -2.680 1.001 7500

alpha[1,1,2] -0.989 0.088 -0.988 -1.162 -0.819 1.001 3400

alpha[2,1,2] -0.187 0.111 -0.185 -0.405 0.026 1.001 7500

alpha[3,1,2] -0.139 0.092 -0.138 -0.324 0.039 1.002 2600

alpha[4,1,2] 0.021 0.123 0.021 -0.221 0.261 1.002 2300

alpha[5,1,2] -0.041 0.109 -0.043 -0.256 0.175 1.001 3100

alpha[6,1,2] 0.145 0.130 0.147 -0.107 0.403 1.001 6300

alpha[1,2,2] -1.413 0.094 -1.411 -1.599 -1.234 1.001 7500

alpha[2,2,2] 0.200 0.107 0.201 -0.012 0.409 1.001 7500

alpha[3,2,2] 0.332 0.096 0.331 0.144 0.523 1.002 3000

alpha[4,2,2] 0.019 0.085 0.017 -0.149 0.185 1.001 7500

alpha[5,2,2] 0.526 0.118 0.525 0.299 0.759 1.001 3600

alpha[6,2,2] 0.639 0.106 0.639 0.437 0.852 1.001 3000

alpha[7,2,2] 0.941 0.122 0.939 0.705 1.185 1.001 7500

alpha[1,3,2] -0.124 0.075 -0.124 -0.271 0.022 1.001 7500

alpha[2,3,2] -0.174 0.099 -0.174 -0.366 0.018 1.001 7500

alpha[3,3,2] 0.764 0.089 0.762 0.590 0.938 1.001 7500

alpha[4,3,2] 0.128 0.090 0.127 -0.046 0.303 1.001 6500

alpha[5,3,2] 0.711 0.076 0.711 0.563 0.859 1.001 7500

alpha[6,3,2] -0.400 0.102 -0.401 -0.602 -0.199 1.001 7500

alpha[7,3,2] -0.805 0.095 -0.805 -0.986 -0.616 1.001 7500

alpha[8,3,2] -0.394 0.113 -0.394 -0.613 -0.173 1.001 7500

alpha[1,4,2] -3.514 0.172 -3.500 -3.879 -3.216 1.001 7500

alpha[1,5,2] -2.916 0.135 -2.908 -3.202 -2.680 1.001 7500

alpha[1,1,3] -3.520 0.405 -3.478 -4.426 -2.862 1.001 7500

alpha[2,1,3] -0.596 0.750 -0.534 -2.251 0.691 1.001 7500

alpha[3,1,3] -0.596 0.729 -0.528 -2.203 0.654 1.001 5300

alpha[4,1,3] -0.580 0.746 -0.512 -2.165 0.713 1.001 7500

alpha[5,1,3] -0.479 0.758 -0.430 -2.135 0.822 1.001 7500

alpha[6,1,3] -0.211 0.857 -0.173 -1.995 1.328 1.001 7500

alpha[1,2,3] -3.505 0.409 -3.461 -4.416 -2.826 1.001 7500

alpha[2,2,3] 0.009 0.999 0.015 -1.915 1.966 1.001 7500

alpha[3,2,3] -0.594 0.738 -0.517 -2.211 0.671 1.001 7500

alpha[4,2,3] -0.608 0.727 -0.550 -2.192 0.651 1.001 7500

alpha[5,2,3] -0.595 0.732 -0.528 -2.160 0.709 1.001 7500

alpha[6,2,3] -0.496 0.750 -0.437 -2.125 0.804 1.001 7500

alpha[7,2,3] -0.229 0.869 -0.191 -2.040 1.300 1.001 7500

alpha[1,3,3] -0.963 0.043 -0.963 -1.047 -0.880 1.001 7500

alpha[2,3,3] 0.004 0.999 0.010 -1.908 1.923 1.001 3500

alpha[3,3,3] 0.000 1.016 -0.002 -1.975 2.011 1.001 6200

alpha[4,3,3] -0.038 0.064 -0.037 -0.165 0.088 1.001 7500

alpha[5,3,3] 0.594 0.057 0.595 0.480 0.709 1.001 6800

alpha[6,3,3] -0.392 0.063 -0.391 -0.517 -0.269 1.001 7500

alpha[7,3,3] -0.916 0.113 -0.914 -1.141 -0.701 1.001 7100

alpha[8,3,3] -0.994 0.297 -0.983 -1.617 -0.449 1.001 7500

alpha[1,4,3] -3.514 0.172 -3.500 -3.879 -3.216 1.001 7500

alpha[1,5,3] -2.916 0.135 -2.908 -3.202 -2.680 1.001 7500

beta[1,1,1] -1.176 0.303 -1.173 -1.773 -0.591 1.001 7500

beta[2,1,1] 0.234 0.310 0.235 -0.377 0.836 1.001 7300

beta[3,1,1] 0.038 0.306 0.040 -0.562 0.626 1.001 4000

beta[4,1,1] -0.087 0.335 -0.080 -0.750 0.559 1.001 7500

beta[5,1,1] 0.279 0.313 0.280 -0.336 0.895 1.001 6100

beta[6,1,1] 0.119 0.392 0.125 -0.668 0.848 1.001 7500

beta[1,2,1] 1.316 0.322 1.310 0.701 1.958 1.001 5800

beta[2,2,1] 0.300 0.371 0.295 -0.410 1.055 1.001 4200

beta[3,2,1] 0.016 0.344 0.013 -0.659 0.694 1.002 2400

beta[4,2,1] -0.040 0.363 -0.046 -0.744 0.676 1.001 2900

beta[5,2,1] 0.241 0.381 0.228 -0.480 1.008 1.001 7500

beta[6,2,1] 0.214 0.398 0.210 -0.550 1.012 1.001 5600

beta[1,3,1] 1.381 0.377 1.382 0.647 2.139 1.001 7500

beta[2,3,1] 0.392 0.372 0.392 -0.352 1.111 1.002 2000

beta[3,3,1] -0.009 0.372 -0.011 -0.730 0.728 1.001 7500

beta[4,3,1] 0.326 0.381 0.324 -0.394 1.086 1.002 2100

beta[5,3,1] -0.090 0.375 -0.091 -0.822 0.659 1.001 7500

beta[6,3,1] 0.211 0.398 0.209 -0.562 0.991 1.001 7500

beta[7,3,1] 0.109 0.420 0.110 -0.703 0.937 1.001 7500

beta[1,4,1] 0.290 0.301 0.286 -0.283 0.904 1.001 7500

beta[2,4,1] -0.298 0.319 -0.298 -0.916 0.332 1.001 7500

beta[3,4,1] 0.220 0.319 0.221 -0.410 0.852 1.001 7500

beta[4,4,1] 0.148 0.337 0.150 -0.510 0.815 1.001 7500

beta[5,4,1] 0.046 0.333 0.051 -0.602 0.691 1.001 7500

beta[6,4,1] -0.132 0.364 -0.140 -0.835 0.603 1.001 7500

beta[1,5,1] 0.677 0.347 0.680 -0.004 1.364 1.001 5100

beta[2,5,1] -0.033 0.353 -0.034 -0.718 0.651 1.001 7500

beta[3,5,1] -0.250 0.309 -0.249 -0.858 0.359 1.001 3600

beta[4,5,1] -0.178 0.314 -0.180 -0.802 0.436 1.001 7200

beta[5,5,1] -0.081 0.330 -0.083 -0.728 0.571 1.001 7500

beta[6,5,1] -0.168 0.317 -0.167 -0.782 0.456 1.001 4800

beta[7,5,1] 0.348 0.391 0.346 -0.410 1.126 1.001 7500

beta[1,1,2] -0.980 0.454 -0.980 -1.877 -0.079 1.001 4900

beta[2,1,2] -0.001 0.450 -0.006 -0.891 0.865 1.001 6000

beta[3,1,2] 0.015 0.449 0.005 -0.852 0.908 1.001 7500

beta[4,1,2] -0.001 0.447 -0.001 -0.855 0.869 1.001 7500

beta[5,1,2] 0.028 0.459 0.031 -0.869 0.916 1.001 7500

beta[6,1,2] -0.007 0.445 -0.007 -0.876 0.857 1.001 7500

beta[1,2,2] 1.017 0.450 1.021 0.109 1.883 1.001 4900

beta[2,2,2] 0.007 0.443 0.011 -0.869 0.874 1.001 7500

beta[3,2,2] 0.009 0.447 0.012 -0.860 0.880 1.001 4800

beta[4,2,2] 0.006 0.446 0.006 -0.862 0.885 1.001 2900

beta[5,2,2] 0.012 0.446 0.015 -0.857 0.878 1.001 7500

beta[6,2,2] -0.005 0.448 0.002 -0.882 0.864 1.001 7500

beta[1,3,2] 0.998 0.447 0.998 0.110 1.854 1.001 4800

beta[2,3,2] -0.002 0.449 0.002 -0.879 0.887 1.001 7500

beta[3,3,2] -0.002 0.448 -0.002 -0.863 0.876 1.001 7500

beta[4,3,2] -0.008 0.452 -0.015 -0.875 0.885 1.002 2100

beta[5,3,2] -0.008 0.447 -0.004 -0.888 0.844 1.001 7500

beta[6,3,2] 0.012 0.443 0.009 -0.835 0.883 1.001 7500

beta[7,3,2] -0.007 0.442 -0.009 -0.864 0.858 1.002 1500

beta[1,4,2] 0.290 0.301 0.286 -0.283 0.904 1.001 7500

beta[2,4,2] -0.298 0.319 -0.298 -0.916 0.332 1.001 7500

beta[3,4,2] 0.220 0.319 0.221 -0.410 0.852 1.001 7500

beta[4,4,2] 0.148 0.337 0.150 -0.510 0.815 1.001 7500

beta[5,4,2] 0.046 0.333 0.051 -0.602 0.691 1.001 7500

beta[6,4,2] -0.132 0.364 -0.140 -0.835 0.603 1.001 7500

beta[1,5,2] 0.677 0.347 0.680 -0.004 1.364 1.001 5100

beta[2,5,2] -0.033 0.353 -0.034 -0.718 0.651 1.001 7500

beta[3,5,2] -0.250 0.309 -0.249 -0.858 0.359 1.001 3600

beta[4,5,2] -0.178 0.314 -0.180 -0.802 0.436 1.001 7200

beta[5,5,2] -0.081 0.330 -0.083 -0.728 0.571 1.001 7500

beta[6,5,2] -0.168 0.317 -0.167 -0.782 0.456 1.001 4800

beta[7,5,2] 0.348 0.391 0.346 -0.410 1.126 1.001 7500

beta[1,1,3] -1.004 0.447 -0.999 -1.864 -0.146 1.002 2600

beta[2,1,3] -0.001 0.442 -0.002 -0.851 0.858 1.001 7500

beta[3,1,3] 0.002 0.448 0.003 -0.884 0.883 1.001 3900

beta[4,1,3] 0.000 0.452 -0.008 -0.882 0.889 1.001 7500

beta[5,1,3] -0.003 0.444 0.003 -0.866 0.863 1.001 7500

beta[6,1,3] -0.006 0.448 -0.012 -0.887 0.878 1.001 7500

beta[1,2,3] 0.991 0.446 0.990 0.135 1.868 1.001 4800

beta[2,2,3] -0.002 0.445 0.004 -0.866 0.874 1.002 1200

beta[3,2,3] -0.005 0.445 -0.007 -0.880 0.879 1.001 7500

beta[4,2,3] -0.002 0.449 -0.004 -0.872 0.861 1.001 7500

beta[5,2,3] -0.001 0.448 0.003 -0.864 0.874 1.001 7500

beta[6,2,3] -0.004 0.441 -0.003 -0.874 0.855 1.001 7500

beta[1,3,3] 0.995 0.439 0.995 0.105 1.845 1.001 7500

beta[2,3,3] 0.002 0.447 0.004 -0.885 0.859 1.001 7500

beta[3,3,3] 0.007 0.450 0.006 -0.870 0.902 1.001 7500

beta[4,3,3] 0.002 0.454 0.007 -0.903 0.892 1.001 7500

beta[5,3,3] -0.002 0.449 -0.002 -0.891 0.860 1.001 7500

beta[6,3,3] -0.003 0.448 -0.002 -0.861 0.883 1.001 7500

beta[7,3,3] -0.003 0.445 -0.001 -0.881 0.858 1.001 7500

beta[1,4,3] 0.290 0.301 0.286 -0.283 0.904 1.001 7500

beta[2,4,3] -0.298 0.319 -0.298 -0.916 0.332 1.001 7500

beta[3,4,3] 0.220 0.319 0.221 -0.410 0.852 1.001 7500

beta[4,4,3] 0.148 0.337 0.150 -0.510 0.815 1.001 7500

beta[5,4,3] 0.046 0.333 0.051 -0.602 0.691 1.001 7500

beta[6,4,3] -0.132 0.364 -0.140 -0.835 0.603 1.001 7500

beta[1,5,3] 0.677 0.347 0.680 -0.004 1.364 1.001 5100

beta[2,5,3] -0.033 0.353 -0.034 -0.718 0.651 1.001 7500

beta[3,5,3] -0.250 0.309 -0.249 -0.858 0.359 1.001 3600

beta[4,5,3] -0.178 0.314 -0.180 -0.802 0.436 1.001 7200

beta[5,5,3] -0.081 0.330 -0.083 -0.728 0.571 1.001 7500

beta[6,5,3] -0.168 0.317 -0.167 -0.782 0.456 1.001 4800

beta[7,5,3] 0.348 0.391 0.346 -0.410 1.126 1.001 7500

pi[1] 0.010 0.003 0.010 0.006 0.017 1.001 7500

pi[2] 0.005 0.001 0.005 0.003 0.007 1.001 7500

pi[3] 0.011 0.003 0.011 0.007 0.017 1.001 7500

pi[4] 0.004 0.001 0.004 0.003 0.007 1.001 7500

pi[5] 0.004 0.001 0.004 0.002 0.007 1.001 7500

pi[6] 0.006 0.002 0.006 0.003 0.011 1.001 7500

pi[7] 0.009 0.002 0.008 0.005 0.014 1.001 7500

pi[8] 0.007 0.003 0.006 0.003 0.013 1.001 7500

pi[9] 0.018 0.003 0.017 0.012 0.025 1.001 7500

pi[10] 0.001 0.001 0.001 0.000 0.003 1.001 7500

pi[11] 0.000 0.000 0.000 0.000 0.001 1.001 3900

pi[12] 0.007 0.001 0.007 0.005 0.010 1.001 7500

pv[1] 0.502 0.005 0.502 0.492 0.512 1.001 6900

pv[2] 0.144 0.003 0.144 0.137 0.150 1.001 7500

pv[3] 0.354 0.005 0.354 0.345 0.364 1.001 3500

se[1,1] 0.208 0.074 0.197 0.094 0.379 1.001 7500

se[2,1] 0.859 0.069 0.870 0.697 0.961 1.001 3700

se[3,1] 0.861 0.074 0.875 0.687 0.968 1.001 7500

se[4,1] 0.546 0.117 0.550 0.318 0.770 1.001 7500

se[5,1] 0.601 0.105 0.606 0.383 0.790 1.001 7500

se[1,2] 0.178 0.054 0.171 0.088 0.299 1.001 3800

se[2,2] 0.853 0.052 0.859 0.734 0.936 1.001 5300

se[3,2] 0.872 0.059 0.881 0.735 0.959 1.001 4000

se[4,2] 0.641 0.087 0.644 0.466 0.807 1.001 7500

se[5,2] 0.700 0.073 0.704 0.549 0.828 1.001 3300

se[1,3] 0.189 0.069 0.180 0.081 0.347 1.001 7500

se[2,3] 0.849 0.066 0.859 0.694 0.949 1.001 7500

se[3,3] 0.873 0.070 0.886 0.706 0.972 1.001 7500

se[4,3] 0.673 0.104 0.679 0.451 0.859 1.001 7500

se[5,3] 0.637 0.091 0.643 0.447 0.799 1.001 7500

se[1,4] 0.185 0.056 0.179 0.094 0.313 1.001 4400

se[2,4] 0.857 0.052 0.864 0.737 0.940 1.001 5600

se[3,4] 0.867 0.060 0.876 0.728 0.956 1.001 3800

se[4,4] 0.584 0.093 0.586 0.398 0.763 1.001 7500

se[5,4] 0.686 0.080 0.689 0.521 0.828 1.001 7500

se[1,5] 0.169 0.055 0.163 0.080 0.293 1.001 4500

se[2,5] 0.855 0.052 0.861 0.736 0.937 1.001 7500

se[3,5] 0.875 0.059 0.884 0.736 0.961 1.001 3300

se[4,5] 0.622 0.097 0.623 0.429 0.800 1.001 7500

se[5,5] 0.689 0.083 0.694 0.520 0.839 1.001 7500

se[1,6] 0.181 0.074 0.170 0.067 0.353 1.001 7500

se[2,6] 0.846 0.072 0.857 0.680 0.953 1.001 7500

se[3,6] 0.857 0.075 0.871 0.680 0.966 1.001 7500

se[4,6] 0.620 0.124 0.625 0.371 0.847 1.001 7500

se[5,6] 0.633 0.110 0.637 0.406 0.836 1.001 7500

se[1,7] 0.215 0.074 0.206 0.097 0.385 1.001 3100

se[2,7] 0.862 0.067 0.873 0.704 0.959 1.001 7500

se[3,7] 0.873 0.070 0.885 0.706 0.973 1.001 7500

se[4,7] 0.612 0.118 0.616 0.373 0.829 1.001 7500

se[5,7] 0.631 0.106 0.636 0.414 0.820 1.001 4900

se[1,8] 0.189 0.078 0.179 0.064 0.364 1.001 7500

se[2,8] 0.857 0.069 0.869 0.701 0.960 1.001 7500

se[3,8] 0.868 0.072 0.881 0.698 0.971 1.001 3600

se[4,8] 0.568 0.145 0.569 0.293 0.844 1.001 7500

se[5,8] 0.800 0.110 0.818 0.548 0.959 1.001 7500

se[1,9] 0.187 0.054 0.180 0.099 0.310 1.001 5500

se[2,9] 0.855 0.052 0.861 0.733 0.937 1.001 7500

se[3,9] 0.869 0.059 0.877 0.732 0.957 1.001 5000

se[4,9] 0.613 0.082 0.615 0.447 0.772 1.001 7500

se[5,9] 0.670 0.067 0.673 0.530 0.792 1.001 6800

sp[1,1] 0.901 0.006 0.901 0.889 0.911 1.001 7500

sp[2,1] 0.780 0.007 0.780 0.765 0.794 1.001 7500

sp[3,1] 0.655 0.009 0.655 0.638 0.672 1.001 6500

sp[4,1] 1.000 0.000 1.000 0.999 1.000 1.001 7500

sp[5,1] 0.998 0.001 0.998 0.996 0.999 1.001 7500

sp[1,2] 0.889 0.004 0.889 0.882 0.897 1.001 7500

sp[2,2] 0.844 0.004 0.844 0.836 0.852 1.001 7500

sp[3,2] 0.660 0.006 0.660 0.648 0.672 1.001 2900

sp[4,2] 1.000 0.000 1.000 0.999 1.000 1.001 7500

sp[5,2] 0.998 0.001 0.998 0.996 0.999 1.001 7500

sp[1,3] 0.897 0.005 0.897 0.887 0.907 1.001 7500

sp[2,3] 0.826 0.006 0.826 0.814 0.838 1.001 7500

sp[3,3] 0.496 0.008 0.496 0.479 0.512 1.002 2700

sp[4,3] 1.000 0.000 1.000 0.999 1.000 1.001 7500

sp[5,3] 0.998 0.001 0.998 0.996 0.999 1.001 7500

sp[1,4] 0.891 0.004 0.891 0.883 0.898 1.001 7500

sp[2,4] 0.825 0.005 0.825 0.815 0.834 1.001 7500

sp[3,4] 0.738 0.006 0.738 0.727 0.749 1.001 6100

sp[4,4] 1.000 0.000 1.000 0.999 1.000 1.001 7500

sp[5,4] 0.998 0.001 0.998 0.996 0.999 1.001 7500

sp[1,5] 0.887 0.006 0.887 0.874 0.899 1.001 5600

sp[2,5] 0.922 0.005 0.922 0.911 0.932 1.001 7500

sp[3,5] 0.575 0.009 0.575 0.557 0.592 1.001 4800

sp[4,5] 1.000 0.000 1.000 0.999 1.000 1.001 7500

sp[5,5] 0.998 0.001 0.998 0.996 0.999 1.001 7500

sp[1,6] 0.898 0.006 0.898 0.886 0.909 1.001 7500

sp[2,6] 0.798 0.008 0.798 0.782 0.813 1.001 4800

sp[3,6] 0.665 0.009 0.665 0.647 0.682 1.001 4100

sp[4,6] 1.000 0.000 1.000 0.999 1.000 1.001 7500

sp[5,6] 0.998 0.001 0.998 0.996 0.999 1.001 7500

sp[1,7] 0.898 0.005 0.898 0.888 0.908 1.001 7500

sp[2,7] 0.764 0.007 0.764 0.750 0.777 1.001 6200

sp[3,7] 0.743 0.008 0.743 0.728 0.759 1.001 7500

sp[4,7] 1.000 0.000 1.000 0.999 1.000 1.001 7500

sp[5,7] 0.998 0.001 0.998 0.996 0.999 1.001 7500

sp[1,8] 0.886 0.008 0.886 0.870 0.901 1.001 5400

sp[2,8] 0.728 0.010 0.728 0.708 0.748 1.001 7500

sp[3,8] 0.706 0.014 0.707 0.677 0.731 1.001 7500

sp[4,8] 1.000 0.000 1.000 0.999 1.000 1.001 7500

sp[5,8] 0.998 0.001 0.998 0.996 0.999 1.001 7500

sp[1,9] 0.893 0.003 0.893 0.886 0.899 1.001 7500

sp[2,9] 0.825 0.004 0.825 0.817 0.832 1.001 7500

sp[3,9] 0.659 0.005 0.659 0.649 0.668 1.002 2800

sp[4,9] 1.000 0.000 1.000 0.999 1.000 1.001 7500

sp[5,9] 0.998 0.001 0.998 0.996 0.999 1.001 7500

taup[1] 0.160 0.088 0.143 0.039 0.374 1.001 7500

taup[2] 0.049 0.040 0.038 0.006 0.153 1.001 3800

theta[1,1] -0.756 0.027 -0.757 -0.811 -0.702 1.001 7500

theta[2,1] -0.392 0.032 -0.392 -0.455 -0.330 1.001 7500

theta[3,1] 1.013 0.035 1.013 0.944 1.084 1.001 6600

theta[4,1] 0.267 0.036 0.267 0.196 0.337 1.001 7500

theta[5,1] -0.709 0.066 -0.709 -0.838 -0.577 1.001 7500

theta[1,2] -0.168 0.021 -0.168 -0.210 -0.127 1.001 7500

theta[2,2] -0.371 0.033 -0.372 -0.436 -0.307 1.001 5200

theta[3,2] -0.485 0.033 -0.484 -0.550 -0.418 1.001 7500

theta[4,2] -0.561 0.046 -0.560 -0.653 -0.472 1.001 7500

deviance 71861.434 33.890 71859.551 71800.025 71931.829 1.001 7500

For each parameter, n.eff is a crude measure of effective sample size,

and Rhat is the potential scale reduction factor (at convergence, Rhat=1).

DIC info (using the rule, pD = var(deviance)/2)

pD = 574.4 and DIC = 72435.8

DIC is an estimate of expected predictive error (lower deviance is better).

1. Analysis with simultaneous imputation of missing Xpert Ultra and culture test results assuming the data is MNAR (Model adjusted for age, sex and HIV status)

Inference for Bugs model at "vb_model1_MNAR_3_groups_adj.txt", fit using jags,3 chains, each with 50000 iterations (first 25000 discarded), n.thin = 10 n.sims = 7500 iterations saved

mu.vect sd.vect 50% 2.5% 97.5% Rhat n.eff

a_prev[1] -2.709 0.128 -2.706 -2.962 -2.466 1.002 1900

a_prev[2] 0.289 0.123 0.286 0.055 0.532 1.001 7500

a_prev[3] 0.390 0.118 0.385 0.170 0.633 1.002 7500

a_prev[4] 0.152 0.143 0.152 -0.128 0.430 1.002 2400

a_prev[5] 0.299 0.143 0.293 0.038 0.582 1.005 7500

a_prev[6] 0.165 0.167 0.170 -0.168 0.486 1.002 2400

alpha[1,1,1] -0.872 0.050 -0.872 -0.971 -0.774 1.001 4600

alpha[2,1,1] -0.033 0.052 -0.033 -0.135 0.068 1.001 7500

alpha[3,1,1] -0.042 0.047 -0.042 -0.135 0.052 1.001 7500

alpha[4,1,1] -0.076 0.064 -0.076 -0.200 0.048 1.001 7500

alpha[5,1,1] -0.087 0.058 -0.087 -0.201 0.029 1.001 7500

alpha[6,1,1] -0.063 0.070 -0.063 -0.200 0.074 1.001 5200

alpha[1,2,1] -1.283 0.055 -1.284 -1.389 -1.172 1.002 1200

alpha[2,2,1] -0.108 0.053 -0.108 -0.210 -0.004 1.001 6300

alpha[3,2,1] 0.292 0.047 0.292 0.201 0.384 1.001 7500

alpha[4,2,1] 0.215 0.043 0.215 0.133 0.300 1.002 3100

alpha[5,2,1] 0.627 0.064 0.627 0.502 0.751 1.002 2400

alpha[6,2,1] 0.896 0.059 0.897 0.780 1.008 1.003 1100

alpha[7,2,1] 1.088 0.066 1.089 0.958 1.218 1.002 2600

alpha[1,3,1] -0.457 0.047 -0.457 -0.549 -0.364 1.001 6100

alpha[2,3,1] 0.060 0.051 0.060 -0.041 0.159 1.001 7200

alpha[3,3,1] 0.845 0.043 0.846 0.760 0.927 1.001 6800

alpha[4,3,1] 0.157 0.046 0.158 0.066 0.246 1.001 7500

alpha[5,3,1] 0.758 0.041 0.758 0.678 0.838 1.001 3600

alpha[6,3,1] -0.286 0.058 -0.286 -0.402 -0.172 1.001 7500

alpha[7,3,1] -0.441 0.054 -0.441 -0.545 -0.335 1.001 7500

alpha[8,3,1] -0.325 0.065 -0.325 -0.453 -0.199 1.001 7500

alpha[1,4,1] -3.424 0.185 -3.413 -3.815 -3.097 1.001 3400

alpha[1,5,1] -2.913 0.133 -2.904 -3.194 -2.677 1.001 7500

alpha[1,1,2] -0.985 0.089 -0.984 -1.161 -0.810 1.001 4100

alpha[2,1,2] -0.194 0.115 -0.193 -0.421 0.029 1.001 7500

alpha[3,1,2] -0.147 0.094 -0.146 -0.332 0.034 1.001 4200

alpha[4,1,2] 0.020 0.123 0.021 -0.225 0.256 1.001 7500

alpha[5,1,2] -0.057 0.115 -0.055 -0.288 0.161 1.002 2700

alpha[6,1,2] 0.141 0.128 0.143 -0.113 0.390 1.001 4400

alpha[1,2,2] -1.414 0.095 -1.414 -1.600 -1.225 1.001 3300

alpha[2,2,2] 0.181 0.114 0.184 -0.049 0.391 1.002 1900

alpha[3,2,2] 0.327 0.098 0.327 0.135 0.518 1.001 7500

alpha[4,2,2] 0.003 0.087 0.004 -0.169 0.174 1.002 2300

alpha[5,2,2] 0.528 0.116 0.528 0.297 0.755 1.001 7500

alpha[6,2,2] 0.632 0.109 0.631 0.420 0.849 1.002 1900

alpha[7,2,2] 0.943 0.125 0.943 0.701 1.188 1.001 7500

alpha[1,3,2] -0.123 0.076 -0.124 -0.273 0.023 1.001 7500

alpha[2,3,2] -0.183 0.103 -0.181 -0.386 0.019 1.001 7500

alpha[3,3,2] 0.762 0.092 0.763 0.578 0.946 1.002 1700

alpha[4,3,2] 0.126 0.092 0.126 -0.051 0.304 1.001 7500

alpha[5,3,2] 0.713 0.077 0.712 0.560 0.862 1.001 7500

alpha[6,3,2] -0.397 0.102 -0.397 -0.598 -0.198 1.001 4300

alpha[7,3,2] -0.813 0.097 -0.812 -1.001 -0.627 1.001 7500

alpha[8,3,2] -0.394 0.114 -0.395 -0.618 -0.169 1.001 7500

alpha[1,4,2] -3.424 0.185 -3.413 -3.815 -3.097 1.001 3400

alpha[1,5,2] -2.913 0.133 -2.904 -3.194 -2.677 1.001 7500

alpha[1,1,3] -3.516 0.408 -3.474 -4.424 -2.842 1.001 7500

alpha[2,1,3] -0.576 0.723 -0.522 -2.172 0.708 1.002 2400

alpha[3,1,3] -0.615 0.731 -0.551 -2.168 0.649 1.001 7500

alpha[4,1,3] -0.574 0.732 -0.533 -2.145 0.709 1.001 4100

alpha[5,1,3] -0.478 0.751 -0.415 -2.097 0.814 1.001 7500

alpha[6,1,3] -0.210 0.857 -0.184 -2.005 1.315 1.001 7500

alpha[1,2,3] -3.504 0.397 -3.467 -4.396 -2.838 1.002 1800

alpha[2,2,3] 0.005 0.999 -0.007 -1.930 1.946 1.001 7500

alpha[3,2,3] -0.590 0.727 -0.536 -2.139 0.688 1.001 7500

alpha[4,2,3] -0.597 0.728 -0.517 -2.190 0.652 1.001 7500

alpha[5,2,3] -0.576 0.722 -0.522 -2.123 0.685 1.001 7500

alpha[6,2,3] -0.499 0.769 -0.435 -2.167 0.822 1.001 4900

alpha[7,2,3] -0.213 0.862 -0.162 -1.987 1.346 1.001 6300

alpha[1,3,3] -0.963 0.043 -0.963 -1.046 -0.878 1.001 6100

alpha[2,3,3] 0.005 0.994 0.013 -1.983 1.946 1.001 3900

alpha[3,3,3] 0.000 1.005 0.000 -1.985 1.975 1.001 7500

alpha[4,3,3] -0.038 0.064 -0.038 -0.162 0.089 1.001 7500

alpha[5,3,3] 0.594 0.058 0.595 0.478 0.704 1.001 7500

alpha[6,3,3] -0.391 0.065 -0.391 -0.516 -0.265 1.001 7500

alpha[7,3,3] -0.913 0.111 -0.911 -1.139 -0.704 1.001 7500

alpha[8,3,3] -0.996 0.298 -0.984 -1.615 -0.448 1.001 5100

alpha[1,4,3] -3.424 0.185 -3.413 -3.815 -3.097 1.001 3400

alpha[1,5,3] -2.913 0.133 -2.904 -3.194 -2.677 1.001 7500

b_psi[1,1] -1.125 0.784 -1.150 -2.592 0.479 1.001 7500

b_psi[2,1] -0.002 1.006 -0.001 -2.006 1.961 1.001 7500

b_psi[1,2] -0.103 1.014 -0.120 -2.062 1.909 1.001 7500

b_psi[2,2] -0.005 0.991 -0.001 -1.981 1.957 1.001 7500

b_psi[1,3] -0.144 0.993 -0.152 -2.056 1.834 1.001 7500

b_psi[2,3] -0.013 0.987 -0.029 -1.891 1.967 1.001 7500

b_psi[1,4] -0.337 0.993 -0.385 -2.191 1.698 1.001 4900

b_psi[2,4] 0.001 0.988 0.007 -1.953 1.890 1.001 7500

b_tau[1] 0.002 0.330 0.002 -0.636 0.638 1.001 5600

b_tau[2] -1.415 0.266 -1.416 -1.936 -0.893 1.001 7500

beta[1,1,1] -1.177 0.304 -1.172 -1.781 -0.592 1.001 7500

beta[2,1,1] 0.240 0.306 0.232 -0.355 0.844 1.001 5100

beta[3,1,1] 0.043 0.313 0.046 -0.569 0.652 1.001 5900

beta[4,1,1] -0.080 0.339 -0.080 -0.743 0.579 1.001 7500

beta[5,1,1] 0.273 0.321 0.271 -0.349 0.909 1.001 7500

beta[6,1,1] 0.113 0.390 0.122 -0.677 0.853 1.001 7500

beta[1,2,1] 1.314 0.321 1.305 0.701 1.959 1.001 7500

beta[2,2,1] 0.296 0.374 0.292 -0.430 1.046 1.001 4200

beta[3,2,1] 0.019 0.340 0.020 -0.645 0.672 1.001 5000

beta[4,2,1] -0.037 0.368 -0.040 -0.748 0.700 1.001 7500

beta[5,2,1] 0.259 0.383 0.254 -0.477 1.047 1.001 7500

beta[6,2,1] 0.214 0.387 0.214 -0.538 0.976 1.001 7500

beta[1,3,1] 1.393 0.371 1.390 0.674 2.133 1.007 7500

beta[2,3,1] 0.376 0.373 0.385 -0.369 1.086 1.001 7500

beta[3,3,1] -0.005 0.371 -0.006 -0.727 0.732 1.001 7500

beta[4,3,1] 0.335 0.373 0.329 -0.388 1.059 1.001 7500

beta[5,3,1] -0.084 0.374 -0.090 -0.810 0.665 1.001 7500

beta[6,3,1] 0.220 0.394 0.212 -0.533 1.005 1.001 3800

beta[7,3,1] 0.103 0.415 0.101 -0.699 0.916 1.001 3900

beta[1,4,1] 0.299 0.307 0.295 -0.289 0.906 1.001 5200

beta[2,4,1] -0.290 0.318 -0.288 -0.909 0.329 1.001 5600

beta[3,4,1] 0.215 0.320 0.217 -0.410 0.831 1.001 7500

beta[4,4,1] 0.154 0.336 0.148 -0.501 0.821 1.001 7500

beta[5,4,1] 0.034 0.333 0.033 -0.609 0.678 1.001 7500

beta[6,4,1] -0.136 0.371 -0.146 -0.841 0.596 1.001 7500

beta[1,5,1] 0.671 0.353 0.670 -0.029 1.347 1.001 7500

beta[2,5,1] -0.015 0.366 -0.023 -0.709 0.694 1.001 7500

beta[3,5,1] -0.245 0.308 -0.242 -0.838 0.364 1.001 6900

beta[4,5,1] -0.180 0.318 -0.183 -0.800 0.467 1.001 7500

beta[5,5,1] -0.079 0.326 -0.083 -0.714 0.557 1.001 7500

beta[6,5,1] -0.188 0.332 -0.188 -0.835 0.455 1.001 7500

beta[7,5,1] 0.339 0.389 0.333 -0.405 1.135 1.001 7500

beta[1,1,2] -0.880 0.464 -0.875 -1.776 0.028 1.002 2300

beta[2,1,2] 0.039 0.451 0.044 -0.844 0.913 1.001 7500

beta[3,1,2] 0.058 0.450 0.057 -0.842 0.937 1.001 4400

beta[4,1,2] -0.007 0.444 -0.007 -0.882 0.856 1.001 7500

beta[5,1,2] 0.171 0.463 0.174 -0.750 1.066 1.002 1500

beta[6,1,2] -0.001 0.447 -0.001 -0.872 0.875 1.001 4700

beta[1,2,2] 1.052 0.438 1.055 0.197 1.903 1.001 3100

beta[2,2,2] 0.015 0.443 0.024 -0.854 0.867 1.001 7500

beta[3,2,2] 0.049 0.444 0.046 -0.824 0.924 1.001 6100

beta[4,2,2] 0.006 0.443 0.004 -0.866 0.874 1.001 4100

beta[5,2,2] 0.060 0.435 0.060 -0.798 0.915 1.001 3300

beta[6,2,2] -0.008 0.448 -0.008 -0.895 0.873 1.001 7500

beta[1,3,2] 0.978 0.448 0.978 0.087 1.855 1.001 5200

beta[2,3,2] -0.012 0.442 -0.009 -0.897 0.831 1.001 7500

beta[3,3,2] 0.027 0.441 0.027 -0.837 0.905 1.001 6200

beta[4,3,2] -0.042 0.451 -0.044 -0.918 0.829 1.001 7500

beta[5,3,2] -0.041 0.457 -0.051 -0.943 0.868 1.002 2800

beta[6,3,2] 0.074 0.439 0.077 -0.789 0.925 1.001 7500

beta[7,3,2] 0.002 0.443 0.006 -0.867 0.869 1.001 7500

beta[1,4,2] 0.299 0.307 0.295 -0.289 0.906 1.001 5200

beta[2,4,2] -0.290 0.318 -0.288 -0.909 0.329 1.001 5600

beta[3,4,2] 0.215 0.320 0.217 -0.410 0.831 1.001 7500

beta[4,4,2] 0.154 0.336 0.148 -0.501 0.821 1.001 7500

beta[5,4,2] 0.034 0.333 0.033 -0.609 0.678 1.001 7500

beta[6,4,2] -0.136 0.371 -0.146 -0.841 0.596 1.001 7500

beta[1,5,2] 0.671 0.353 0.670 -0.029 1.347 1.001 7500

beta[2,5,2] -0.015 0.366 -0.023 -0.709 0.694 1.001 7500

beta[3,5,2] -0.245 0.308 -0.242 -0.838 0.364 1.001 6900

beta[4,5,2] -0.180 0.318 -0.183 -0.800 0.467 1.001 7500

beta[5,5,2] -0.079 0.326 -0.083 -0.714 0.557 1.001 7500

beta[6,5,2] -0.188 0.332 -0.188 -0.835 0.455 1.001 7500

beta[7,5,2] 0.339 0.389 0.333 -0.405 1.135 1.001 7500

beta[1,1,3] -1.028 0.444 -1.030 -1.902 -0.162 1.001 7500

beta[2,1,3] -0.016 0.445 -0.024 -0.887 0.856 1.001 6000

beta[3,1,3] -0.018 0.433 -0.016 -0.866 0.841 1.001 7500

beta[4,1,3] -0.010 0.450 -0.007 -0.874 0.871 1.001 7500

beta[5,1,3] -0.001 0.450 0.000 -0.895 0.881 1.001 7500

beta[6,1,3] -0.002 0.443 -0.009 -0.878 0.878 1.001 5900

beta[1,2,3] 0.906 0.464 0.916 -0.023 1.802 1.001 7500

beta[2,2,3] -0.041 0.458 -0.037 -0.950 0.841 1.001 3000

beta[3,2,3] -0.037 0.460 -0.037 -0.939 0.888 1.001 7500

beta[4,2,3] -0.032 0.449 -0.029 -0.919 0.835 1.001 6800

beta[5,2,3] -0.015 0.446 -0.019 -0.872 0.867 1.001 7500

beta[6,2,3] -0.007 0.453 -0.008 -0.903 0.884 1.001 7000

beta[1,3,3] 0.996 0.446 1.004 0.110 1.867 1.002 2200

beta[2,3,3] -0.002 0.448 0.001 -0.893 0.863 1.001 7500

beta[3,3,3] -0.001 0.447 0.000 -0.882 0.872 1.001 7500

beta[4,3,3] -0.005 0.448 -0.002 -0.891 0.878 1.001 7500

beta[5,3,3] 0.005 0.457 -0.003 -0.889 0.905 1.001 7500

beta[6,3,3] 0.004 0.452 0.006 -0.873 0.880 1.001 7500

beta[7,3,3] 0.003 0.446 0.005 -0.864 0.864 1.002 2500

beta[1,4,3] 0.299 0.307 0.295 -0.289 0.906 1.001 5200

beta[2,4,3] -0.290 0.318 -0.288 -0.909 0.329 1.001 5600

beta[3,4,3] 0.215 0.320 0.217 -0.410 0.831 1.001 7500

beta[4,4,3] 0.154 0.336 0.148 -0.501 0.821 1.001 7500

beta[5,4,3] 0.034 0.333 0.033 -0.609 0.678 1.001 7500

beta[6,4,3] -0.136 0.371 -0.146 -0.841 0.596 1.001 7500

beta[1,5,3] 0.671 0.353 0.670 -0.029 1.347 1.001 7500

beta[2,5,3] -0.015 0.366 -0.023 -0.709 0.694 1.001 7500

beta[3,5,3] -0.245 0.308 -0.242 -0.838 0.364 1.001 6900

beta[4,5,3] -0.180 0.318 -0.183 -0.800 0.467 1.001 7500

beta[5,5,3] -0.079 0.326 -0.083 -0.714 0.557 1.001 7500

beta[6,5,3] -0.188 0.332 -0.188 -0.835 0.455 1.001 7500

beta[7,5,3] 0.339 0.389 0.333 -0.405 1.135 1.001 7500

pi[1] 0.011 0.004 0.011 0.006 0.019 1.003 2700

pi[2] 0.005 0.002 0.005 0.003 0.008 1.011 890

pi[3] 0.013 0.004 0.012 0.008 0.021 1.013 1700

pi[4] 0.005 0.001 0.005 0.003 0.007 1.002 1800

pi[5] 0.005 0.001 0.005 0.002 0.008 1.003 1000

pi[6] 0.007 0.002 0.007 0.004 0.012 1.002 6900

pi[7] 0.010 0.004 0.010 0.006 0.018 1.012 1300

pi[8] 0.008 0.003 0.007 0.003 0.014 1.001 6400

pi[9] 0.018 0.005 0.017 0.012 0.027 1.013 1100

pi[10] 0.003 0.001 0.002 0.001 0.005 1.005 1900

pi[11] 0.001 0.001 0.001 0.000 0.002 1.001 4400

pi[12] 0.008 0.002 0.007 0.005 0.012 1.017 1100

psi[1,1] 0.185 0.182 0.125 0.005 0.684 1.001 7500

psi[2,1] 0.499 0.289 0.500 0.022 0.975 1.001 7500

psi[1,2] 0.470 0.290 0.452 0.020 0.972 1.001 7500

psi[2,2] 0.498 0.288 0.499 0.024 0.975 1.001 7500

psi[1,3] 0.458 0.287 0.440 0.020 0.967 1.001 6500

psi[2,3] 0.495 0.287 0.488 0.029 0.975 1.001 7500

psi[1,4] 0.401 0.282 0.350 0.014 0.955 1.001 3900

psi[2,4] 0.501 0.288 0.503 0.025 0.971 1.001 7500

pv[1] 0.502 0.005 0.502 0.492 0.511 1.001 7500

pv[2] 0.144 0.004 0.144 0.137 0.151 1.001 7500

pv[3] 0.354 0.005 0.354 0.345 0.364 1.001 5600

se[1,1] 0.212 0.072 0.203 0.094 0.376 1.001 7500

se[2,1] 0.847 0.075 0.858 0.677 0.959 1.001 7500

se[3,1] 0.861 0.074 0.874 0.686 0.967 1.001 5000

se[4,1] 0.551 0.119 0.553 0.313 0.774 1.002 7000

se[5,1] 0.602 0.108 0.606 0.377 0.795 1.003 2100

se[1,2] 0.180 0.054 0.175 0.091 0.300 1.001 7500

se[2,2] 0.845 0.056 0.852 0.721 0.934 1.001 7500

se[3,2] 0.871 0.060 0.881 0.729 0.958 1.001 4700

se[4,2] 0.643 0.092 0.647 0.454 0.807 1.009 2000

se[5,2] 0.698 0.076 0.704 0.537 0.829 1.006 7500

se[1,3] 0.192 0.067 0.185 0.084 0.344 1.001 7500

se[2,3] 0.839 0.071 0.850 0.677 0.947 1.001 7500

se[3,3] 0.871 0.071 0.884 0.704 0.971 1.001 7500

se[4,3] 0.674 0.109 0.681 0.438 0.859 1.019 2400

se[5,3] 0.637 0.097 0.644 0.429 0.805 1.013 2400

se[1,4] 0.189 0.056 0.182 0.096 0.311 1.001 7500

se[2,4] 0.849 0.057 0.856 0.720 0.938 1.001 7500

se[3,4] 0.867 0.060 0.877 0.726 0.956 1.001 3800

se[4,4] 0.587 0.096 0.589 0.393 0.770 1.003 3900

se[5,4] 0.685 0.082 0.691 0.514 0.831 1.002 7500

se[1,5] 0.171 0.053 0.166 0.083 0.288 1.001 7500

se[2,5] 0.847 0.056 0.854 0.719 0.935 1.001 7500

se[3,5] 0.873 0.060 0.883 0.730 0.960 1.001 4400

se[4,5] 0.624 0.099 0.628 0.416 0.807 1.005 2600

se[5,5] 0.690 0.086 0.696 0.504 0.838 1.001 7500

se[1,6] 0.183 0.072 0.174 0.068 0.347 1.001 7500

se[2,6] 0.834 0.078 0.846 0.660 0.950 1.001 7500

se[3,6] 0.856 0.076 0.870 0.677 0.964 1.001 7500

se[4,6] 0.626 0.125 0.630 0.376 0.856 1.002 7500

se[5,6] 0.636 0.112 0.642 0.405 0.834 1.002 3200

se[1,7] 0.222 0.076 0.214 0.098 0.394 1.001 7500

se[2,7] 0.855 0.070 0.867 0.692 0.960 1.001 7500

se[3,7] 0.875 0.071 0.888 0.703 0.974 1.001 5600

se[4,7] 0.611 0.123 0.618 0.348 0.831 1.007 2200

se[5,7] 0.625 0.113 0.634 0.379 0.817 1.009 3800

se[1,8] 0.190 0.078 0.182 0.065 0.367 1.001 7500

se[2,8] 0.850 0.072 0.860 0.683 0.958 1.001 7500

se[3,8] 0.867 0.073 0.881 0.693 0.971 1.002 1800

se[4,8] 0.570 0.146 0.571 0.278 0.843 1.001 7500

se[5,8] 0.798 0.112 0.815 0.537 0.961 1.002 7500

se[1,9] 0.190 0.054 0.184 0.100 0.309 1.001 7500

se[2,9] 0.846 0.056 0.853 0.719 0.934 1.001 7500

se[3,9] 0.868 0.060 0.878 0.727 0.956 1.001 4900

se[4,9] 0.615 0.086 0.619 0.438 0.775 1.010 2500

se[5,9] 0.670 0.072 0.674 0.518 0.796 1.009 3300

sp[1,1] 0.901 0.006 0.901 0.890 0.912 1.001 4600

sp[2,1] 0.781 0.008 0.781 0.766 0.796 1.001 5900

sp[3,1] 0.655 0.009 0.655 0.638 0.673 1.002 1500

sp[4,1] 1.000 0.000 1.000 0.999 1.000 1.001 3100

sp[5,1] 0.998 0.001 0.998 0.996 0.999 1.001 7500

sp[1,2] 0.889 0.004 0.889 0.882 0.897 1.001 5800

sp[2,2] 0.845 0.004 0.845 0.836 0.853 1.001 7500

sp[3,2] 0.660 0.006 0.660 0.648 0.672 1.001 7500

sp[4,2] 1.000 0.000 1.000 0.999 1.000 1.001 3200

sp[5,2] 0.998 0.001 0.998 0.996 0.999 1.001 7500

sp[1,3] 0.898 0.005 0.898 0.887 0.908 1.001 3000

sp[2,3] 0.827 0.006 0.826 0.814 0.839 1.004 4500

sp[3,3] 0.496 0.009 0.496 0.479 0.513 1.001 5900

sp[4,3] 1.000 0.000 1.000 0.999 1.000 1.001 3100

sp[5,3] 0.998 0.001 0.998 0.996 0.999 1.001 7500

sp[1,4] 0.890 0.004 0.891 0.882 0.898 1.001 5500

sp[2,4] 0.825 0.005 0.825 0.816 0.834 1.001 7500

sp[3,4] 0.738 0.006 0.738 0.727 0.749 1.002 1800

sp[4,4] 1.000 0.000 1.000 0.999 1.000 1.001 3200

sp[5,4] 0.998 0.001 0.998 0.996 0.999 1.001 7500

sp[1,5] 0.886 0.006 0.886 0.874 0.899 1.001 3200

sp[2,5] 0.922 0.005 0.923 0.912 0.932 1.002 2000

sp[3,5] 0.575 0.009 0.575 0.557 0.592 1.001 7500

sp[4,5] 1.000 0.000 1.000 0.999 1.000 1.001 3100

sp[5,5] 0.998 0.001 0.998 0.996 0.999 1.001 7500

sp[1,6] 0.898 0.006 0.898 0.886 0.910 1.001 7500

sp[2,6] 0.798 0.008 0.798 0.783 0.813 1.001 7500

sp[3,6] 0.665 0.009 0.665 0.647 0.682 1.001 7500

sp[4,6] 1.000 0.000 1.000 0.999 1.000 1.001 3100

sp[5,6] 0.998 0.001 0.998 0.996 0.999 1.001 7500

sp[1,7] 0.899 0.005 0.899 0.888 0.909 1.001 5700

sp[2,7] 0.764 0.007 0.764 0.750 0.779 1.003 1700

sp[3,7] 0.744 0.008 0.744 0.728 0.760 1.002 2600

sp[4,7] 1.000 0.000 1.000 0.999 1.000 1.001 3100

sp[5,7] 0.998 0.001 0.998 0.996 0.999 1.001 7500

sp[1,8] 0.886 0.008 0.886 0.870 0.901 1.001 7500

sp[2,8] 0.728 0.010 0.728 0.707 0.748 1.001 7500

sp[3,8] 0.706 0.014 0.707 0.677 0.731 1.002 2600

sp[4,8] 1.000 0.000 1.000 0.999 1.000 1.001 3100

sp[5,8] 0.998 0.001 0.998 0.996 0.999 1.001 7500

sp[1,9] 0.893 0.003 0.893 0.886 0.899 1.001 3400

sp[2,9] 0.825 0.004 0.825 0.818 0.833 1.001 7500

sp[3,9] 0.659 0.005 0.659 0.649 0.669 1.002 2100

sp[4,9] 1.000 0.000 1.000 0.999 1.000 1.001 3100

sp[5,9] 0.998 0.001 0.998 0.996 0.999 1.001 7500

tau[1] 0.501 0.125 0.501 0.263 0.738 1.001 5300

tau[2] 0.086 0.042 0.078 0.026 0.186 1.001 7500

taup[1] 0.331 0.075 0.333 0.185 0.468 1.001 5500

taup[2] 0.140 0.065 0.129 0.044 0.293 1.001 7500

theta[1,1] -0.757 0.027 -0.757 -0.809 -0.705 1.001 7500

theta[2,1] -0.393 0.031 -0.393 -0.455 -0.331 1.001 7500

theta[3,1] 1.013 0.035 1.013 0.944 1.082 1.001 7500

theta[4,1] 0.267 0.036 0.267 0.195 0.338 1.001 4800

theta[5,1] -0.707 0.066 -0.707 -0.837 -0.581 1.001 7500

theta[1,2] -0.169 0.022 -0.169 -0.211 -0.127 1.001 6900

theta[2,2] -0.371 0.033 -0.371 -0.436 -0.307 1.001 5500

theta[3,2] -0.485 0.034 -0.485 -0.551 -0.418 1.001 7500

theta[4,2] -0.561 0.048 -0.560 -0.657 -0.468 1.001 7500

deviance 71839.101 50.829 71840.714 71756.543 71924.178 1.051 590

For each parameter, n.eff is a crude measure of effective sample size,

and Rhat is the potential scale reduction factor (at convergence, Rhat=1).

DIC info (using the rule, pD = var(deviance)/2)

pD = 1287.7 and DIC = 73126.8

DIC is an estimate of expected predictive error (lower deviance is better).

**References**

[1] Menten J, Boelaert M, Lesaffre E. Bayesian latent class models with conditionally dependent diagnostic tests: A case study. Stat Med 2008;27:4469–88. https://doi.org/10.1002/sim.3317.

[2] Keter AK, Lynen L, Van Heerden A, Wong E, Reither K, Goetghebeur E, et al. Evaluation of tuberculosis diagnostic test accuracy using Bayesian latent class analysis in the presence of conditional dependence between the diagnostic tests used in a community-based tuberculosis screening study. PLoS One 2023;18:e0282417. https://doi.org/10.1371/journal.pone.0282417.

[3] Albert JH, Chib S. Bayesian Analysis of Binary and Polychotomous Response Data. American Statistical Association 1993;88.

[4] Gelman A, Carlin JB, Stern HS, Dunson DB, Vehtari A, Rubin DB. Bayesian Data Analysis. Third Edition. New York: Chapman and Hall; 2014.

[5] Little RJA, Rubin DB. Statistical Inference with Missing Data. Second Edition. New York: Wiley; 2002.

[6] van Buuren S, Groothuis-Oudshoorn K. mice: Multivariate imputation by chained equations in R. J Stat Softw 2011;45:1–67. https://doi.org/10.18637/jss.v045.i03.

[7] Moyo S, Ismail F, Van der Walt M, Ismail N, Mkhondo N, Dlamini S, et al. Prevalence of bacteriologically confirmed pulmonary tuberculosis in South Africa, 2017–19: a multistage, cluster-based, cross-sectional survey. Lancet Infect Dis 2022;22:1172–80. https://doi.org/10.1016/S1473-3099(22)00149-9.
