# Supplementary material for: Simultaneous alleviation of verification and reference standard biases in a community-based tuberculosis screening study using Bayesian latent class analysis
Source: PLoS One. 2024 Jun 10;19(6):e0305126. doi: 10.1371/journal.pone.0305126 (PMC11164341; doi:10.1371/journal.pone.0305126)
Supplement: Attachment — Submitted filename: Response to reviewers.pdf [file pone.0305126.s002.pdf]

INSTITUTE OF TROPICAL MEDICINE ANTWERP

Department of Clinical Sciences
TB/HIV Unit
Nationalestraat 155
2000 Antwerpen
Belgium

The Editor,
PLOS ONE

**YOUR REF.**	**OUR REF.**	**ATTACHMENT(S)**	**ANTWERP**
			22/03/2024

Subject: Manuscript Re-submission

Dear Dr. Chegou,

On behalf of the co-authors, I would like to sincerely thank you for allowing our manuscript entitled "**Simultaneous alleviation of verification and reference standard biases in a community-based tuberculosis screening study using Bayesian latent class analysis**" undergo peer-review. We would also like to thank you and the reviewer for taking time to review our manuscript and for the insightful feedback that has helped us improve the quality of the manuscript.

We wish to resubmit our revised manuscript after considering all the suggestions from the editors and the reviewer. We have particularly revised the manuscript to conform to PLOS ONE's journal style requirements, have addressed the question on the ethical approval of the study, and responded to the questions and suggestions of the reviewer. These responses are presented with this letter in the next page.

We hope that our responses will be able to sufficiently address the questions raised. Nevertheless, we are happy to respond in case further clarification is required.

We look forward to hearing from you.

Thank you on behalf of the co-authors.

Alfred K. Keter

Corresponding author

Comments to the Author

1. Is the manuscript technically sound, and do the data support the conclusions?

The manuscript must describe a technically sound piece of scientific research with data that supports the conclusions. Experiments must have been conducted rigorously, with appropriate controls, replication, and sample sizes. The conclusions must be drawn appropriately based on the data presented.

Reviewer #1: Yes

---

2. Has the statistical analysis been performed appropriately and rigorously?

Reviewer #1: Yes

---

3. Have the authors made all data underlying the findings in their manuscript fully available?

The PLOS Data policy requires authors to make all data underlying the findings described in their manuscript fully available without restriction, with rare exception (please refer to the Data Availability Statement in the manuscript PDF file). The data should be provided as part of the manuscript or its supporting information, or deposited to a public repository. For example, in addition to summary statistics, the data points behind means, medians and variance measures should be available. If there are restrictions on publicly sharing data—e.g. participant privacy or use of data from a third party—those must be specified.

Reviewer #1: Yes

---

4. Is the manuscript presented in an intelligible fashion and written in standard English?

PLOS ONE does not copyedit accepted manuscripts, so the language in submitted articles must be clear, correct, and unambiguous. Any typographical or grammatical errors should be corrected at revision, so please note any specific errors here.

Reviewer #1: Yes

---

5. Review Comments to the Author

Please use the space provided to explain your answers to the questions above. You may also include additional comments for the author, including concerns about dual publication, research ethics, or publication ethics. (Please upload your review as an attachment if it exceeds 20,000 characters)

Reviewer #1: Many thanks for asking me to review this manuscript. Really important methodological study, addressing an issue that we (and many other researchers) have faced when analysing TB prevalence surveys and the accuracy of novel TB diagnostics. Clearly written, and high standard of analysis. I have only a few issues the authors should address:

1. Line 81 and 82: Suggest "higher risk" rather than "high risk".

Thank you for this suggestion. We have revised the sentence as suggested.

2. Fraction of participants in the Vukuzazi study eligible to submit sputum (65%) and prevalence of TB (3.3% in those sputum tested) were higher than in most other national and sub-national prevalence surveys. In the discussion, I would recommend that the authors reflect on whether their results would likely hold when evaluated in settings with less intense TB epidemics.

The data presented in the question are from two different sources: fraction eligible for verification - Vukuzazi study, and the prevalence of TB – from the simulation. Nonetheless, we will try to handle this question from two angles. First, the simulation used a TB prevalence of 3.3% among the participants eligible and verified to demonstrate a high-risk group manifesting with symptoms and/or abnormal chest X-ray images. Our goal was to demonstrate the impact of excluding the ineligible and unverified participants from the estimation of prevalence. Analysis of the simulated data yielded the true values used to generate the data. We are confident that if repeated on simulated data using different parameters e.g. lower true values of the prevalence the model would yield correct estimates. In a low prevalence setting, less people would pass the screening step. If a screening test meeting the WHO minimum target product profiles (i.e. sensitivity of 90% and specificity of 70%) is applied on a population setting with 1% prevalence, the resulting estimate of prevalence in the microbiologically tested population is about 3.3%. However, if such tests have a higher specificity in the general population screening (which isn't that unlikely), then a 2-3% prevalence in the microbiologically tested population is still possible with 0.5% prevalence and 80% specificity of the triage test for example. From our analysis of the eligible and verified participants in the Vukuzazi study, we obtained TB prevalence of 1.3% using Bayesian LCA and 1.4% using composite reference standard. Upon accounting for the unverified

participants, the estimate based on Bayesian LCA was even lower and closer to the reported overall population prevalence. When adapted appropriately with the use of realistic priors, the model can yield realistic estimates of TB prevalence in regions with less intense TB epidemics.
As proposed by the reviewer, we have added the following paragraph to the discussion.

"While our proposed model yields realistic estimates, it cannot be applied directly in a different setting without prior consideration of the diagnostic tests in use, the potential dependencies between the diagnostic tests and the underlying prevalence. The concept is the same but the model will need to be tweaked appropriately. Based on our analysis of active TB case-finding data, regions with low prevalence may require specification of a weakly-informative prior to ensure convergence of the prevalence to a realistic distribution. The factors associated with TB prevalence as well as the diagnostic test properties may be included in the analysis to improve the precision of the estimates. "

3. Building upon point 2 above, external validation against other prevalence survey datasets would provide greater confidence in the results of simulations. I don't think the authors need to do this for the present manuscript, but I wonder if for a future paper, comparison of methods using the SCALE dataset from Malawi (https://journals.plos.org/globalpublichealth/article?id=10.1371/journal.pgph.0001911), the TREATS study from Zambia (https://journals.plos.org/plosmedicine/article?id=10.1371/journal.pmed.1004278), or from recent national TB prevalence surveys might be informative. Either way, I think a sentence in the Discussion should mention external validation.

We wish to thank the reviewer for this suggestion.

Indeed, our methods are currently being applied in the analysis of a number of population-based TB prevalence surveys. Specifically, the methods are being applied in the analysis of ZAMSTAR survey, TREATS study and Bangladesh survey among others. There is an ongoing collaboration between the Institute of Tropical Medicine in Antwerp, Heidelberg University in Germany, Swiss Tropical and Public Health Institute in Switzerland and other partners who are conducting analyses to externally validate these methods in six TB prevalence surveys. We would be glad to collaborate with the reviewer of our manuscript to validate and compare the methods in future work. We have added the paragraph below to the discussion highlighting the ongoing external validations using other datasets.

"We have tested our methods using simulation analysis and validated them using only one community-based TB prevalence survey. It would be interesting to understand the performance of the model in several other prevalence surveys. Currently, there is an ongoing effort to externally validate these methods in six TB prevalence surveys."

4. In the methods, would be good to mention priors that were selected for each parameter, and how the influence of prior selection was checked. (I see there is a table of priors in the supplemental material - prior choice can be highly influential in these sorts of models).

Thank you for this comment. We have added the paragraph below to the methods section.

The unknown model parameters were assigned priors from the Gaussian distribution. Except for the unknown parameters of the prevalence model and the regressive probit models for the specificity of Xpert Ultra and culture, the unknown model parameters in all the models were assigned priors from Gaussian distribution with mean zero and unit variance. The unknown model parameters for the overall prevalence and overall false positive rate (equal to 1-specificity) for Xpert Ultra and culture were assigned priors from Gaussian distribution with mean -3 and variance of 0.1. The structure of the models and the priors assigned to the unknown model parameters are presented in S9 and S10 Tables of the Supplementary Material. The choice of the informative prior for the overall prevalence was based on data from the literature on the estimates of population prevalence of PTB in the region where the study was conducted. Similarly, the choice of the informative priors for the overall specificity for Xpert Ultra and culture were based on the literature on the performance of these tests when compared to the composite reference standard and on the expert knowledge. In the absence of TB, culture for M*ycobacterium tuberculosis* has a high specificity. Xpert Ultra also has a substantially high specificity when evaluated against culture. This explains the choice of our priors for the unknown model parameters for the specificity of Xpert Ultra and culture.

5. I note that the authors state in several places that this is the first time this issue has been addressed. Although the authors have done a much more advanced analysis here, we previously undertook a similar approach (albeit without the simulation study) using data from the Kenya National TB prevalence survey (https://journals.plos.org/globalpublichealth/article?id=10.1371/journal.pgph.0001272). Good to reflect on differences in the approach by Mungai et al and the present paper in the discussion.

Thank you for this comment.

Indeed, to the best of our knowledge this is the first application of Bayesian LCA to simultaneously alleviate the reference standard bias as well as the verification bias. Our approach acknowledges that there is no perfect reference standard for conclusive determination of TB. Even culture for *Mycobacterium tuberculosis* has an imperfect sensitivity. Hence, a negative culture test result does not rule out the presence of TB.

Using all the available information on the diagnostic tests involved in the diagnosis of TB, without considering any as the reference test, Bayesian LCA was able to

stochastically classify the participants into those with and without TB. We extended the model to allow imputation of the unknown TB status among those who were not bacteriologically tested because they were ineligible based on the screening criteria. In doing so, the model was able to yield estimates of TB prevalence and diagnostic test accuracy (sensitivity and specificity) corrected for the verification bias.

This approach differs from the application in Mungai et al. 2022 who used Bayesian LCA to infer the prevalence of TB among the participants who were not bacteriologically tested while using Xpert and culture as the bacteriological reference standard among the bacteriologically tested participants.

We have added the above paragraph into our discussion to highlight the difference between these two approaches.

6. In the supplemental material, trace plots in Figure 3.1 show several chains for parameters with potential anomalies suggestive of serial correlation, likely caused by difficulties exploring the sample sample and low effective samples. It is well known that achieving convergence in LCA models can be challenging. In the supplemental material, the authors should a) describe methods to improve sampling (e.g. potentially related to point 4 above), and b) present a table showing Rhat statistics and effective sample sizes for each parameter.

Thank you for this comment.

We acknowledge that convergence can be problematic with Bayesian LCA. This limitation can be overcome by carefully choosing priors for the unknown model parameters. Informative priors for certain parameters can be chosen based on the expert knowledge and the available data in the literature. This can improve model convergence. Increasing the number of iterations and choosing the correct "burn-in" or "warm-up" period in the MCMC sampling algorithm can also help achieve good mixing. Using a wider sampling interval can reduce autocorrelation.

Nevertheless, the trace plots presented in S3 Fig. in the Supplementary Material (Figure 3.1 in the previous version of the Supplementary Material) depict convergence in the imputation models used to impute the missing Xpert Ultra and culture test results as well as the HIV status among the microbiologically untested participants. This imputation was implemented using Multivariate Imputation via Chained Equations (MICE) in R statistical software. Except for a few instances where sampling in one of the chains appears a little distant from the rest of the chains, the sampling seems to converge to some value for each of the three presented variables. The reason for the conspicuous anomalies may be explained by the choice of the axis scale which is determined inside the *mice* package. In the package, 5-10 iterations are recommended but, in our case, we used 50 iterations which provides a better exploration of the sampling space.

6. PLOS authors have the option to publish the peer review history of their article (what does this mean?). If published, this will include your full peer review and any attached files.

If you choose "no", your identity will remain anonymous but your review may still be made public.

We consent to publication of the peer review history of our article.

Do you want your identity to be public for this peer review? For information about this choice, including consent withdrawal, please see our Privacy Policy.

Reviewer #1: **Yes:** Peter MacPherson

Journal Requirements:

When submitting your revision, we need you to address these additional requirements.

1. Please ensure that your manuscript meets PLOS ONE's style requirements, including those for file naming. The PLOS ONE style templates can be found at

https://journals.plos.org/plosone/s/file?id=wjVg/PLOSOne_formatting_sample_main_body.pdf and https://journals.plos.org/plosone/s/file?id=ba62/PLOSOne_formatting_sample_title_authors_affiliations.pdf

Thank you for this comment. We have formatted the manuscript and Supplementary Material to conform to the PLOS ONE’s style requirements.

2. Please be informed that funding information should not appear in the Acknowledgments section or other areas of your manuscript. We will only publish funding information present in the Funding Statement section of the online submission form. Please remove any funding-related text from the manuscript.

Thanks for this comment. We have removed the funding information from the body of the manuscript. We will ensure that this information is only available in the Funding Statement section of the online submission form.

3. For studies involving third-party data, we encourage authors to share any data specific to their analyses that they can legally distribute. PLOS recognizes, however, that authors may be using third-party data they do not have the rights to share. When third-party data cannot be publicly shared, authors must provide all information necessary for interested researchers to apply to gain access to the data. (https://journals.plos.org/plosone/s/data-availability#loc-acceptable-data-access-restrictions)

For any third-party data that the authors cannot legally distribute, they should include the following information in their Data Availability Statement upon submission:
1) A description of the data set and the third-party source
2) If applicable, verification of permission to use the data set
3) Confirmation of whether the authors received any special privileges in accessing the data that other researchers would not have
4) All necessary contact information others would need to apply to gain access to the data

Thank you for this comment. We have provided information on the availability of the data at the end of the manuscript. We also provided similar information in the Data availability statement during submission as follows

This work was based on simulations and analysis of a real dataset. The simulation and the analysis scripts are available on github (https://github.com/KeterAlfred). We obtained the real dataset from Africa Health Research Institute (AHRI) upon putting in a formal request where we entered a binding agreement to use the data solely for the intended research purpose. Data is publicly available but AHRI owns and controls access and use of the data. Upon request, the dataset can be accessed at the AHRI data repository available in this link:

https://data.ahri.org/index.php/catalog/990/study-description#metadata-data_access. The analysis scripts for this dataset are also available on github (https://github.com/KeterAlfred).

4. Please include captions for your Supporting Information files at the end of your manuscript, and update any in-text citations to match accordingly. Please see our Supporting Information guidelines for more information: http://journals.plos.org/plosone/s/supporting-information.

Thank you for this comment. Captions of the Supporting Information has been included at the end of the manuscript.

5. Please review your reference list to ensure that it is complete and correct. If you have cited papers that have been retracted, please include the rationale for doing so in the manuscript text, or remove these references and replace them with relevant current references. Any changes to the reference list should be mentioned in the rebuttal letter that accompanies your revised manuscript. If you need to cite a retracted article, indicate the article's retracted status in the References list and also include a citation and full reference for the retraction notice.

Thank you for this feedback.

We have reviewed the entire reference list. We did not find any article that has been retracted in our list of references. Included in our citations and the reference list are articles that had corrections made to them. These included the references listed in number 4, 5 and 20. We included the erratum for reference number 20 but not for reference numbers 4 and 5. We have also added the errata files for reference numbers 4 and 5. We regret this omission.

Additional Editor Comments:

You wrote "NA" under "Ethics statement" in the manuscript submission system. Although this was a secondary analysis of data that was collected via a community-based study, the original study protocol must have been approved by an ethics committee. In the case where only data analysis is done as may be the case here, it is customary in most environments that an ethics committee should still be consulted, for an exemption from ethics certificate if deemed necessary.

Please revise the ethics section in the submission system and add an ethics statement to the main manuscript. Say whether the study was approved (or exemption granted) by an Ethics committee or not.

Thank you for this comment.

The form previously shared with me was prefilled with 'NA' for every question. I didn't delete them as I did not understand the implications. Nevertheless, I added my responses wherever it was necessary. I have revised the form by deleting the prefilled "NA".
The protocol for the primary study was approved by the Research Ethics Committee prior to its implementation. However, for this manuscript, we obtained the dataset from Africa Health Research Institute (AHRI) upon putting in a formal request where we entered into a binding agreement to use the data solely for the intended research purpose. Data is publicly available but AHRI owns and controls access and use of the data. Upon request, the dataset can be accessed at the AHRI data repository available in this link: https://data.ahri.org/index.php/catalog/990/study-description#metadata-data_access.
Our study was not separately evaluated by an Ethics committee because it involved the evaluation of statistical methods using simulations and publicly available data, i.e. a methodological rather than a medical research question. The data was therefore used as a proof of concept here, not a research goal by itself. In hindsight, we would have been able to obtain an exemption from our Institutional Review Board had we requested it, but this is no longer possible post-hoc.

We have added the ethics statement to the main manuscript as follows
“Our study was not separately evaluated by an Ethics committee because it involved the evaluation of statistical methods using simulations and publicly available data, i.e. a methodological rather than a medical research question.”
