# Supplementary material for: Simultaneous alleviation of verification and reference standard biases in a community-based tuberculosis screening study using Bayesian latent class analysis
Source: PLoS One. 2024 Jun 10;19(6):e0305126. doi: 10.1371/journal.pone.0305126 (PMC11164341; doi:10.1371/journal.pone.0305126)
Supplement: Attachment — Submitted filename: Response to reviewers.pdf [file pone.0305126.s003.pdf]

Department of Clinical Sciences
TB/HIV Unit
Nationalestraat 155
2000 Antwerpen
Belgium

The Editor,
PLOS ONE

**YOUR REF.**	**OUR REF.**	**ATTACHMENT(S)**	**ANTWERP**
			20/05/2024

Subject: Manuscript resubmission

Dear Dr. Chegou,

On behalf of the co-authors, I would like to sincerely thank you for allowing our manuscript entitled "**Simultaneous alleviation of verification and reference standard biases in a community-based tuberculosis screening study using Bayesian latent class analysis**" undergo peer-review. We would also like to thank you and the reviewer for taking time to review our manuscript and for the insightful feedback that has helped us improve the quality of the manuscript.

We wish to resubmit our revised manuscript after considering all the suggestions from the editors and the reviewer. We have particularly revised the manuscript to conform to PLOS ONE's journal style requirements, citation and referencing of retracted articles and responded to the suggestion of the reviewer to include model results showing model convergence. These responses are presented with this letter in the next page.

We hope that our responses will be able to sufficiently address the questions raised. Nevertheless, we are happy to respond in case further clarification is required.

We look forward to hearing from you.

Thank you on behalf of the co-authors.

Alfred K. Keter

Corresponding author

**Date:**	Apr 23 2024 04:01PM
**To:**	"Alfred Kipyegon Keter" keteralfred@gmail.com
**cc:**	novel@sun.ac.za
**From:**	"PLOS ONE" plosone@plos.org
**Subject:**	PLOS ONE Decision: Revision required [PONE-D-24-00503R1]

PONE-D-24-00503R1
Simultaneous alleviation of verification and reference standard biases in a community-based tuberculosis screening study using Bayesian latent class analysis
PLOS ONE

Dear Dr. Keter,

Thank you for submitting your manuscript to PLOS ONE. After careful consideration, we feel that it has merit but does not fully meet PLOS ONE's publication criteria as it currently stands. Therefore, we invite you to submit a revised version of the manuscript that addresses the points raised during the review process.

==============================
ACADEMIC EDITOR:
Please address the outstanding important query raised by the reviewer.
==============================

Please submit your revised manuscript by Jun 07 2024 11:59PM. If you will need more time than this to complete your revisions, please reply to this message or contact the journal office at plosone@plos.org. When you're ready to submit your revision, log on to https://www.editorialmanager.com/pone/ and select the 'Submissions Needing Revision' folder to locate your manuscript file.

Please include the following items when submitting your revised manuscript:

- A rebuttal letter that responds to each point raised by the academic editor and reviewer(s). You should upload this letter as a separate file labeled 'Response to Reviewers'.
- A marked-up copy of your manuscript that highlights changes made to the original version. You should upload this as a separate file labeled 'Revised Manuscript with Track Changes'.
- An unmarked version of your revised paper without tracked changes. You should upload this as a separate file labeled 'Manuscript'.

If you would like to make changes to your financial disclosure, please include your updated statement in your cover letter. Guidelines for resubmitting your figure files are available below the reviewer comments at the end of this letter.

If applicable, we recommend that you deposit your laboratory protocols in protocols.io to enhance the reproducibility of your results. Protocols.io assigns your protocol its own identifier (DOI) so that it can be cited independently in the future. For instructions see: https://journals.plos.org/plosone/s/submission-guidelines#loc-laboratory-protocols. Additionally, PLOS ONE offers an option for publishing peer-reviewed Lab Protocol articles, which describe protocols hosted on protocols.io. Read more information on sharing protocols at https://plos.org/protocols?utm_medium=editorial-email&utm_source=authorletters&utm_campaign=protocols.

We look forward to receiving your revised manuscript.

Kind regards,

Novel N. Chegou, Ph.D
Academic Editor
PLOS ONE

Journal Requirements:

Please review your reference list to ensure that it is complete and correct. If you have cited papers that have been retracted, please include the rationale for doing so in the manuscript text, or remove these references and replace them with relevant current references. Any changes to the reference list should be mentioned in the rebuttal letter that accompanies your revised manuscript. If you need to cite a retracted article, indicate the article's retracted status in the References list and also include a citation and full reference for the retraction notice.

Thank you for this comment.

We apologize that this requirement was not well addressed previously. We have replaced the retracted articles from the reference list as well as from the citations in the article with the correct ones. We hope this action addresses the concerns raised by the editor regarding the cited papers that have been retracted.

[Note: HTML markup is below. Please do not edit.]

Reviewers' comments:

Reviewer's Responses to Questions

Comments to the Author

1. If the authors have adequately addressed your comments raised in a previous round of review and you feel that this manuscript is now acceptable for publication, you may indicate that here to bypass the "Comments to the Author" section, enter your conflict of interest statement in the "Confidential to Editor" section, and submit your "Accept" recommendation.

Reviewer #1: (No Response)

---

2. Is the manuscript technically sound, and do the data support the conclusions?

The manuscript must describe a technically sound piece of scientific research with data that supports the conclusions. Experiments must have been conducted rigorously, with appropriate controls, replication, and sample sizes. The conclusions must be drawn appropriately based on the data presented.

Reviewer #1: Yes

---

3. Has the statistical analysis been performed appropriately and rigorously?

Reviewer #1: Yes

---

4. Have the authors made all data underlying the findings in their manuscript fully available?

The PLOS Data policy requires authors to make all data underlying the findings described in their manuscript fully available without restriction, with rare exception (please refer to the Data Availability Statement in the manuscript PDF file). The data should be provided as part of the manuscript or its supporting information, or deposited to a public repository. For example, in addition to summary statistics, the data points behind means, medians and variance measures

should be available. If there are restrictions on publicly sharing data—e.g. participant privacy or use of data from a third party—those must be specified.

Reviewer #1: Yes

---

5. Is the manuscript presented in an intelligible fashion and written in standard English?

PLOS ONE does not copyedit accepted manuscripts, so the language in submitted articles must be clear, correct, and unambiguous. Any typographical or grammatical errors should be corrected at revision, so please note any specific errors here.

Reviewer #1: Yes

---

6. Review Comments to the Author

Please use the space provided to explain your answers to the questions above. You may also include additional comments for the author, including concerns about dual publication, research ethics, or publication ethics. (Please upload your review as an attachment if it exceeds 20,000 characters)

Reviewer #1: Thank you for asking me to review this revised manuscript. The authors have done a good job at responding to all comments.

There is only one issue outstanding that they did not address. To confirm convergence and appropriate exploration of the parameter space, the authors should add a table to the supplemental material showing the Rhat statistic and effective sample size for each model parameter.

We regret that this request was missed in our previous responses. In the supplementary material, we have added tables of parameter estimates that include the indicators of model convergence (R hat statistic and effective sample size) for the models we fitted. We hope this addresses the question raised by the reviewer.

---

7. PLOS authors have the option to publish the peer review history of their article (what does this mean?). If published, this will include your full peer review and any attached files.

If you choose “no”, your identity will remain anonymous but your review may still be made public.

Do you want your identity to be public for this peer review? For information about this choice, including consent withdrawal, please see our Privacy Policy.

Reviewer #1: **Yes:** Peter MacPherson

[NOTE: If reviewer comments were submitted as an attachment file, they will be attached to this email and accessible via the submission site. Please log into your account, locate the manuscript record, and check for the action link "View Attachments". If this link does not appear, there are no attachment files.]

While revising your submission, please upload your figure files to the Preflight Analysis and Conversion Engine (PACE) digital diagnostic tool, https://pacev2.apexcovantage.com/. PACE helps

ensure that figures meet PLOS requirements. To use PACE, you must first register as a user. Registration is free. Then, login and navigate to the UPLOAD tab, where you will find detailed instructions on how to use the tool. If you encounter any issues or have any questions when using PACE, please email PLOS at figures@plos.org. Please note that Supporting Information files do not need this step.

In compliance with data protection regulations, you may request that we remove your personal registration details at any time. (Remove my information/details). Please contact the publication office if you have any questions.
